# **β**-Catenin signaling in alveolar macrophages enhances lung metastasis through a TNF-dependent mechanism

**DOI:** 10.1172/jci.insight.160978

**Published:** 2023-04-24

**Authors:** Elliot D. Kramer, Stephanie L. Tzetzo, Sean H. Colligan, Mary L. Hensen, Craig M. Brackett, Björn E. Clausen, Makoto M. Taketo, Scott I. Abrams

**Affiliations:** 1 Department of Immunology and; 2 Department of Cell Stress Biology, Roswell Park Comprehensive Cancer Center, Buffalo, New York, USA.; 3 Institute for Molecular Medicine, Paul Klein Center for Immune Intervention, University Medical Center of the Johannes Gutenberg-University Mainz, Mainz, Germany.; 4 Division of Experimental Therapeutics, Graduate School of Medicine, Kyoto University, Kyoto, Japan.

**Keywords:** Oncology, Breast cancer, Macrophages, Mouse models

## Abstract

The main cause of malignancy-related mortality is metastasis. Although metastatic progression is driven by diverse tumor-intrinsic mechanisms, there is a growing appreciation for the contribution of tumor-extrinsic elements of the tumor microenvironment, especially macrophages, which correlate with poor clinical outcomes. Macrophages consist of bone marrow–derived and tissue-resident populations. In contrast to bone marrow–derived macrophages, the transcriptional pathways that govern the pro-metastatic activities of tissue-resident macrophages (TRMs) remain less clear. Alveolar macrophages (AMs) are a TRM population with critical roles in tissue homeostasis and metastasis. Wnt/β-catenin signaling is a hallmark of cancer and has been identified as a pathologic regulator of AMs in infection. We tested the hypothesis that β-catenin expression in AMs enhances metastasis in solid tumor models. Using a genetic β-catenin gain-of-function approach, we demonstrated that (a) enhanced β-catenin in AMs heightened lung metastasis; (b) β-catenin activity in AMs drove a dysregulated inflammatory program strongly associated with *Tnf* expression; and (c) localized TNF-α blockade abrogated this metastatic outcome. Last, β-catenin gene *CTNNB1* and *TNF* expression levels were positively correlated in AMs of patients with lung cancer. Overall, our findings revealed a Wnt/β-catenin/TNF-α pro-metastatic axis in AMs with potential therapeutic implications against tumors refractory to the antineoplastic actions of TNF-α.

## Graphical abstract

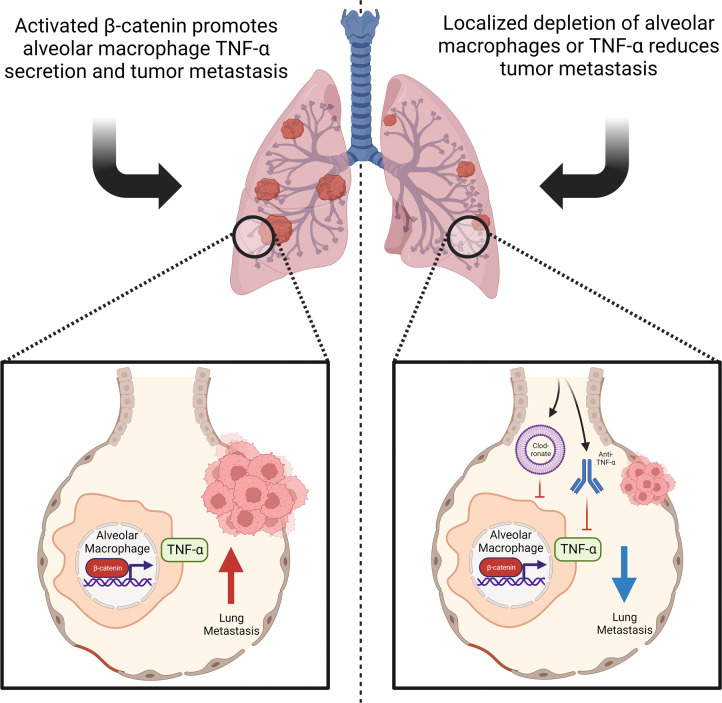

## Introduction

Metastasis remains the leading cause of solid cancer–related deaths worldwide (1). Therefore, understanding the mechanisms that govern tumor progression from the primary tumor to the metastatic stage is essential for the identification of novel therapeutic targets. The means by which this progression occurs can be broadly separated into tumor-intrinsic and tumor-extrinsic mechanisms (2). Primary tumor tissue represents a heterogenous milieu of malignant cells, characterized by a reversion from differentiated cells to a stem cell–like, proliferative, and invasive state (3). The transformation from normal to neoplastic biology is initially driven by genetic and epigenetic events that alter the expression of oncogenes and tumor suppressor genes within somatic cells at the tissue site of origin. Over the past decades, much work has been devoted to identifying specific driver mutations that promote tumorigenesis, and these fundamental discoveries have been fruitful, resulting in the identification of druggable targets including tyrosine kinase receptors, KRAS mutants, hormone receptors, cell cycle components, and epigenetic regulators (4–6).

However, the mechanisms that control tumor progression extend beyond the malignant cells themselves to include multiple stromal and immune cell components of the tumor microenvironment (TME). This diverse collection of nonmalignant cells is capable of promoting tumor progression via varied immune-suppressive, angiogenic, and growth factor–mediated mechanisms (7). In patients with breast cancer and melanoma, for example, macrophages have been identified as the most abundant leukocyte subset of the TME (8), suggesting an integral role for macrophages in these solid tumors. These tumor-associated macrophages (TAMs) contribute to tumor growth, as well as the immunosuppressive TME (9).

Like primary tumor growth, tumor progression to metastasis is mediated by both tumor-intrinsic and -extrinsic factors (10). Although the tumor-intrinsic mechanisms of metastatic progression have been extensively explored elsewhere (11), the mechanisms underlying how immune and myeloid cells at distal sites enable metastasis are less clear. Macrophages are of particular interest as a cancer cell–nonautonomous driver of metastasis, since they are found in nearly every tissue of the body at steady state and have been proposed to play important roles in conditioning the pre-metastatic niche for tumor spread and colonization (12). This concept is in line with the original “seed and soil” hypothesis put forth by Stephen Paget at the end of the 19th century (13). Indeed, several studies have demonstrated distinct transcriptional differences between homeostatic macrophages and those found at the same tissues in primary tumors and distal metastatic sites alike (14, 15). Furthermore, although TAMs in both primary and metastatic sites have historically been studied as a monolithic myeloid subset, recent advances in single-cell transcriptomic analyses have identified significant functional and ontological heterogeneity between and within the self-renewing tissue-resident and the recruited monocyte-derived TAM populations (16–18).

Bone marrow–derived monocytes are known to traffic along chemokine gradients generated by the TME and differentiate into TAMs that are generally rendered pro-tumorigenic within the TME (19). Many individual TME-derived factors have been associated with altering macrophage function; however, these changes are thought to result from a broader pro-tumorigenic program directed by transcription factors. For example, interferon regulatory factor-8 deficiency has been associated with a pro-metastatic program in lung macrophages, defined by increased expression of cell migration and neutrophil chemotactic gene signatures (20). Furthermore, activation of STAT3/6 signaling and HIF-1α expression within TAMs have been implicated in angiogenesis, tumor invasion, and immunosuppression (21). Despite our knowledge about bone marrow–derived macrophages (BMDMs) within primary TMEs, considerably less is known about the mechanisms governing tumor-induced alterations in the function of tissue-resident macrophages (TRMs). This TRM-tumor interplay is particularly relevant in the context of metastasis given that TRMs, namely microglia and alveolar macrophages (AMs), are the predominant macrophage subsets in the brain and lung, respectively, which are common sites of metastasis for multiple solid cancer types (22).

Although our understanding of the transcriptional regulators of the TRM response in metastasis remains incomplete, Wnt/β-catenin signaling has been implicated as a potential mediator of tumor-driven macrophage dysfunction (23). Wnt/β-catenin signaling is evolutionarily highly conserved, and the β-catenin gene, *Ctnnb1*, is expressed in many tissues (24). β-Catenin activity is tightly regulated posttranslationally through phosphorylation, ubiquitination, and degradation (25). Tumor-intrinsic driver mutations, wherein genetic alterations uncouple β-catenin regulation, lead to constitutive β-catenin activation and have long been known to drive neoplastic transformation (26). With respect to macrophages, it has been shown that macrophage-derived Wnt7b ligand secretion promotes breast cancer growth and metastasis through angiogenesis (27). Additional studies show that β-catenin activity in total lung macrophages influences primary lung cancer progression (28, 29). Furthermore, β-catenin, in conjunction with an HIF-1α cofactor, has been identified as a key transcriptional regulator of AM proliferation and pathologic inflammation in the context of severe COVID-19 infection (30). Such inflammatory networks may also contribute to cancer since uncontrolled chronic inflammation is a hallmark of progressive malignancy (31).

While these studies elegantly connect Wnt/β-catenin signaling to dysregulation of lung macrophage biology, the precise roles of this signaling pathway within AMs in driving or enhancing lung metastasis remain unresolved (32, 33). Here, we revealed and underscored the importance of intrinsic β-catenin signaling in tissue-resident AMs as an enhancer of metastasis in preclinical models of triple-negative breast cancer (TNBC) and melanoma. Moreover, we demonstrated that β-catenin signaling in tissue-resident AMs augmented lung metastasis through a TNF-α–dependent mechanism. Last, we correlated *CTNNB1* and *TNF* expression in the AMs of patients with cancer, highlighting the translational relevance of our findings. Indeed, TNF-α is a complex cytokine and has been shown to elicit both antitumor and pro-tumor activities depending on tumor-intrinsic characteristics (34). Therefore, in settings where tumors are resistant to the cytotoxic actions of TNF-α, TNF-α may deliver opposing survival or proliferative signals (35). Thus, our findings also uncovered a mechanistic basis for TNF-α enhancement in the metastatic microenvironment and a therapeutically targetable axis to potentially mitigate metastatic development or outgrowth.

## Results

### Myeloid cell–specific constitutive β-catenin activity enhances primary tumor growth and spontaneous lung metastasis in a preclinical model of TNBC.

**Figure 1:**
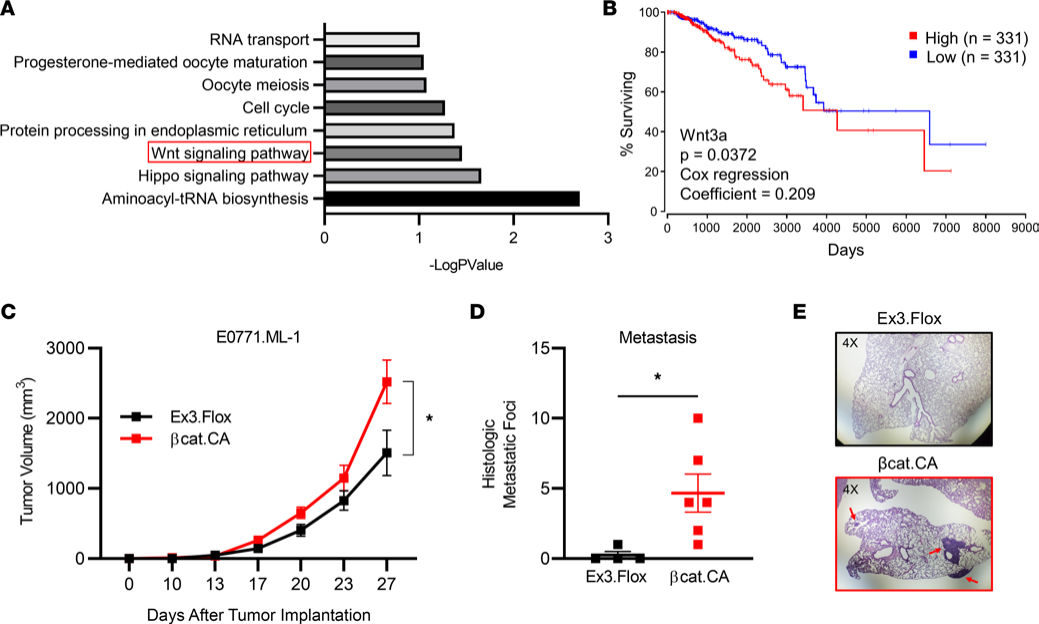
β-Catenin constitutive activation in myeloid cells promotes primary tumor growth and spontaneous lung metastasis in the E0771.ML-1 model. (**A**) Kyoto Encyclopedia of Genes and Genomes pathway analysis of genes associated with poor overall survival in breast cancer patients from TCGA database, identified through OncoLnc by positive Cox regression score. (**B**) Correlation between high and low Wnt3a-expressing breast cancer and OS, based on upper and lower tertile cutoffs. (**C**) Primary tumor growth of orthotopically implanted E0771.ML-1 tumor cells in β-catenin–activated C57BL/6 *Lyz2-Cre^+/+^* *Ctnnb1*^Ex3Δ*/*Δ^ (βcat.CA) or *Ctnnb1*^Ex3Δ*/*Δ^ floxed control (Ex3.Flox) C57BL/6 mice. (**D**) Spontaneous histologic metastatic foci of E0771.ML-1 tumor–bearing mice at endpoint. (**E**) H&E-stained lung sections from mice in **D** representative of 2 independent experiments; red arrows highlight E0771.ML-1 metastatic foci at 4× original magnification. (**C** and **D**) The mean ± SEM values are shown and represent 4–6 individual mice. Statistical analysis is based on 2-way ANOVA (**C**) and 2-tailed *t* test (**D**); * = *P* < 0.05.

Aberrant activation of Wnt signaling is a known hallmark of cancer, and the β-catenin protein is the central signal transducer within that pathway (36). Further substantiating the putative role for Wnt/β-catenin signaling specifically in the context of breast cancer, RNA expression data from bulk breast cancer tumor samples from The Cancer Genome Atlas (TCGA) demonstrated that Wnt signaling correlated with decreased overall survival in patients with breast cancer (Figure 1A). Furthermore, differential expression of the canonical Wnt ligand Wnt3a was correlated with decreased overall survival across all breast cancer subtypes (Figure 1B). It is important to note, however, that these findings are limited by the bulk nature of the expression data, which only serves as an overall indication and masks the potential individual contributions of the multiple cell types within the TME.

Recent work demonstrated that the loss of β-catenin in myeloid cells decreases tumor growth in multiple preclinical models of lung cancer (29). To expand upon these findings, we used a myeloid specific β-catenin gain-of-function approach in which β-catenin is stabilized via conditional deletion of the exon 3 (Ex3Δ) regulatory domain (37). We crossed C57BL/6 mice expressing *Ctnnb1*^Ex3 Δ/ Δ^ with those expressing a Cre-recombinase driven by the *Lyz2* promoter (*Lyz2-Cre*^+/+^) (Supplemental Figure 1, A and B; supplemental material available online with this article; https://doi.org/10.1172/jci.insight.160978DS1) (38). In this *Lyz2*-*Cre*^+/+^ *Ctnnb1*^Ex3*Δ/*Δ^ model (βcat.CA), active β-catenin (ABC) levels were enhanced relative to the floxed controls (Ex3.Flox) in CD45^+^ cells, based on nonphosphorylated active β-catenin expression, but not in CD45^–^ cells (Supplemental Figure 1, C and D), consistent with cell-specific expression of the *Lyz2* promoter. To test the hypothesis that activated β-catenin signaling intrinsic to myeloid cells facilitates TNBC progression, we orthotopically implanted the metastatic TNBC cell line E0771.ML-1 into syngeneic C57BL/6 βcat.CA or Ex3.Flox mice. In line with our hypothesis, we observed an increased rate of E0771.ML-1 growth in βcat.CA mice (Figure 1C). These findings were verified by an increase in the weight of E0771.ML-1 tumors from βcat.CA hosts collected at endpoint (Supplemental Figure 1E).

Splenomegaly is a marker of tumor-induced extramedullary myelopoiesis and/or trafficking of myeloid cells to the organ (39); however, we observed no significant difference in spleen weight between the genotypes (Supplemental Figure 1F). Interestingly, while few metastatic foci were in the lungs of the Ex3.Flox mice, there was a significant increase in metastasis in βcat.CA mice (Figure 1D). These metastatic foci could be readily seen in histological sections of the βcat.CA lungs (Figure 1E). Lung tissue harbors multiple myeloid populations known to affect metastasis, including AMs and interstitial macrophages, and, therefore, such macrophage populations may be dysregulated in the βcat.CA model. Although it has been reported that *Lyz2* is expressed in type II alveolar epithelial cells, we only observed an increase in nonphosphorylated ABC in the CD45^+^ leukocyte compartment of βcat.CA lungs (Supplemental Figure 1D) (40). Taken together, these findings extend the pro-tumorigenic role of myeloid cell–intrinsic β-catenin activity to a preclinical model of TNBC and unveil a role for myeloid cell–specific β-catenin signaling in enhancing metastasis to the lung.

### Lyz2-Cre–mediated β-catenin gain of function in a heterozygous reporter model maintains enhanced tumor growth and metastatic phenotype.

While our data established a causal role for constitutively active myeloid β-catenin in promoting tumor growth and metastasis, the specific myeloid cell type(s) responsible for these outcomes remained unclear. *Lyz2*, the promoter driving Cre-recombinase in our system, is known to be expressed by multiple myeloid cell populations (41). To address this potential model limitation, we crossed *Lyz2-Cre*^+/+^ *ROSA26-EYFP^+/+^* reporter mice, which express yellow fluorescent protein (YFP) in cells with *Lyz2*-*Cre* activity, to our βcat.CA mouse (42). This mating yielded triple-transgenic *Lyz2-Cre*^+/+^ *Ctnnb1*^Ex3Δ/wt^ *ROSA26-EYFP^+/–^* (*Lyz2-Cre^+/+^* *YFP^+/–^* *Ctnnb1*^Ex3*Δ*/wt^) progeny that were homozygous for the *Lyz2* Cre-recombinase but heterozygous for the *EYFP* reporter and constitutively active β-catenin *Ctnnb1* (*Ex3*Δ) alleles (Supplemental Figure 1, G and H). *Lyz2-Cre^+/+^* *ROSA26-EYFP^+/+^* mice were also bred to *Lyz2-Cre^+/+^* mice to yield *Lyz2-Cre^+/+^* *ROSA26-EYFP^+/–^* (*Lyz2-Cre^+/+^* *YFP^+/–^*) control mice that would express the same intensity of the YFP Cre-reporter signal but lack β-catenin activation. These *YFP* models enabled us to make one-to-one comparisons between myeloid cells expressing constitutively active or WT *Ctnnb1* alleles, respectively, without the potential confounding variable of *Lyz2* penetrance.

**Figure 2:**
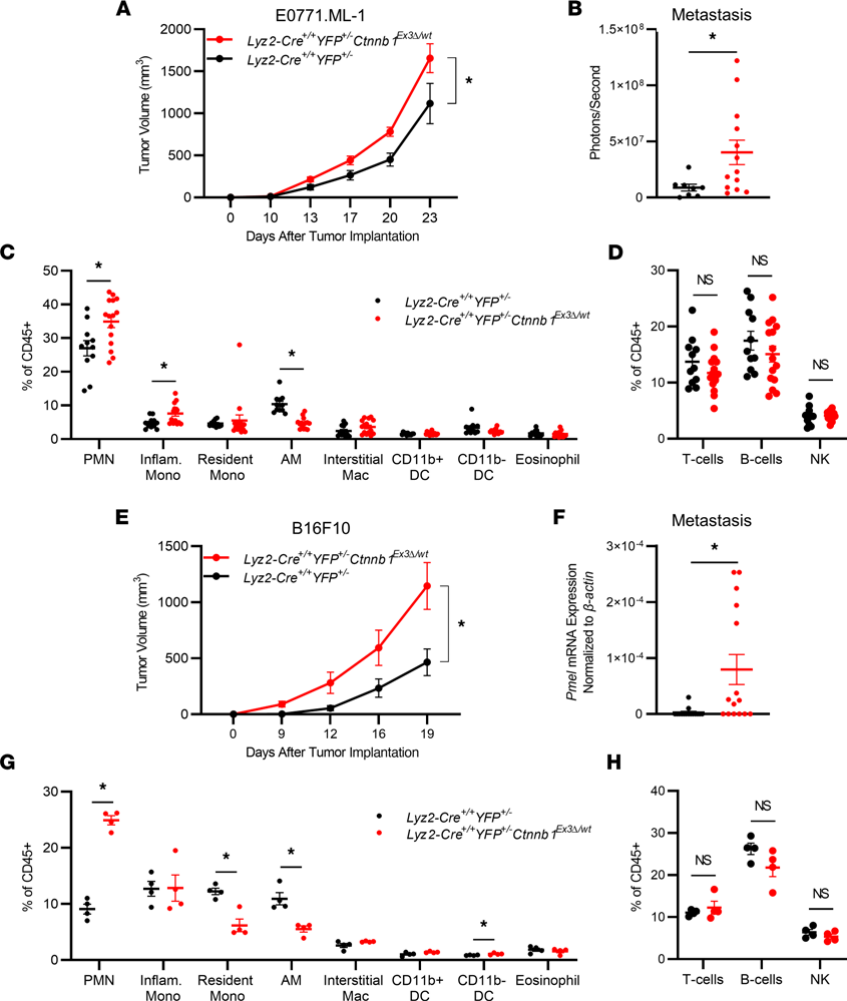
Heterozygous β-catenin gain of function in *Lyz2*-Cre reporter mice maintains an enhanced tumor growth and metastatic phenotype in TNBC and melanoma models. (**A**) Primary tumor growth of orthotopically implanted E0771.ML-1 tumor cells in *Lyz2-Cre^+/+^* *YFP^+/–^* *Ctnnb1*^Ex3Δ/wt^ and *Lyz2-Cre^+/+^* *YFP^+/–^* C57BL/6 mice. (**B**) Bioluminescence of ex vivo–imaged lungs in **A**. (**C**) Proportion of myeloid and (**D**) lymphoid populations from dissociated whole lungs from **A**, gated as described in Supplemental Figure 2D and labeled as in **C**. (**E**) Primary tumor growth of orthotopically implanted B16F10 cells in *Lyz2-Cre^+/+^* *YFP^+/–^* *Ctnnb1*^Ex3Δ/wt^ and *Lyz2-Cre^+/+^* *YFP^+/–^* C57BL/6 mice. (**F**) mRNA expression of *Pmel* at endpoint from digested lungs from **E** as labeled. (**G**) Proportion of myeloid and (**H**) lymphoid populations from dissociated whole lungs from **E**; data are representative of 2 independent experiments, gated as in Supplemental Figure 2D. (**A**–**H**) In all panels the mean ± SEM values are shown and represent 8–15 (**A**–**D**), 13–15 (**E** and **F**), or 4 (**G** and **H**) individual mice. Statistical analysis is based on 2-way ANOVA (**A** and **E**) and 2-tailed *t* tests (**B**–**D** and **F**–**H**); * = *P* < 0.05.

Despite heterozygous expression of the transgene, *Lyz2-Cre^+/+^* *YFP^+/–^* *Ctnnb1*^Ex3*Δ*/wt^ mice showed increased E0771.ML-1 growth relative to the controls (Figure 2A and Supplemental Figure 2A). The immune landscape of the primary TME is known to play a key role in mediating tumor progression; therefore, we explored changes in the TME immune contexture through a comprehensive flow cytometry gating strategy (Supplemental Figure 2B). We observed an increase in CD11b^+^Ly6G^+^ polymorphonuclear cells (PMNs) and a concordant decrease in CD11b^+^CD11c^+^ dendritic cells (DCs) within the myeloid fraction of the CD45^+^ leukocytes (Supplemental Figure 2C, left). PMN-like cells that share this marker combination could include PMN–myeloid-derived suppressor cells (PMN-MDSCs), tumor-associated neutrophils, or both, which have known immune suppressive roles (43). Additionally, there was a significant decrease in both CD4^+^ and CD8^+^ tumor-infiltrating lymphocytes (Supplemental Figure 2C, right). Thus, there was a shift toward a more immune-suppressive phenotype in the primary E0771.ML-1 TME in mice expressing myeloid cell–specific constitutive β-catenin activity.

Increased lung metastasis was also maintained in *Lyz2-Cre*^+/+^ *YFP^+/–^* *Ctnnb1*^Ex3*Δ*/wt^ mice (Figure 2B). To better characterize the metastatic lung microenvironment, we prepared single-cell suspensions of the entire lung tissue and analyzed the myeloid cell compartment using a previously published and validated comprehensive flow cytometry panel (Supplemental Figure 2D) (44). We observed that constitutive activation of β-catenin in myeloid cells led to the accumulation of PMN-like cells and Ly6G^–^MHCII^–^SSC^lo^CD11b^hi^CD64^int^Ly6C^hi^ inflammatory monocytes at the expense of the tissue-resident AM population (Figure 2C). Interestingly, studies elsewhere revealed a similar decrease in AM abundance in the context of viral infection using the βcat.CA model, which was attributed to β-catenin–induced cell cycle arrest specifically in AMs (30). Those findings, together with ours, demonstrate the negative effects of enhanced myeloid β-catenin expression on AM abundance. In contrast, we observed no significant differences in the relative abundance of the lymphoid components of the lung environment (Figure 2D).

Malignant melanoma is another highly metastatic cancer that is known to target the lung (33). Therefore, we expanded our analysis to the B16F10 mouse model of metastatic melanoma (45). As with the E0771.ML-1 model, we observed increased tumor growth and mass in *Lyz2-Cre*^+/+^ *YFP^+/–^* *Ctnnb1*^Ex3*Δ*/wt^ mice (Figure 2E and Supplemental Figure 2E, left). This increased tumor growth was accompanied by an increase in PMNs and a decrease in CD11b^+^ DCs in the primary TME, consistent with a heightened immune-suppressive state (Supplemental Figure 2E, middle). This is in accordance with the E0771.ML-1 model; however, there was no significant change in the frequency of T cell subsets (Supplemental Figure 2E, right). Additionally, we observed a difference in B16F10 metastasis between the genotypes as determined by mRNA expression of the melanocyte-specific gene premelanosome protein (*Pmel*) in whole-lung lysates (Figure 2F) (46). While there was a robust increase in PMNs and a decrease in AMs within the B16F10 lung microenvironment, there was no significant change in the inflammatory monocyte subset. Instead, we observed a decrease in the Ly6G^–^MHCII^–^SSC^lo^CD11b^hi^CD64^int^Ly6C^lo/–^CD11c^int^ resident monocyte population (Figure 2G). As with the E0771.ML-1 model, no significant differences in the relative abundance of the lymphoid components of the lung environment were observed (Figure 2H), which contrasted with the primary TME, further suggesting a myeloid driven tumor-supportive mechanism in the metastatic lung. Last, using a third tumor model, luciferase-expressing Lewis lung carcinoma (LLC), we demonstrated increased tumor growth and metastasis in mice with enhanced β-catenin expression relative to the controls (Supplemental Figure 2, F and G).

To better understand the myeloid cell–specific effects of constitutively active β-catenin in the *Lyz2*-Cre model, we quantified the penetrance of Cre-recombinase in E0771.ML-1-bearing mice using the YFP Cre-reporter. Interestingly, while PMN cell numbers were significantly increased in the primary TME of *Lyz2-Cre*^+/+^ *YFP^+/–^* *Ctnnb1*^Ex3*Δ*/wt^ mice, only about 20% of PMNs expressed the YFP Cre-reporter. On the contrary, approximately half of all CD11b^hi^Ly6G^–^Ly6C^+^ monocytes, CD11b^hi^Ly6G^–^Ly6C^lo^F4/80^+^ macrophages, and CD11b^+^ DCs expressed the YFP Cre-reporter (Supplemental Figure 2H). This suggested that the effect of β-catenin activation in the βcat.CA and *Lyz2-Cre*^+/+^ *YFP^+/–^* *Ctnnb1*^Ex3*Δ*/wt^ models on PMNs in the primary TME could be indirect, potentially mediated by chemokines secreted by other myeloid subsets. Alternatively, within the metastatic lung environment, there was broad expression of the Cre-reporter across multiple myeloid subsets. Strikingly, AMs had nearly complete penetrance (Supplemental Figure 2I and inset), suggesting a prominent role for AMs in the metastatic process in βcat.CA and *Lyz2-Cre*^+/+^ *YFP^+/–^* *Ctnnb1*^Ex3*Δ*/wt^ models.

### Increased metastasis in mice with myeloid specific constitutive β-catenin activity is independent of primary tumor growth but dependent on AMs.

To test for a causal relationship between activated β-catenin in AMs and enhanced metastasis, we specifically depleted this macrophage population in vivo by intranasal administration of clodronate liposomes, as described by Burkard-Mandel et al. (47). This approach does not significantly deplete interstitial and systemic macrophage populations. AMs are the most abundant phagocyte within the lung airspaces across multiple mammals, including rodents and humans (48). In non–tumor-bearing *Lyz2-Cre*^+/+^ *YFP^+/–^* control mice, intranasal clodronate administration selectively depleted AMs. This AM depletion led to a reciprocal increase in the absolute number of PMNs, interstitial macrophages, and CD11b^+^ DCs in response to the dysregulation caused by the loss of homeostatic AMs (Supplemental Figure 3, A and B). Since primary tumor size may correlate with the extent of metastasis, we used an experimental metastasis model to exclude the confounding effects of differential primary tumor sizes. Importantly, tail vein injection of B16F10 cells into *Lyz2-Cre*^+/+^ *YFP^+/–^* *Ctnnb1*^Ex3*Δ*/wt^ mice resulted in an increase in metastatic nodules and lung weights compared with the controls (Supplemental Figure 3, C–E), consistent with our spontaneous metastasis data (Figure 2F).

**Figure 3:**
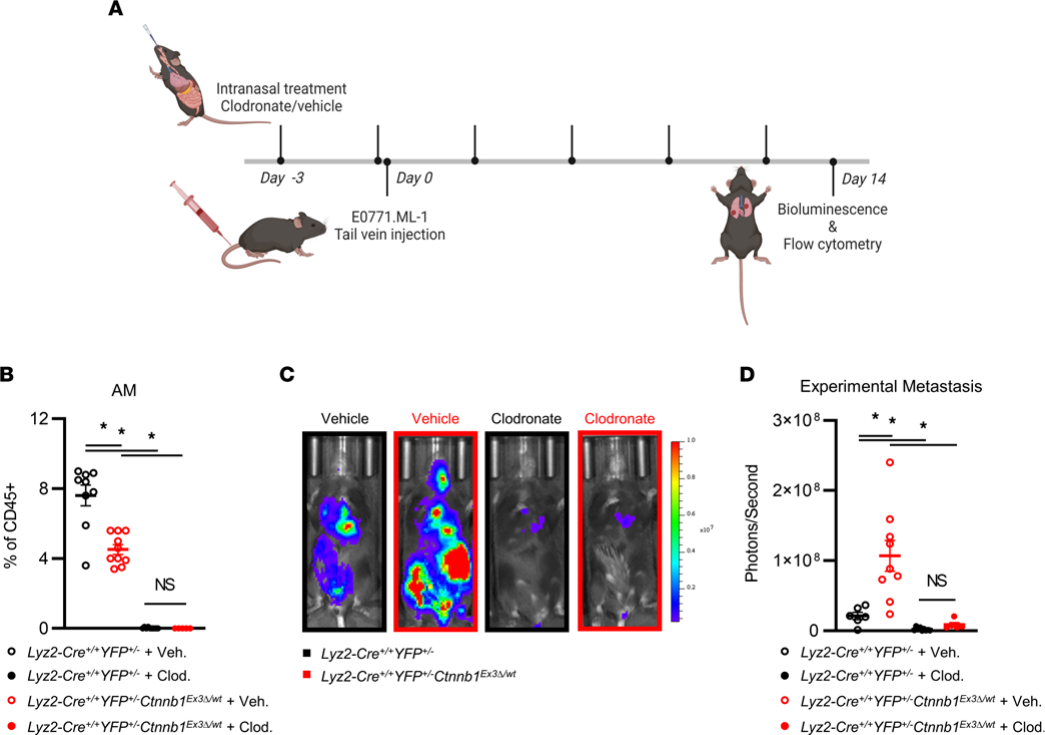
Targeted AM depletion ameliorates genotype-specific metastatic phenotype in a primary tumor-independent manner. (**A**) Treatment schedule for *Lyz2-Cre^+/+^* *YFP^+/–^* *Ctnnb1*^Ex3Δ/wt^ and *Lyz2-Cre^+/+^* *YFP^+/–^* mice treated with clodronate liposomes or vehicle control until endpoint 14 days after tail vein injection of E0771.ML-1 cells. (**B**) Relative abundance of lung CD45^+^Ly6G^–^CD24^–^CD64^+^CD11b^lo^CD11c^+^ AMs at endpoint as described in **A**. (**C**) Bioluminescence images, representative of 3 independent experiments, and (**D**) quantification of mice represented in **C** at endpoint. (**B** and **D**) The mean ± SEM values are shown and represent 5–9 individual mice. Statistical analysis is based on 1-way ANOVA with the Holm-Šídák correction applied for multiple comparisons (**B** and **D**); * = *P* < 0.05.

For AM depletion, clodronate or vehicle control treatment was delivered intranasally 3 days prior to as well as on the day of tail vein injection of E0771.ML-1 and repeated 5 additional times over the course of 14 days (Figure 3A). This treatment schedule led to a near-complete depletion of CD45^+^Ly6G^–^CD24^–^CD64^+^CD11b^lo^CD11c^+^ AMs, as a proportion of CD45^+^ leukocytes within the lung (Figure 3B). Notably, the significant decrease in AMs from *Lyz2-Cre*^+/+^ *YFP^+/–^* *Ctnnb1*^Ex3Δ/wt^ mice bearing E0771.ML-1 experimental metastasis mirrored the findings observed in the spontaneous metastatic setting (Figure 2B). Furthermore, *Lyz2-Cre*^+/+^ *YFP^+/–^* *Ctnnb1*^Ex3*Δ*/wt^ mice bore a higher experimental metastatic burden when compared with the controls, and importantly, local depletion of AMs from the lung environment not only significantly decreased tumor burden but also eliminated the genotype-specific effects (Figure 3, C and D). Thus, these data indicated that AMs enhanced experimental metastasis to the lung and AM depletion abrogated their effect in this *Lyz2*-Cre–driven β-catenin gain-of-function model.

### Constitutively active β-catenin in tissue-resident AMs drives a TNF-mediated pro-metastatic inflammatory program.

Given that constitutive β-catenin activity in AMs enhances metastasis to the lung, we sought to uncover the effector pathways underlying that phenotype. Since we observed that constitutive β-catenin activation decreased the overall abundance of AMs in the lung TME, the mechanisms affecting AMs were unlikely quantitative but rather qualitative in nature. Therefore, we explored changes in the transcriptional program of AMs via bulk RNA-Seq. CD45^+^Ly6G^–^SSC^hi^CD11c^+^Lyz2-YFP^hi^ AMs were isolated by flow sorting from single-cell suspensions of non–tumor-bearing whole lungs collected from *Lyz2-Cre*^+/+^ *YFP^+/–^* *Ctnnb1*^Ex3*Δ*/wt^ or *Lyz2-Cre*^+/+^ *YFP^+/–^* control mice in biologic triplicates (Supplemental Figure 4A).

**Figure 4:**
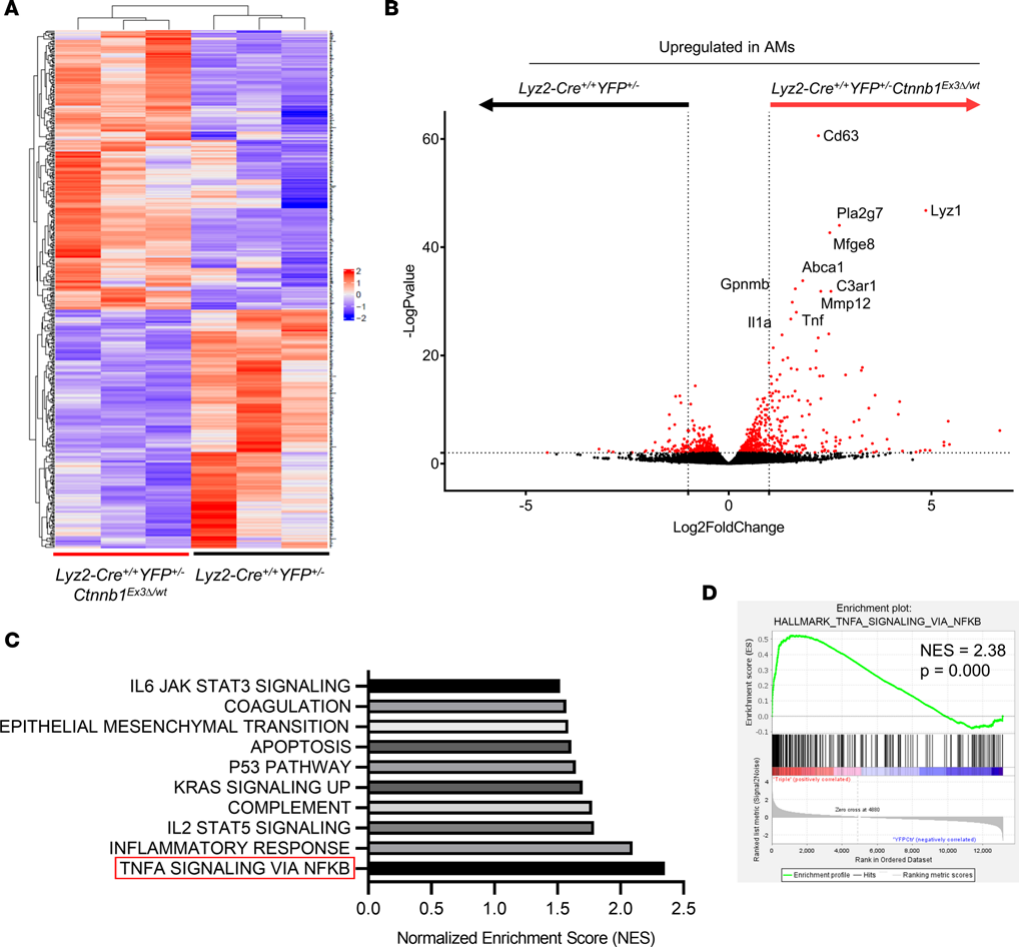
Comparative transcriptomic analysis of the activated β-catenin transcriptional program in AMs. (**A**) Heatmap of top 500 DEGs in CD45^+^Ly6G^–^SSC^hi^CD11c^+^Lyz2-EYFP^+^ AMs flow-sorted from the single-cell–dissociated lungs of non–tumor-bearing *Lyz2-Cre^+/+^* *YFP^+/–^* *Ctnnb1*^Ex3Δ/wt^ and *Lyz2-Cre^+/+^* *YFP^+/–^* mice, as described in Supplemental Figure 4A; *n* = 3, Wald’s test with Benjamini-Hochberg correction. (**B**) DEGs in AMs of *Lyz2-Cre^+/+^* *YFP^+/–^* *Ctnnb1*^Ex3Δ/wt^ and *Lyz2-Cre^+/+^* *YFP^+/–^* mice, cutoff of –log*P* ≥ 2 and 1 ≤ log_2_FC ≤ –1 highlighted in red and top 10 genes upregulated in *Lyz2-Cre^+/+^* *YFP^+/–^* *Ctnnb1*^Ex3Δ/wt^ relative to *Lyz2-Cre^+/+^* *YFP^+/–^* mice labeled. (**C**) Ranked normalized enrichment scores (NES) from Gene Set Enrichment Analysis (GSEA) of Hallmark gene sets significantly upregulated in AMs of *Lyz2-Cre^+/+^* *YFP^+/–^* *Ctnnb1*^Ex3Δ/wt^ relative to *Lyz2-Cre^+/+^* *YFP^+/–^* mice; NES ≥ 2 are highlighted. (**D**) GSEA plot for HALLMARK_TNFA_SIGNALING_VIA_NFKB gene set from **C**.

Since the AMs from both genotypes shared the same cell surface markers and *Lyz2*-YFP reporter expression, the only difference between the groups was the presence or absence of the *Ctnnb1*^Ex3*Δ*/wt^ gain-of-function allele. In total, there were 352 significantly differentially expressed genes (DEGs) between the 2 genotypes, with the majority (75%) being upregulated in AMs of *Lyz2-Cre*^+/+^ *YFP^+/–^* *Ctnnb1*^Ex3*Δ*/wt^ mice (Figure 4, A and B). Principal component analysis revealed little variance within the biologic replicates (PC2) but considerable variance between genotypes (PC1) (Supplemental Figure 4C). Highly DEGs included the chemokines, *Ccl2*, *Ccl3*, *Ccl9*, *Cxcl1*, *Cxcl12*, and *Cxcl3*; growth factors, *Csf1*, *Igf1*; metalloproteases, *Mmp8* and *Mmp12*; and cytokines, *Il1a*, *Il1b*, *Il10*, and *Tnf* (Supplemental Table 1). The most significantly upregulated genes in *Lyz2-Cre*^+/+^ *YFP^+/–^* *Ctnnb1*^Ex3*Δ*/wt^ AMs relative to the controls were *Cd63*, *Lyz1*, *Pla2g7*, *Mfge8*, *Abca1*, *Gpnmb*, *C3ar1*, *Mmp12*, *Tnf*, and *Il1a* (Figure 4B).

We hypothesized that β-catenin–activated AMs secreted effector molecules, which could interact directly with metastatic tumor cells themselves or with immune and stromal components of the lung microenvironment. Therefore, we focused on secreted proteins in our analyses, of which *Tnf* was one of the most significantly upregulated cytokines in the β-catenin–activated AMs (Figure 4C). Furthermore, TNFA SIGNALING VIA NFKB was the most enriched gene set within the significantly upregulated genes in AMs of *Lyz2-Cre*^+/+^ *YFP^+/–^* *Ctnnb1*^Ex3Δ/wt^ mice, as assessed by gene set enrichment analysis (GSEA) (Figure 4, C and D; Supplemental Figure 4D; and Supplemental Tables 2 and 3). Interestingly, the highly enriched INFLAMMATORY RESPONSE gene set contained multiple upregulated myeloid chemokines, including the previously noted *Ccl2*, *Ccl9*, *Cxcl1*, *Cxcl2*, and *Cxcl3* genes, concurrent with the increase in PMN infiltration of the metastatic lung we observed by flow cytometry (Figure 2, C and G).

To account for any redundancy in our Hallmark gene signatures caused by the same DEGs being enriched in multiple gene sets, we performed Gene Ontology (GO) clustering using Metascape (49). With the exceptions of the tissue remodeling and neutrophil degranulation pathways, there was substantial overlap between GO terms, indicating a broadly shared DEG signature across multiple pathways (Supplemental Figure 4E). These data indicated that constitutively activated β-catenin in AMs led to increased *Tnf* expression. To further strengthen the findings based on our genetic approach, we leveraged publicly available single-cell RNA-Seq (scRNA-Seq) data of murine AMs from Zhu et al. (National Center for Biotechnology Information Gene Expression Omnibus [GEO] GSE164793) that used a pharmacological approach to activate β-catenin (30). In their study, AMs were treated in vivo via intranasal recombinant Wnt3a ligand and then were isolated by bronchoalveolar lavage (BAL) for single-cell transcriptomic analysis. We verified their reported finding that untreated and Wnt3a-treated BAL AMs formed distinct clusters following dimensional reduction and uniform manifold approximation and projection analyses (Supplemental Figure 4F, left). Additionally, on a per-cell basis, *Tnf* expression was greater in Wnt3a-treated AMs relative to the untreated controls (Supplemental Figure 4F, right) and was significantly increased between the treatment populations (Supplemental Figure 4G). Therefore, β-catenin activation in AMs led to a distinct transcriptional program enriched for inflammatory, vascular development, and cytokine/chemotactic pathways important for metastasis. Specifically, an increase in *Tnf* expression was observed in the context of both β-catenin activation by genetic gain of function and Wnt ligand treatment.

### Neonatal liver-derived macrophages recapitulate the AM transcriptional landscape and are distinct from BMDMs.

AMs are the most abundant immune cell in the alveoli at steady sate; however, their total numbers within an individual mouse are low and represent a small proportion of the total lung tissue (44). Therefore, we sought to establish an in vitro primary cell culture model to further dissect the mechanistic role of enhanced β-catenin expression within these cells. AMs are transcriptionally distinct from both macrophages replenished from bone marrow–derived monocytes, as well as interstitial TRMs in the lung (18). Indeed, in mice, AMs are derived from yolk sac and fetal liver progenitors early on in development and self-renew in situ supported by GM-CSF paracrine signaling (50, 51). Therefore, to probe the impact of β-catenin activation on AMs more rigorously in vitro, we opted to establish a primary culture system with comparable ontology and growth signals termed neonatal liver-derived macrophages (NLDMs), modified from the Max Planck Institute cell line developed by Fejer et al. (52).

To that end, we derived primary NLDMs by culturing dissociated neonatal livers in 30 ng/mL GM-CSF for 14 days prior to passaging and analyses. NLDMs derived from *Lyz2-Cre*^+/+^ *YFP^+/–^* control mice had high *Lyz2*-YFP penetrance when viewed by fluorescence microscopy (Supplemental Figure 5A) and phenotypically characterized by flow cytometry (Supplemental Figure 5B). NLDMs expressed increased levels of the TRM markers *Timd4* and *Lyve1* when compared with M-CSF–cultured BMDMs; however, there was no difference in *Adgre1* expression, which codes for the murine pan-macrophage marker F4/80 (Supplemental Figure 5C). NLDMs expressed significantly higher levels of the well-described AM marker *Siglecf* compared with BMDMs, as well as the AM-specific markers *Plet1*, *Fabp5*, and *Net1* recently identified by comprehensive scRNA-Seq studies of murine tissues (Supplemental Figure 5D) (18).

**Figure 5:**
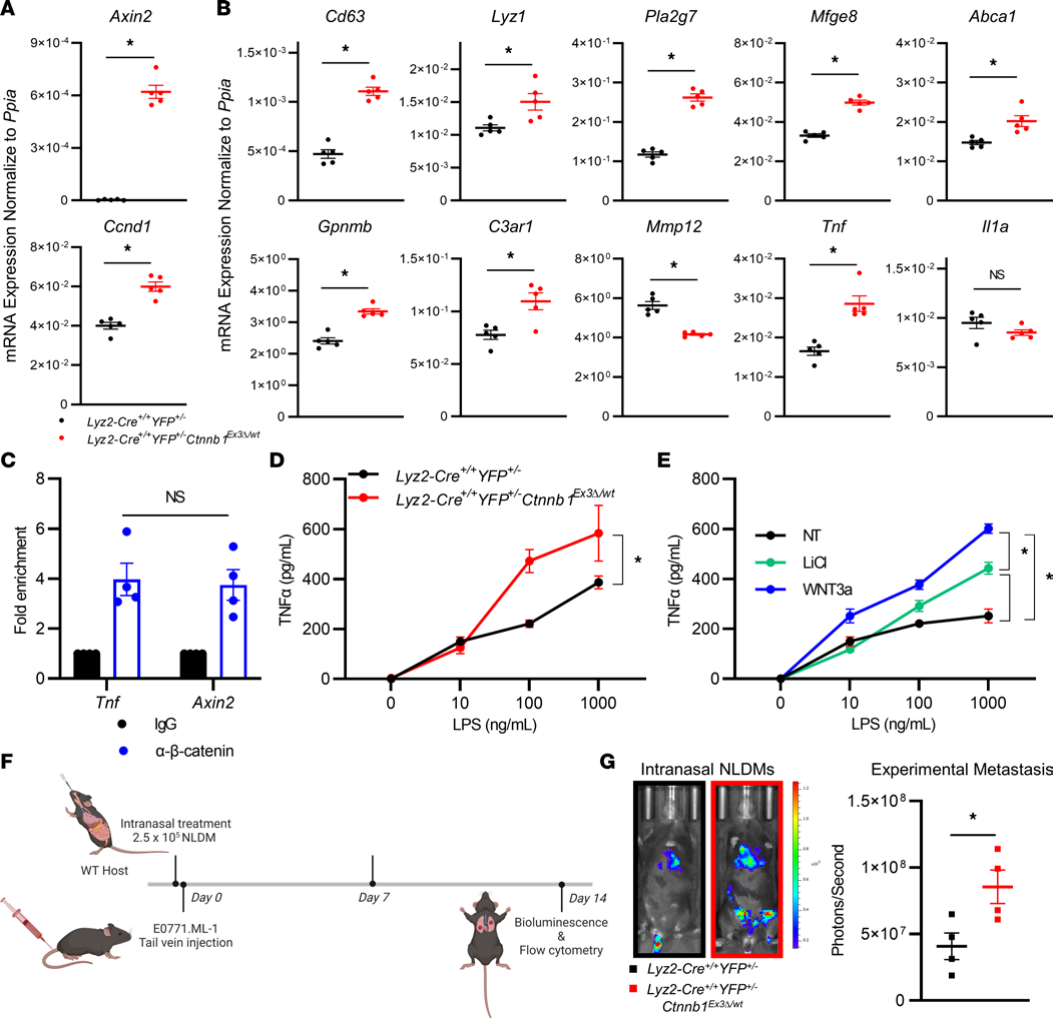
β-Catenin activity in NLDMs drives *Tnf* expression, and intranasal adoptive transfer of NLDMs with constitutively active β-catenin increases experimental metastasis. (**A**) *Lyz2-Cre^+/+^* *YFP^+/–^* *Ctnnb1*^Ex3Δ/wt^ and *Lyz2-Cre^+/+^* *YFP^+/–^* NLDM reverse transcription quantitative PCR (RT-qPCR) of *Axin2* and *Ccnd1*. (**B**) NLDMs derived from *Lyz2-Cre^+/+^* *YFP^+/–^* *Ctnnb1*^Ex3Δ/wt^ and *Lyz2-Cre^+/+^* *YFP^+/–^* mice analyzed by RT-qPCR for the top 10 upregulated DEGs identified in Figure 4B: *Cd63*, *Lyz1*, *Pla2g7*, *Mfge8*, *Abca1*, *Gpnmb*, *C3ar1*, *Mmp12*, *Tnf*, and *Il1a*. (**C**) CUT&RUN assay assessed by RT-qPCR for regions –408 and –340 of the *Tnf* and *Axin2* genes, respectively, following incubation with anti–β-catenin antibody, and the data reported as fold enrichment over IgG background. (**D**) TNF-α ELISA using supernatants of *Lyz2-Cre^+/+^* *YFP^+/–^* *Ctnnb1*^Ex3Δ/wt^ or *Lyz2-Cre^+/+^* *YFP^+/–^*–derived NLDMs treated with or without LPS for 24 hours. (**E**) TNF-α ELISA of *Lyz2-Cre^+/+^* *YFP^+/–^* –derived NLDMs ± 10 mM LiCl or 100 ng/mL recombinant Wnt3a. (**F**) Treatment schedule for intranasal NLDM adoptive transfer in WT hosts injected with E0771.ML-1 cells via the tail vein 36 hours after treatment. (**G**) Bioluminescence images and quantification of data at endpoint, representative of 4 individual mice. In all data panels the mean ± SEM values are shown and represent 5 (**A** and **B**) or 4 (**G**) individual mice, technical quadruplicate of 3 pooled biological samples (**C**), or biological triplicates (**D** and **E**). Statistical analysis is based on 2-tailed *t* tests (**A**–**C** and **G**), 2-way ANOVA (**D** and **E**); * = *P* < 0.05.

In addition to being transcriptionally and phenotypically AM like, NLDMs derived from *Lyz2-Cre*^+/+^ *YFP^+/–^* and *Lyz2-Cre*^+/+^ *YFP^+/–^* *Ctnnb1*^Ex3*Δ*/wt^ mice faithfully reproduced the β-catenin–dependent mRNA expression findings observed in vivo. NLDMs derived from the *Lyz2-Cre*^+/+^ *YFP^+/–^* *Ctnnb1*^Ex3*Δ*/wt^ model maintained a robust increase in *Axin2* and *Ccnd1* expression, classical readouts of Wnt/β-catenin signaling (Figure 5A). Furthermore, both NLDM genotypes maintained the same differential expression pattern in 8 of the top 10 DEGs identified by the RNA-Seq experiment of in vivo AMs shown in Figure 4, including *Tnf* (Figure 5B). To demonstrate a physical interaction between β-catenin and the *Tnf* gene regulatory region, we performed a cleavage under targets and release using nuclease (CUT&RUN) assay of untreated *Lyz2-Cre*^+/+^ *YFP^+/–^* control NLDMs guided by anti–β-catenin or isotype-matched IgG antibodies. We observed amplification of DNA fragments from the *Tnf* and *Axin2* upstream regulatory regions in anti–β-catenin–treated samples. Signal was elevated above control IgG for both regions, while there was no difference in fold enrichment between *Tnf* and the *Axin2* positive control (Figure 5C) (53). Moreover, constitutively active β-catenin in NLDMs from the *Lyz2-Cre*^+/+^ *YFP^+/–^* *Ctnnb1*^Ex3Δ/wt^ model increased TNF-α protein secretion in response to the TLR4 agonist LPS (Figure 5D). NLDMs of *Lyz2-Cre*^+/+^ *YFP^+/–^* control mice pharmacologically treated with the Wnt/β-catenin degradation complex inhibitor, LiCl, or a Wnt agonist, Wnt3a, maintained enhanced TNF-α protein secretion following LPS treatment, consistent with the *Lyz2-Cre*^+/+^ *YFP^+/–^* *Ctnnb1*^Ex3*Δ*/wt^ model (Figure 5E). Taken together, these data demonstrate that NLDMs are an accurate in vitro model of AMs. Through interrogation of the NLDM model, we demonstrated a direct interaction between β-catenin and the *Tnf* regulatory domain, and that activation, both genetically and pharmacologically, enhanced TNF-α secretion.

### Localized adoptive transfer of β-catenin constitutively active NLDMs increases experimental metastatic burden.

To validate the specific effect of β-catenin activation as an enhancer of experimental metastasis, we made use of an AM adoptive transfer model. The lungs of syngeneic WT C57BL/6 mice were preseeded intranasally with NLDMs derived from either *Lyz2-Cre*^+/+^ *YFP^+/–^* control or *Lyz2-Cre*^+/+^ *YFP^+/–^* *Ctnnb1*^Ex3*Δ*/wt^ mice 36 hours prior to E0771.ML-1 tail vein injection. After 1 week, NLDMs were replenished by an additional intranasal adoptive transfer before experimental endpoint, 14 days after tumor implantation (Figure 5F). The persistence of intranasally transferred control NLDMs was tracked for *Lyz2*-YFP expression by flow cytometry (Supplemental Figure 5, E and F). At endpoint, bioluminescence imaging revealed increased experimental metastasis in WT mice receiving NLDMs of *Lyz2-Cre*^+/+^ *YFP^+/–^* *Ctnnb1*^Ex3*Δ*/wt^ mice (Figure 5G). Within the lung environment, there was no significant difference between the AM and resident monocyte populations. However, there was a marked increase in the proportions of interstitial macrophages and inflammatory monocytes, suggesting increased recruitment of myeloid cells (Supplemental Figure 5G). In the E0771.ML-1 model, PMN infiltration remained unchanged relative to the controls (Supplemental Figure 5H).

To further expand upon these findings, we tested the effects of NLDM adoptive transfer in B16F10 experimental metastasis following the same intranasal adoptive transfer protocol. Concurrent with our E0771.ML-1 experiments, NLDMs of *Lyz2-Cre*^+/+^ *YFP^+/–^* *Ctnnb1*^Ex3*Δ*/wt^ mice administered intranasally increased tumor burden relative to the controls, as measured by expression of the melanocyte-specific transcripts *Trp2* and *Pmel* (Supplemental Figure 6A). Surprisingly, in the B16F10 model, there was no significant difference in the inflammatory monocyte or interstitial macrophage compartments (Supplemental Figure 6B). However, there was a significant increase in PMNs within the lung environment (Supplemental Figure 6C). Therefore, myeloid infiltration driven by β-catenin constitutively active NLDMs is conserved between models, although some tumor-specific differences remain. Taken together, these findings demonstrate a causal role for β-catenin activation in AMs via an adoptive transfer model in enhancing experimental metastasis and myeloid immune cell infiltration into the lung.

### Local delivery of anti–TNF-α neutralizing antibody rescues the pro-metastatic effects of β-catenin activation in AMs, and TNF correlates with CTNNB1 expression in the AMs of cancer patients.

**Figure 6:**
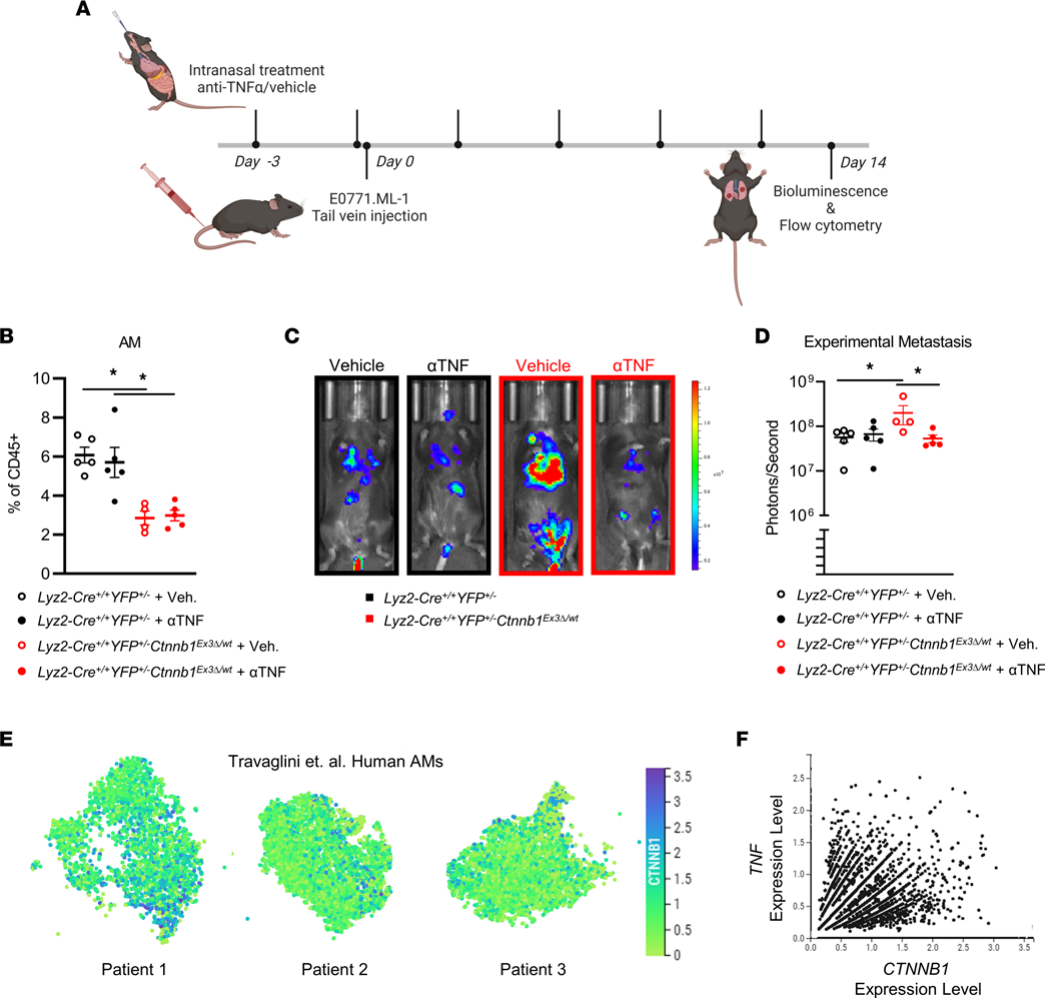
Intranasal delivery of anti–TNF-α neutralizing antibody rescues the pro-metastatic effects of AM β-catenin activation, and *TNF* expression correlates with *CTNNB1* in human AMs. (**A**) Schematic of intranasal anti–TNF-α treatments in *Lyz2-Cre^+/+^* *YFP^+/–^* *Ctnnb1*^Ex3Δ/wt^ or *Lyz2-Cre^+/+^* *YFP^+/–^* hosts injected with E0771.ML-1 via tail vein 36 hours after treatment. (**B**) Relative abundance of CD45^+^Ly6G^–^CD24^–^CD64^+^CD11b^lo^CD11c^+^ AMs of E0771.ML-1 tumor–bearing mice described in **A**. (**C**) Bioluminescence images, representative of 2 independent experiments and (**D**) quantification of data at endpoint represented. (**E**) Expression of *CTNNB1* in human AMs from Travaglini et al. by scRNA-Seq (European Genome-phenome Archive [EGA], EGAS00001004344); *n* = 3. (**F**) Scatterplot of *TNF* and *CTNNB1* expression from **D**; *n* = 3, pooled; positive Pearson correlation *P* = 0.0470. (**B** and **D**) The mean ± SEM values are shown and represent 4–5 individual mice. Statistical analysis is based on 1-way ANOVA with the Holm-Šídák correction applied for multiple comparisons (**B** and **D**); * = *P* < 0.05.

Given that β-catenin activation in AMs upregulates *Tnf* expression and enhances metastasis concurrently, we sought to establish a causal link between TNF-α protein bioavailability and lung metastatic burden. Anti–TNF-α neutralizing antibody was administered intranasally to localize effects to the lung following a protocol similar to a previous neutralization study (54) but with the same dosing regimen as the AM depletion studies (Figure 6A). Consistent with our previous findings, there was a significant decrease in the number of AMs in *Lyz2-Cre*^+/+^ *YFP^+/–^* *Ctnnb1*^Ex3*Δ*/wt^ compared with the controls. However, between the treatment groups, the localized administration of anti–TNF-α neutralizing antibody did not alter either the proportion or abundance of AMs in the lung (Figure 6B and Supplemental Figure 6D). This suggests that the previously reported β-catenin–mediated cell cycle arrest of AMs is TNF-α independent. Importantly, anti–TNF-α neutralizing antibody treatment was effective in rescuing the pro-metastatic phenotype observed in the *Lyz2-Cre*^+/+^ *YFP^+/–^* *Ctnnb1*^Ex3*Δ*/wt^ model, demonstrating a causal role for TNF-α (Figure 6, C and D).

To begin to tease apart whether TNF-α affects disease outcome directly or indirectly, we returned to in vitro settings that evaluate migration or invasion capacity based on the rationale that these activities associate with pro-metastatic behavior. From our NLDM-derived AM model, our data indicated that supernatants collected from cells with enhanced β-catenin led to heightened tumor cell migration in vitro compared with the control group (Supplemental Figure 6E, left). Second, to reveal potential links to TNF-α, we examined the impact of TNF blockade in the migration assay and found that anti-TNF antibody reduced migration in cocultures of tumor cells with supernatants from NLDM-derived AMs with or without enhanced β-catenin expression (Supplemental Figure 6E, left). Interestingly, while anti-TNF antibody decreased migration using supernatants from NLDM-derived AMs expressing enhanced β-catenin, it did not reduce migration to the level seen with the control group. These findings suggest a potential role for additional β-catenin–dependent secreted factors in enhancing tumor cell migration. Supernatants collected from NLDM-derived AMs with enhanced β-catenin also led to enhanced tumor cell invasion in vitro compared with the control group (Supplemental Figure 6E, right). These findings support the possibility for direct interactions between AM products and tumor cells and provide a potential basis for increased metastasis via a TNF-dependent mechanism.

To translate our β-catenin/TNF-α axis findings to the human cancer setting, we investigated the expression of *TNF* and *CTNNB1* in human lung AMs at the single-cell level using scRNA-Seq data generated by Travaglini et al. (55). While previous studies of macrophages in the lung have focused on resected tumor tissue, which is primarily infiltrated by bone marrow monocyte-derived TAMs at the expense of tissue-resident AMs, the samples the Travaglini group studied originated from uninvolved regions of the lung. First, we verified that *CTNNB1* is expressed in human AMs (Figure 6E). Next, we compared *CTNNB1* and *TNF* expression on a per-cell basis, unveiling a positive correlation between expression of the 2 genes within individual AMs pooled from the 3 patients (Figure 6F).

In summary, β-catenin activation in AMs enhanced metastasis in spontaneous and experimental murine models via a TNF-α–dependent mechanism. Furthermore, localized treatment with anti–TNF-α neutralizing antibody rescued the β-catenin–activated AM pro-metastatic phenotype, and this β-catenin/TNF-α axis was observed in the AMs of patients with lung cancer . Thus, in settings wherein TNF-α may exert pro- rather than antitumor effects, TNF-α may serve as a potential target in ameliorating lung metastasis or colonization via the local delivery of existing anti–TNF-α biologics.

## Discussion

Metastasis is the leading cause of mortality in multiple solid malignancies, including breast cancer and melanoma (1). Metastasis is guided not only by tumor-intrinsic features but also by the complex interactions between tumor cells and the stromal or resident cells of the primary site and distal organs in which they colonize (10). Macrophages infiltrate or preexist as tissue-resident populations in nearly every tissue of the body and are essential in maintaining tissue homeostasis (8). In cancer, both BMDMs and TRMs can aid metastasis (56, 57); however, the mechanisms that govern TRM behavior remain less well understood. Our study demonstrated that Wnt/β-catenin signaling in AMs of the metastatic lung environment promotes a TNF-α–dependent pro-metastatic program in 3 preclinical metastatic models.

Wnt/β-catenin signaling is a known hallmark of cancer (36). Given the abundance of intratumor macrophages in multiple solid cancers (8), including breast cancer, the pro-tumorigenic effects of macrophage-derived Wnt7b secretion in breast cancer (27), and a recent study of decreased primary lung tumor growth following β-catenin knockout in total F4/80^+^ macrophages (29), we tested the hypothesis that heightened β-catenin signaling in macrophages, particularly TRMs of the lung, enhanced metastatic outcome. Using a genetic gain-of-function approach, i.e., *Lyz2-Cre*^+/+^ *Ctnnb1*^Ex3*Δ/Ex3*Δ^ (βcat.CA) mice, initially in a preclinical model of TNBC, we observed that both primary tumor growth and metastasis to the lung were markedly enhanced relative to the floxed controls. We strengthened the importance of this signaling axis in breast cancer biology by revealing that Wnt signaling is a top-ranked pathway among those most associated with poor overall survival in a 1,006-patient breast cancer cohort with matched mRNA expression and survival data from TCGA. In addition, higher expression of the canonical Wnt ligand Wnt3a correlated with worse outcomes within the same data set.

The *Lyz2*-driven promoter used in these conditional β-catenin gain-of-function studies is known to be broadly expressed by mature myeloid cells and has been reported to be active in PMNs, monocytes, macrophages, and DCs (38). Therefore, to clarify which myeloid population was most affected using this model, we crossed this conditional β-catenin gain-of-function model to a YFP Cre-reporter strain. In doing so, we identified that AMs were the myeloid cell type with the highest penetrance of *Lyz2*-Cre activity in E0771.ML-1 tumor–bearing mice. This observation further strengthened our rationale for focusing on AMs in this study. As expected, there was considerable reporter gene expression across multiple myeloid cell subsets as well. However, PMNs within the primary tumor had relatively low *Lyz2*-Cre penetrance relative to PMNs within the lung and still less compared with splenic and peripheral blood PMNs from healthy mice as reported in the literature (58). Nevertheless, primary growth and lung metastasis were increased in *Lyz2-Cre*^+/+^ *Ctnnb1*^Ex3*Δ*/wt^ *ROSA26-EYFP^+/–^* mice relative to controls, consistent with the *Lyz2-Cre*^+/+^ *Ctnnb1*^Ex3*Δ/Ex3*Δ^ homozygous model.

We extended our findings to 2 additional preclinical metastatic models, B16F10 melanoma and LLC. In all 3 tumor models, E0771.ML-1, B16F10, and LLC, we observed increased spontaneous metastasis. While there was no effect on the abundance of lymphocytes in the lung, there was an increase in PMN infiltration. Increased PMN or PMN-MDSC infiltration into the lung has been associated with worse outcomes in multiple preclinical tumor models (59–62). Intriguingly, we observed a decrease in AM abundance; however, a recent study using the same βcat.CA model demonstrated that constitutively active β-catenin causes an arrest during the G0 to S phase transition in AMs, thereby suppressing self-renewal (30).

Since primary tumor burden is known to correlate with increased metastasis and myeloid cell abundance in the periphery, we utilized experimental metastasis models to further dissect the importance of heightened myeloid β-catenin expression in aiding increased tumor metastasis, without the confounding variable of the primary and/or differential primary tumor growth rates (63). Importantly, we found that near-complete depletion of AMs negated the difference in experimental metastasis between the myeloid β-catenin transgenic and control mice. AM depletion alone in the control mice was sufficient to significantly reduce metastasis, in line with previously reported AM depletion studies in the 4T1 metastatic mammary tumor model (47). Although the macrophage depletion experiments established a causal role for AMs in promoting their metastatic effect, to better understand the potential molecular events or mechanisms guiding that pro-metastatic phenotype, we turned to transcriptomic analyses.

Interestingly, despite increased lung metastasis in tumor-bearing mice, the transcriptional program upregulated in AMs with constitutively active β-catenin comprised a mix of pro- and antiinflammatory factors. While pathways with strong association to metastasis, such as epithelial-mesenchymal transition (EMT), complement, regulation of cell adhesion, tissue remodeling, and vascular development, were upregulated as expected, there was a surprising increase in inflammatory and chemotactic pathways as well. In reference to the latter, a significant upregulation of the pro-tumorigenic cytokines *Ccl3*, *Ccl9*, *Cxcl1*, and *Cxcl2* was observed (Supplemental Table 1), all known to play crucial roles in recruiting immune-suppressive cells, including PMNs, to the TME (64–67). These findings support our observation of increased PMN infiltration into the lungs of tumor-bearing mice, suggesting a potential cellular network between AMs and PMNs in which β-catenin activity in AMs triggers migration of PMNs to the lung via chemotactic signals.

Historically, the M1 and M2 classification in macrophage-tumor biology has been used to describe antitumor or pro-tumor functional states, respectively (68). Indeed, previous studies in the field that focused on in vitro*–*derived BMDMs and primary tumor TAMs, which are mainly bone marrow monocyte derived, have suggested that β-catenin signaling induces an M2-like expression profile (69, 70). Here, we demonstrated that while β-catenin–activated AMs are pro-tumorigenic and, therefore, seemingly M2-like in that regard, they expressed substantially higher levels of *Tnf*, a widely reported M1-type phenotypic marker (71). Thus, in the case of AMs and NLDMs in our study, β-catenin induced a mixed, but pro-tumorigenic, M1/M2 TRM-type phenotype. Given that β-catenin interacts with a wide variety of cofactors in the nucleus, further experiments are required to delineate the precise complexity of interactions that define the context-dependent effects of β-catenin in BMDMs and TRMs (72, 73). It is important to point out that future comprehensive studies are warranted to translate our findings to human breast cancer and melanoma that has metastasized to the lung. However, a study by Sarode et al. (29) lends support to the notion that AMs do express enhanced β-catenin in the context of primary lung cancer. Moreover, Sarode et al. (29) found that enhanced β-catenin expression in human macrophages was associated with an M2-like phenotype. Thus, these findings support the hypothesis for a β-catenin/AM/metastasis axis in breast cancer or melanoma biology. Comprehensive histopathologic analyses on such tissue biospecimens may be one approach to strengthen human relevance, especially assessing whether AMs spatially localize near/around the metastatic sites.

TNF-α itself is a complex cytokine that has been the center of intense research focus in cancer medicine since its discovery by Carswell and colleagues in 1975 (74). While named for its antitumor properties at high doses in sarcoma, TNF-α has been shown to promote tumor growth under certain circumstances through the recruitment of suppressive myeloid cells to the TME, activation-induced cell death of cytotoxic T cells, and the induction of EMT in tumor cells themselves (75–77). Conversely, another study demonstrated that systemic TNF-α blockade can both reduce the toxicities of and improve the response to immunotherapy in melanoma (78), suggesting that tumor response to TNF-α is tumor intrinsic and/or context dependent. Here, our findings support a potential pro-metastatic role for TNF-α and, furthermore, reveal that localized delivery of TNF-α neutralizing antibodies can overcome the β-catenin–TNF-α–mediated pro-metastatic AM response. However, the precise interactions between AM-secreted TNF-α and the stroma or the tumor cells themselves remain a complex network that warrants further detailed investigation. While our data support a significant role for TNF-α, it is reasonable to hypothesize that these other chemokines/cytokines identified in our analyses play relevant roles in metastasis through the recruitment of pro-tumorigenic myeloid or lymphoid populations or the suppression of immune effector cells. One potential explanation for why anti–TNF-α antibody alone was able to significantly inhibit metastasis, however, is that TNF-α may be a central mediator in the context of β-catenin regulation of AM-mediated metastasis in vivo. Thus, in that context, reducing TNF-α levels may in turn dampen the production of additional (perhaps, downstream) inflammatory cytokines/chemokines produced by AMs or other stromal cell types of the lung TME. This notion is synonymous with how single-agent biologics, such as TNF-α antagonists, are thought to ameliorate the progression of autoimmune disorders in vivo (e.g., rheumatoid arthritis or Crohn’s disease) characterized by complex inflammatory networks.

It also remains unclear whether TNF-α affects lung metastasis directly or indirectly. To that end, using in vitro coculture systems, our data support the possibility for direct interactions between AM products and tumor cells. We showed that supernatants collected from AMs with enhanced β-catenin expression led to heightened tumor cell migration and invasion, outcomes characteristic of pro-metastatic behavior, and that anti–TNF-α antibody reduced the extent of migration. However, it is important to note that TNF-α blockade did not reduce the extent of tumor cell migration with supernatants from AMs with enhanced β-catenin to the same level as the control supernatants. These data suggest that other cytokines/chemokines in this in vitro context may be at play for these activities, which is consistent with our RNA-Seq data revealing multiple cytokine/chemokine differences. Future work, however, is warranted to explore these interactions in vivo, as well as to investigate the possibility for indirect interactions.

In summary, we demonstrated that 1) β-catenin activation in AMs heightened a pro-metastatic shift in the lung metastatic microenvironment; 2) AMs with enhanced β-catenin activity expressed an inflammatory metastatic program defined by multiple pathways, including increases in PMN chemoattractants and *Tnf* signaling; and 3) this metastatic phenotype was ameliorated by localized TNF-α blockade. Together these findings highlight a potentially novel application for existing anti–TNF-α biologics in suppressing metastasis to the lung in settings wherein tumors are resistant or refractory to the antitumor actions of this complex cytokine.

## Methods

### RNA-Seq data.

RNA-Seq data original to this paper and shown in Figure 4, Supplemental Figure 4, and Supplemental Tables 1–3 were deposited to GEO under accession number GSE200508.

### Statistics.

Data were analyzed using GraphPad Prism (v9.1.2). Data represent mean ± SEM, with the number of mice or biologic or experimental replicates indicated. Differences between groups in primary tumor growth over time or TNF-α secretion were determined by a 2-way ANOVA. Differences in metastatic outcome or cell populations as assessed by flow cytometry between multiple treatment groups were determined by 1-way ANOVA with the Holm-Šídák correction applied for multiple comparisons in the specified figures. Differences in metastatic outcome, cell populations, or mRNA expression between only 2 treatment groups were determined by a 2-tailed unpaired *t* test. Statistical tests following RNA-Seq analyses were performed in R (v4.1.0) and are included in their respective figure legends. In all cases, *P* values less than 0.05 were considered statistically significant.

### Study approval.

All studies involving mice were approved under protocols 1108M and 1117M and conducted in accordance with the Institutional Animal Care and Use Committee at the Roswell Park Comprehensive Cancer Center.

Further information can be found in Supplemental Methods.

## Author contributions

EDK, SLT, and SIA supervised and designed the study. EDK, SHC, MLH, CMB, and SLT performed the experiments. BEC and MMT provided essential mouse models. EDK, SLT, and SIA analyzed the data. EDK and SIA verified statistical analyses. EDK, SHC, and SIA wrote the manuscript. SLT, CMB, BEC, and MMT provided crucial feedback and helped edit the manuscript.

## Supplementary Material

Supplemental data

## Version 1 04/24/2023

Electronic publication
