# Supplementary material for: β-Catenin signaling in alveolar macrophages enhances lung metastasis through a TNF-dependent mechanism
Source: JCI Insight. 2023 Apr 24;8(8):e160978. doi: 10.1172/jci.insight.160978 (PMC10243816; doi:10.1172/jci.insight.160978)
Supplement: Supplemental data [file jciinsight-8-160978-s041.pdf]

Supplemental Methods

Mice

All experiments were performed under protocols (1108M, 1117M) approved by the Institutional Animal Care and Use Committee (IACUC) of the Roswell Park Comprehensive Cancer Center. All experiments were performed using female or male mice 8-12 weeks of age, as specified in the next section. Male or female C57BL/6 mice were obtained from the Charles River Laboratories (Wilmington, MA). *Ctnnb1*$^{Ex3\Delta/Ex3\Delta}$ on a C57BL/6 background were kindly provided by Dr. Makoto Taketo (Kyoto University, Kyoto, Japan) (1) via Drs. Aimin Jiang (at Roswell Park, now at the Henry Ford Health System, Detroit, MI) and Björn E. Clausen University Medical Center of the Johannes Gutenberg-University, Mainz, Germany), and both *Lyz2*-cre$^{+/+}$ and *ROSA26-EYFP*$^{+/+}$ mice also on a C57BL/6 background were purchased from The Jackson Laboratory (Bar Harbor, ME) (2, 3). Genotyping of mouse strains was performed on DNA isolated from tail clips using the DirectPCR Lysis Reagent (Viagen Biotech) per the manufacturer's instructions. Specific primers for validating the different genotypes are found in Supplemental Table 4.

Cell Lines and Tumor Growth Experiments

The E0771.ML-1 mammary tumor cell line, a more aggressive variant of the parental E0771 cell line, was kindly provided by Dr. Vivek Mittal (Weill Medical College at Cornell University, New York, NY). Cell lines were confirmed to be mycoplasma-negative using the Mycoplasma PCR Primer Set (Agilent Technologies) and authenticated using the Mouse Cell STR Profiling Service provided by the ATCC. E0771.ML-1 cells were cultured in a RPMI-based

culture media (containing 10% heat-inactivated FBS, 20mM HEPES, 100 IU/mL Penicillin, and 100μg/mL Streptomycin). B16F10 were purchased from ATCC (Manassas, VA) and cultured in a DMEM-based culture media (containing 10% heat-inactivated FBS). Both lines were cultured at 37°C with 5% CO_2 and were removed from the flasks by trypsinization. The luciferase expressing LLC (LLC-luc) cell line was kindly provided by Dr. Pamela Hershberger (Roswell Park Comprehensive Cancer Center, Buffalo, NY). LLC-luc cells were maintained in DMEM-based culture media as with B16F10.

For the orthotopic E0771.ML-1 experiments, cells ($1x10^5$) were suspended in a Matrigel and DPBS mixture (at a 1:1 ratio) and were implanted into mammary gland no. 4 of syngeneic female C57BL/6 mice. For the orthotopic B16F10 experiments, cells ($3x10^5$) were suspended in DPBS and were implanted into the skin on the flank of syngeneic male C57BL/6 mice. LLC-luc cells ($5x10^5$) were implanted along the flank in a similar manner as B16F10.Tumor growth was measured 3 times per week, and the volumes were calculated using the formula $(w^2 \text{ x } l)/2$, where 'w' represents width and 'l' represents length. For the E0771.ML-1 experimental lung metastasis studies, cells ($1.25x10^5$) in DPBS were injected via the tail vein of syngeneic female C57BL/6 mice. For B16F10 and LLC-luc experimental metastasis studies, cells ($2x10^5$) in DPBS were injected via the tail vein of syngeneic male C57BL/6 mice, and experimental metastasis studies were terminated 14 days post injection. (See Supplemental Table 5 for a listing of all biologics, chemical, reagents, cell and animal lines.)

In Vivo Treatment Studies

Alveolar macrophage depletion studies were conducted by administering 60μL of clodronate liposomes (FormuMax) or DPBS as vehicle control under isoflurane anesthesia twice

prior to tail vein injection of E0771.ML-1 tumor cells, and then continued every 3 to 4 days over 14 days for a total of 6 treatments. Localized anti-TNF-α (BioXCell) therapy was performed following the same technique and schedule as above, utilizing 10μg doses of mAb in 50μL or volume-match DPBS.

NLDMs were generated from *Lyz2*-Cre$^{+/+}$*YFP*$^{+/-}$ control or *Lyz2*-Cre$^{+/+}$*YFP*$^{+/-}$ *Ctnnb1*$^{Ex3\Delta/wt}$ mice and harvested, as described in the section below on "In vitro Neonatal Liver-Derived Macrophage Differentiation". NLDMs ($2.5x10^5$) suspended in 50μL of DPBS were administered under isoflurane anesthesia 36 hours prior to tumor cell line implantation via tail vein injection and NLDM adoptive transfer was repeated 7 days post injection.

Analyses of the Primary or Metastatic Tumor Microenvironment

Tumor tissues and lungs were dissected after euthanasia (at the days post-implantation indicated in the specific figures or figure legends) and dissociated into a single-cell suspension using the gentleMACS automatic tissue dissociator system (Miltenyi Biotec) in a collagenase/hyaluronidase cocktail (Stem Cell Technologies) containing 30μg/mL DNase (Roche, Basel, Switzerland), as per the manufacturer's instructions. Dissociation occurred in a Hybaid rotating incubator (Phoenix Equipment) for 1 hour at 37°C. The resulting mixture was then strained through 100μm SureStrain cell strainers (MTC Bio). Neonatal livers were surgically removed from 1-day old neonates following euthanasia, and mechanically dissociated and passed through a 100μm SureStrain cell strainer. Bone marrow (BM) cells were collected by flushing tibias and femurs. For all tissues, RBCs were lysed using ammonium-chloride-potassium (ACK) lysis buffer, and the cells were then used for further analysis, as described in the appropriate assays.

For quantification of metastasis in the spontaneous lung metastasis setting, the lungs were collected following euthanasia, washed in DPBS, and fixed in 10% formalin. Slides were prepared from formalin-fixed paraffin-embedded blocks, followed by histologic staining with hematoxylin and eosin, and quantification of the metastatic nodules under light microscopy in a blinded manner. For quantification of lung metastasis in the experimental lung metastasis setting, the lungs were removed following euthanasia, washed in DPBS, and fixed in 10% formalin and quantified under a dissecting light microscope in a blinded manner. For quantification of experimental E0771.ML-1 lung metastasis by bioluminescence, a dose of 150mg/kg of D-Luciferin (Goldbio) was administered to each mouse under isoflurane anesthesia by intra-peritoneal injection 15 minutes prior to imaging by the IVIS Spectrum (Perkin-Elmerand acquired and analyzed via Living Image software (v4.7.3). In orthotopic tumor implantation experiments, mice were euthanized 15 minutes after D-luciferin administration and the lungs were surgically removed and imaged ex vivo.

In vitro Neonatal Liver-Derived Macrophage Differentiation

Single cell suspensions from neonatal livers (as described under the section on "Tissue Analysis") were plated at a density of $5x10^4$ cells/cm^2 in complete RPMI (containing 10% heat-inactivated FBS, 15mM HEPES, 2 mM L-glutamine, 0.1mM nonessential amino acids, 1 mM sodium pyruvate, 100 IU/mL Penicillin, 100μg/mL Streptomycin, and 50μM 2-metcaptoethanol) with 30ng/mL GM-CSF (Peprotech) at 37°C with 5% CO_2. Media was replaced at day 7, and the cells were recovered for subsequent analysis at day 14. To recover the cells, the media was removed and replaced with CellStripper (Corning) and incubated for 15 minutes at 37°C with

5% CO_2, followed by vigorous pipetting and a DPBS rinse step to collect the loosely adherent cells.

In vitro Bone Marrow-Derived Macrophage Differentiation

BM single cell suspension (as described under the section on "Tissue Analysis") were plated at a density of 7.5×10^4 cells/cm^2 in complete RPMI with 30ng/mL M-CSF (Peprotech) at 37°C with 5% CO_2 for 6 days.

CUT&RUN

NLDMs were derived from *Lyz2*-Cre$^{+/+}$*YFP*$^{+/-}$ mice (as described under the section on "In vitro Neonatal Liver-Derived Macrophage Differentiation"). CUT&RUN (Cell Signaling Technology) was performed on 1×10^5 suspended NLDM cells incubated with rabbit anti-mouse β-catenin or rabbit IgG isotype control overnight at 1:25 and 1:20 concentration, respectively, following the manufacturer's instructions. β-catenin binding to the *Tnf* and *Axin2* regulatory domains were determined by qPCR with the primers indicated in Supplemental Table 4 using the SYBR-Green (Invitrogen) reaction mix and CFX Maestro 1.1 (Bio-Rad) instrument. Quantification was measured as fold enrichment = $2^{(\text{Ct } \alpha\text{-}\beta\text{-catenin} - \text{Ct IgG})}$.

ELISA

NLDMs were derived and collected from the indicated genotypes (as described under the section on "In vitro Neonatal Liver-Derived Macrophage Differentiation"). NLDMs (1.5×10^5) were loaded into each well of a 96-well flat-bottomed plate in complete RMPI with 30ng/mL GM-CSF (Peprotech) at 37°C with 5% CO_2 and allowed to adhere for 3 days. Media was

removed and replaced with complete RPMI ± LPS (10, 100, or 1000ng/mL) and/or 10mM LiCl (Sigma), or 100ng/mL murine Wnt3a (Peprotech) as indicated in the figure legends. After 24 hours at 37°C with 5% CO_2, plates were centrifuged at 300g to pellet the cells and 100uL of supernatant was carefully collected. TNF-α ELISA was performed according to the manufacturer's instructions (Biolegend) using a 1:10 dilution of supernatant in buffer for each sample well. Signal was quantified using the Synergy 2 (Biotekand) analyzed via Gen5 software (v1.11).

TCGA Analysis

Survival analysis was performed using the publicly available webtool OncoLnc (http://www.oncolnc.org/) where upper and lower tertile cutoffs for Wnt3a mRNA expression were applied. Genes associated with poor overall survival in breast cancer were identified by Anaya utilizing a Cox regression model of data obtained from TCGA representing 1,006 breast cancer patients (4). A gene list containing the top 500 genes associated with poor overall survival, that is, those with the greatest positive Cox coefficient, were assessed for KEGG pathway enrichment using the DAVID Bioinformatics Resource (https://david.ncifcrf.gov/) (5, 6).

Flow Cytometry

Analysis of lung active β-catenin

Single cell suspension of lung cells ($1x10^6$) was isolated (as described under the section on "Tissue Analysis") and blocked with anti-mouse CD16/32 for 10 minutes and immunostained with BUV395 anti-mouse CD45 and Live/Dead - Blue for 25 minutes at 4°C in a flow-based

buffer (DPBS with 0.5% BSA and 2mM EDTA) and washed once with the same buffer. Samples were fixed and permeabilized with the FoxP3 Staining Buffer Set (Miltenyi Biotec) following the manufactures instructions. Samples were then split into flow-minus one (FMO) or stained with Ax488 anti-mouse non-phospho active β-catenin (ABC) (Cell Signaling Technology) for overnight incubation at 4°C. Samples were washed according to the staining buffer instructions and analyzed on an LSR II cytometer (BD Biosciences, Mississauga, ON) running FACSDiva (v6.1.3). Data files were analyzed using FCS Express (v7.12).

Analysis of the lung microenvironment

Lung cells ($1x10^6$) were isolated (as described in the section on "Tissue Analysis") and blocked with anti-mouse CD16/32 for 10 minutes and immunostained with mAbs indicated in Supplemental Table 5 for 25 minutes at 4°C in the flow-based buffer and washed once with the same buffer. Samples were then resuspended in the flow-based buffer containing 1.5µM DAPI (Thermofisher), run on an LSR II cytometer and the data analyzed as described above. Data are reported as either the percentage of $CD45^+$ cells or cells/g of lung were indicated and calculated as *Lung Weight (g)/((% Live Cells/100) x Cell Count)*.

Analysis of the tumor microenvironment

Tumor cells ($1x10^6$) were isolated (as described under the section on "Tissue Analysis") and blocked with anti- mouse CD16/32 for 10 minutes and immunostained with mAbs indicated in Supplemental Table 5 for 25 minutes at 4°C in the flow-based buffer and washed once with the same buffer. Samples were then resuspended in the flow-based buffer containing 1.5µM DAPI (Thermofisher), run on an LSR II cytometer and the data analyzed as described above.

Fluorescence-Activated Cell Sorting

Lung cells ($1x10^6$) were isolated (as described in the section on "Tissue Analysis") and blocked with anti-mouse CD16/32 for 10 minutes and immunostained with mAbs indicated in Supplemental Table 5 for 25 minutes at 4°C in the flow-based buffer and washed once with the same buffer. Samples were then resuspended in flow-based buffer containing 1.5µM DAPI (Thermofisher) and the cells sorted using the MA900 Multi-Application Cell Sorter (SONY). Approximately 10^5 cells from the "AM+" gate were isolated per sample for RNA isolation and sequencing. Data files were analyzed using FCS Express (v7.12).

Invasion and Migration Assays

Migration and invasion assays were performed in Transwell migration chambers using a 24-well format (Corning Falcon) tested in triplicate determinations. For migration assays, $2x10^5$ E0771.ML-1 cells were carefully seeded onto the upper membrane in 200uL of serum-free RPMI (SFM) and allowed to settle for 10 minutes at 37°C. In the invasion assay, a 1:1 mix of 50µL Matrigel and $1x10^5$cells suspended in SFM was seeded, set for 30 minutes at 37°C, and coated in 100uL of SFM. Then, 600µL of 24-hr supernatant from *Lyz2*-Cre$^{+/+}$*YFP*$^{+/-}$ control or *Lyz2*-Cre$^{+/+}$*YFP*$^{+/-}$*Ctnnb1*$^{Ex3\Delta/wt}$ NLDMs was then added to the lower chamber. Anti-TNF-α neutralizing antibody was added to indicated wells at a final lower chamber concentration of 50µg/mL. Cultures were incubated for either 4- or 18-hrs to assess for migration or invasion capacity, respectively. Cells were fixed in methanol and stained with crystal violet as before counting.

RT q-PCR

Total RNA was isolated using the RNeasy Mini Kit (Qiagen). cDNA was synthesized using the iScript cDNA synthesis kit (Bio-Rad). The cDNA was used for PCR amplification using specific primer sets (Supplemental Table 4). qRT-PCR was performed using CFX Maestro 1.1 (Bio-Rad). SYBR-Green (Invitrogen) was used as the dye for quantification. Data were quantified using the formula-fold change = $2^{-\Delta\Delta Ct}$. All results were reported as a ratio of the specific mRNA signal normalized to the housekeeping gene.

RNA Sequencing Studies

RNA-seq

The purification of total and small RNAs were prepared using the miRNeasy mini kit (Qiagen) and immediately lysed and resuspended with the addition of 700 ul of QIAzol Lysis Reagent. The lysate was then left at room temperature for 15 minutes to allow for adequate cell lysis. After addition of chloroform, the homogenate was then separated into aqueous and organic phases by centrifugation. RNA partitions to the upper, aqueous phase, while DNA partitions to the interphase and proteins to the lower, organic phase or the interphase. The upper, aqueous phase was extracted, and ethanol added to provide appropriate binding conditions for all RNA molecules from 18 nucleotides upwards. The sample was then applied to the RNeasy Mini spin column, where the total RNA bound to the membrane and phenol and other contaminants were efficiently washed away. On-column DNAse digestion was also performed to remove any residual genomic DNA contamination followed by additional washes. High quality RNA is then eluted in 60 ul of RNase-free water. Quantitative assessment of the purified total RNA was

performed by Qubit Broad Range RNA kit (Thermofisher). The RNA was further evaluated qualitatively by a 4200 Tapestation (Agilent Technologies).

Illumina compatible NGS libraries were generated using the SMARTer Stranded v2 Ultra Pico Input Total RNA Seq kit (Clontech). 5 ng of total RNA was fragmented based on %DV200 analysis and RIN value and used to synthesize first-strand cDNA utilizing proprietary template switching oligos. Amplified ds cDNA was created by LD PCR using blocked PCR primers and unique sample barcodes were incorporated. The resulting ds cDNA was purified using AmpureXP beads (Beckman Coulter). Ribosomal cDNA was then depleted using R probes, and 12 cycles of PCR using universal PCR primers to complete the library. The final libraries were purified using AmpureXP beads and validated for appropriate size on a 4200 TapeStation D1000 Screentape (Agilent Technologies). The DNA libraries were quantitated using KAPA Biosystems qPCR kit, and pooled together in an equimolar fashion, following experimental design criteria. Each pool was denatured and diluted to 300pM with 1% PhiX control library added. The resulting pool was then loaded into the appropriate NovaSeq Reagent cartridge, as determined by the number of sequencing cycles desired, and sequenced on a NovaSeq6000 following the manufacturer's recommended protocol (Illumina Inc)

RNA-seq analysis

Six paired-end biological samples were obtained. A total of approximately 60 million reads were reported for each sample. First pass base pairs quality control (QC) was performed using Fastqc (v0.10.1). Spliced alignment was done using Bowtie (v1.0.1) and TopHat (v2.0.13), allowing a maximum of 1 mismatch per read. Alignment and downstream analyses were performed using GRCm38 (vM10) GENCODE reference and annotation of the mouse genome.

Separate lane replicates were merged into a single sample alignment file using MergeSamFiles from Picard (v1.97). Read counts were estimated with HTSeq (v0.9.1) using intersection-strict option. Differential expression analyses were performed using DESeq2 (v1.32.0). Downstream and visualization plots are done using regularized log2 transformation implemented by DESeq2. Heatmaps were generated using the R (v4.1.0) library pheatmap (v1.0.12). Gene Ontology clusters were determined by uploading all significantly upregulated genes (padj < 0.05) in Triple AMs in a gene list to Metascape (https://metascape.org/) with kappa set to 0.3 (7). All data are deposited under accession no. GSE200508.

scRNA-seq data acquisition and analysis

Data sets from Zhu et al. and Travaglini et al. were obtained from GSE164793 and EGAS00001004344, respectively (8, 9). Cellranger 10X h5 files were loaded into R (v4.1.0) using the Hdf5r (v1.3.5) package and analyzed using Seurat (v4.1.0) (10, 11). For Zhu et al. data, “ctr” and “wnt3a” data sets were combined using the Merge function. *Tnf* expression was assessed via the Featureplot and VlnPlot functions. For Travaglini et al data, *CTNNB1* feature plot and *CTNNB1* vs *TNF* scatter plots were generated with the CellxGene webtool (https://cellxgene.cziscience.com/) from the “krasnow_lab_human_lung_cell_atlas_10x” dataset. Lung macrophages were selected by expression of the top 10 reported “Cluster 47” markers reported by Travaglini et al. via the Moduelscore function on Merged $CD45^{+}$ lung 10X scRNAseq “P1_3”, “P2_2” and P3_7” h5 data files. Pearson correlation between *CTNNB1* and *TNF* was determined by the FeatureScatter function.

References

1. Harada N, Tamai Y, Ishikawa T, Sauer B, Takaku K, Oshima M, et al. Intestinal polyposis in mice with a dominant stable mutation of the beta-catenin gene. *EMBO J*. 1999;18(21):5931-5942.
2. Clausen BE, Burkhardt C, Reith W, Renkawitz R, Forster I. Conditional gene targeting in macrophages and granulocytes using LysMcre mice. *Transgenic Res*. 1999;8(4):265-277.
3. Srinivas S, Watanabe T, Lin CS, William CM, Tanabe Y, Jessell TM, et al. Cre reporter strains produced by targeted insertion of EYFP and ECFP into the ROSA26 locus. *BMC Dev Biol*. 2001;1:4.
4. Anaya J. OncoLnc: linking TCGA survival data to mRNAs, miRNAs, and lncRNAs. *Peerj Computer Science*. 2016.
5. Huang da W, Sherman BT, Lempicki RA. Systematic and integrative analysis of large gene lists using DAVID bioinformatics resources. *Nat Protoc*. 2009;4(1):44-57.
6. Huang da W, Sherman BT, Lempicki RA. Bioinformatics enrichment tools: paths toward the comprehensive functional analysis of large gene lists. *Nucleic Acids Res*. 2009;37(1):1-13.
7. Zhou Y, Zhou B, Pache L, Chang M, Khodabakhshi AH, Tanaseichuk O, et al. Metascape provides a biologist-oriented resource for the analysis of systems-level datasets. *Nat Commun*. 2019;10(1):1523.
8. Zhu BB, Wu Y, Huang S, Zhang RX, Son YM, Li CF, et al. Uncoupling of macrophage inflammation from self-renewal modulates host recovery from respiratory viral infection. *Immunity*. 2021;54(6):1200-+
9. Travaglini KJ, Nabhan AN, Penland L, Sinha R, Gillich A, Sit RV, et al. A molecular cell atlas of the human lung from single-cell RNA sequencing. *Nature*. 2020;587(7835):619-625.
10. Satija R, Farrell JA, Gennert D, Schier AF, Regev A. Spatial reconstruction of single-cell gene expression data. *Nat Biotechnol*. 2015;33(5):495-502.
11. Stuart T, Butler A, Hoffman P, Hafemeister C, Papalexi E, Mauck WM, 3rd, et al. Comprehensive Integration of Single-Cell Data. *Cell*. 2019;177(7):1888-902 e21.

Supplemental Figure 1

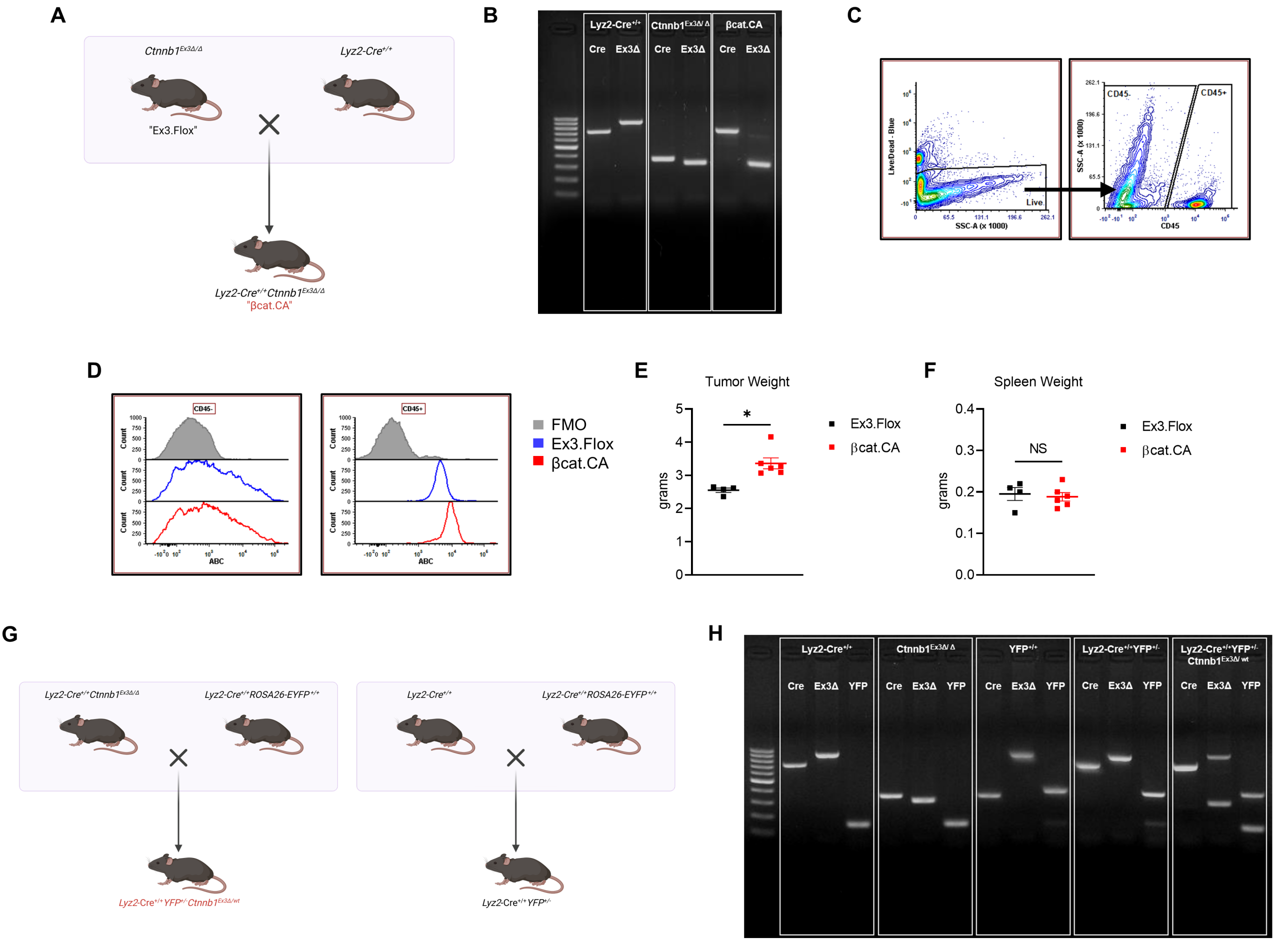

Supplemental Figure 1. Validation of Lyz2-cre$^{+/+}$Ctnnb1$^{Ex3\Delta/\ Ex3\Delta}$ model. (A) Schematic of the C57BL/6 mouse breeding strategy for *Ctnnb1*$^{Ex3\Delta/\Delta}$ (Ex3.Flox) and *Lyz2*-cre$^{+/+}$*Ctnnb1*$^{Ex3\Delta/\Delta}$ (βcat.CA) mice. **(B)** *Lyz2*-cre$^{+/+}$, *Ctnnb1*$^{Ex3\Delta/\Delta}$*,* and *Lyz2*-cre$^{+/+}$ *Ctnnb1*$^{Ex3\Delta/\Delta}$ (βcat.CA) genotyping of mice described in *A,* representative of three independent experiments. **(C)** Flow cytometry gating strategy for CD45^{-} stroma and CD45^{+} leukocytes after fixable live/dead staining of dissociated non-tumor bearing lung. **(D)** Non-phosphorylated active β-catenin (ABC) intracellular staining of Ex3.Flox and βcat.CA lungs, as gated in *C*. **(E)** Tumor and **(F)** spleen weights from E0771.ML-1-bearing mice at day 27 post-implantation as described in *Figure 1C*; statistical analysis is based 2-tailed *t*-test*.* **(G)** Schematic of C57BL/6 mouse breeding strategy for *Lyz2-Cre*$^{+/+}$*YFP*$^{+/-}$*Ctnnb1*$^{Ex3\Delta/wt}$ *and Lyz2-Cre*$^{+/+}$*YFP*$^{+/-}$ C57BL/6 mice. **(H)** genotyping of mice from *G* representative of three independent experiments. Data are recorded as the mean ± SEM of the indicated data points; * = $p < 0.05$, NS = not significant.

Supplemental Figure 2

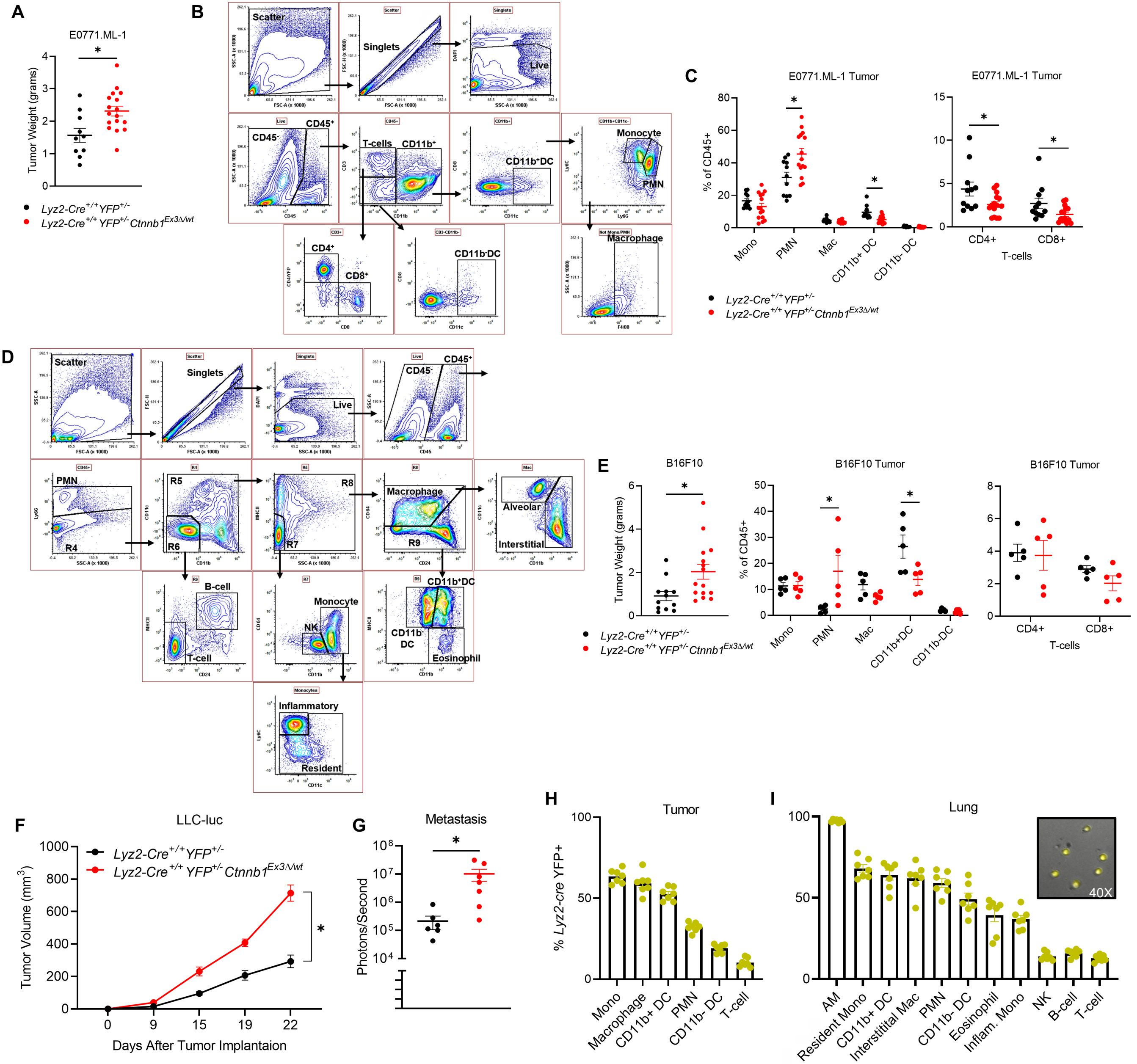

Supplemental Figure 2. Lyz2-cre$^{+/+}$Ctnnb1$^{Ex3\Delta/wt}$ROSA26-EYFP$^{+/-}$ validation and TME characterization. (A) Primary E0771.ML-1 tumor weights from *Figure 2A*. **(B)** Flow cytometry gating strategy for dissociated primary tumor tissues. **(C)** Myeloid, *left*, and lymphoid, *right,* components from the TME of *Figure S2A*, gated as in *S*; n = 10-17; 2-tailed *t*-test. **(D)** Flow cytometry gating strategy for dissociated lung tissue, as adapted from Yu et al. **(E)** *Left,* primary tumor weights from the TME of *Figure 2E*; myeloid, *middle,* and lymphoid, *right,* components from *Figure 2E*; n = 5-15; 2-tailed *t*-test. **(F)** Primary tumor growth of implanted LLC-luc tumor cells in *Lyz2-Cre*$^{+/+}$*YFP*$^{+/-}$*Ctnnb1*$^{Ex3\Delta/wt}$ and *Lyz2-Cre*$^{+/+}$*YFP*$^{+/-}$ C57BL/6 mice; n = 6-7; statistical analysis based on 2-way ANOVA. **(G)** Bioluminescence of ex vivo-imaged lungs in *E*; 2-tailed *t*-test. **(H)** Percentage of cell populations from dissociated tumor or **(I)** lung tissues positive for *Lyz2*-YFP expression in E0771.ML-1-bearing *Lyz2-Cre*$^{+/+}$*YFP*$^{+/-}$ mice as gated in *Figure S2B* or *S2D*, respectively. Inset reflects bronchioalveolar lavage (BAL) AMs at 40X original magnification, representative of 7 individual mice; n = 7, 2-tailed *t*-test. Data are recorded as the mean ± SEM of the indicated data points; * = $p < 0.05$, NS = not significant.

Supplemental Figure 3

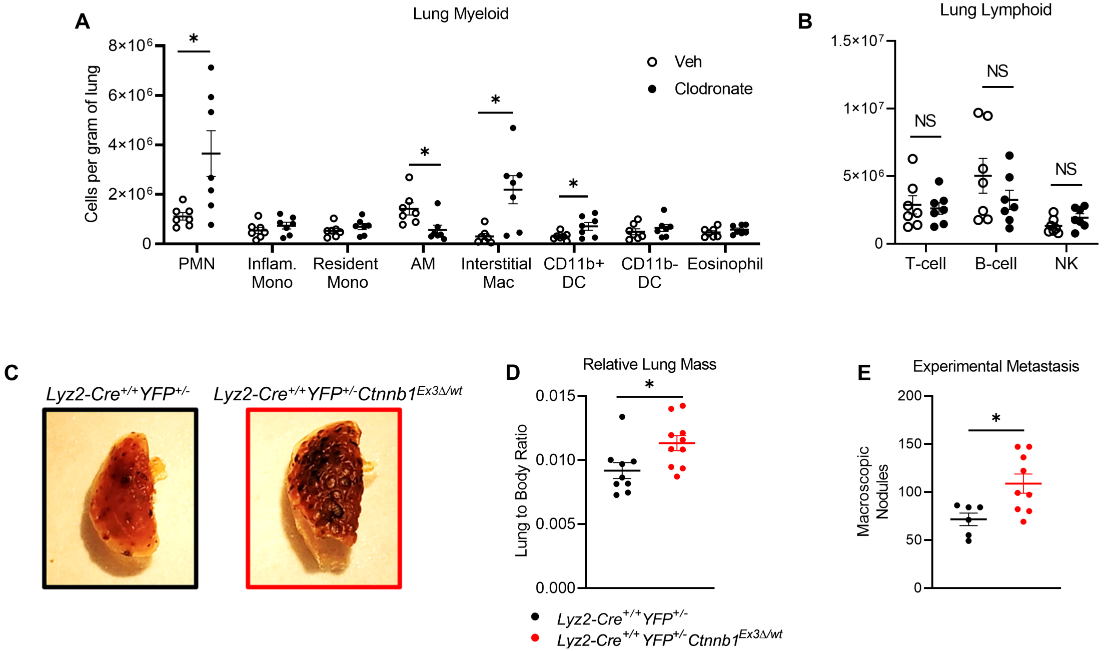

Supplemental Figure 3. Clodronate liposomes specifically deplete AMs. (A) Myeloid and **(B)** lymphoid lung environments of non-tumor bearing *Lyz2-Cre*$^{+/+}$*YFP*$^{+/-}$ mice following the clodronate liposome or vehicle control pre-depletion schedule from *Figure 3A*; n = 7; 2-tailed *t*-test. **(C)** Representative images of lungs from *Lyz2-Cre*$^{+/+}$*YFP*$^{+/-}$*Ctnnb1*$^{Ex3\Delta/wt}$ and *Lyz2-Cre*$^{+/+}$*YFP*$^{+/-}$ mice at endpoint 14 days post-tail vein injection of B16F10 cells, representative of two independent experiments. **(D)** Macroscopic lung nodule counts and **(E)** lung mass corrected for total body weight at endpoint represented in *C*; n = 6-9, 2-tailed *t*-test. Data are recorded as the mean ± SEM of the indicated data points; * = $p < 0.05$, NS = not significant.

Supplemental Figure 4

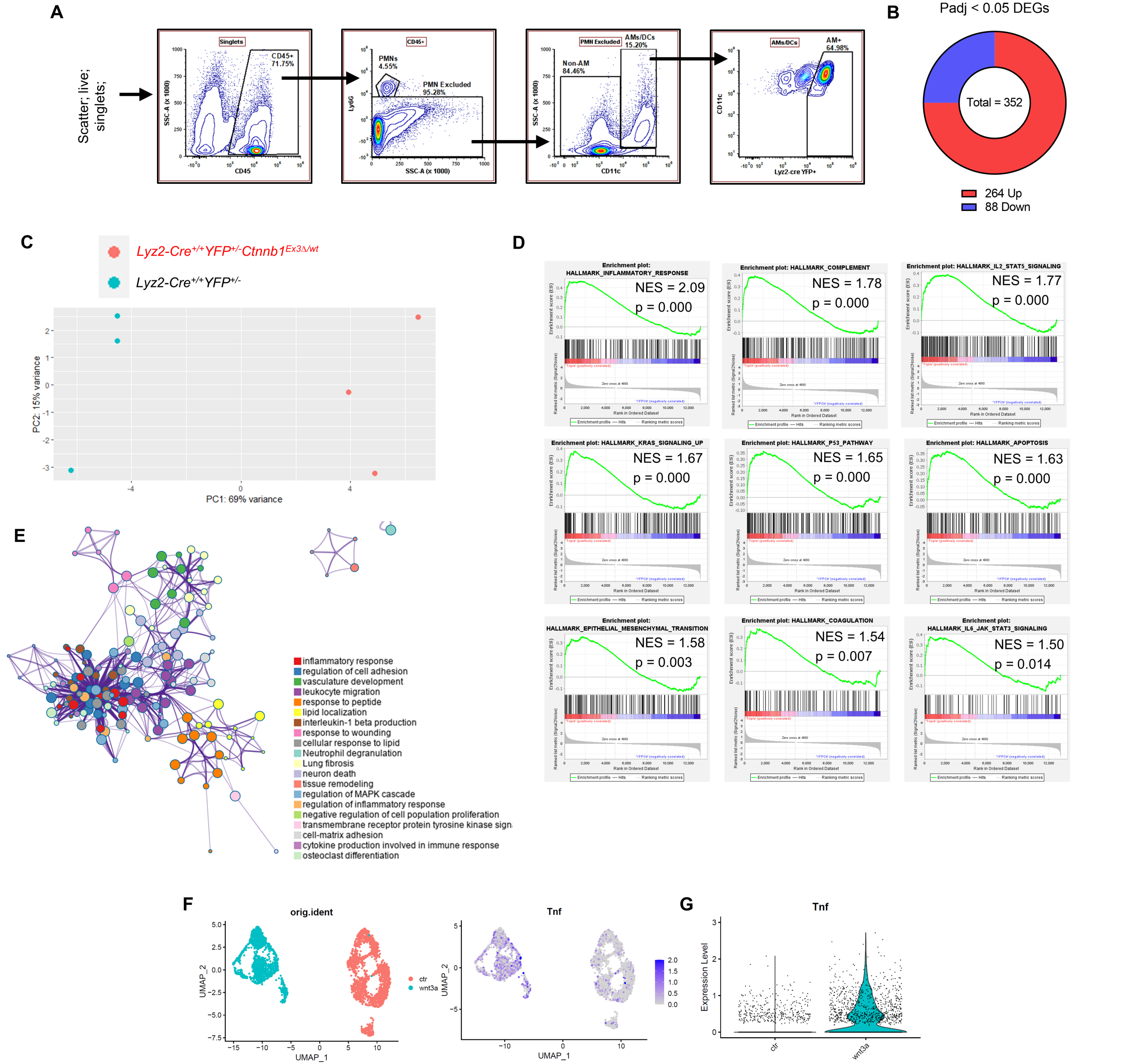

Supplemental Figure 4. Transcriptomic analyses of flow-sorted AMs. (A) Flow cytometry-assisted cell sorting gating strategy for AM isolation for bulk RNA-seq from lungs of non-tumor bearing *Lyz2-Cre*$^{+/+}$*YFP*$^{+/-}$*Ctnnb1*$^{Ex3Δ/wt}$ or *Lyz2-Cre*$^{+/+}$*YFP*$^{+/-}$ mice. **(B)** Number of DEGs from *A* with adjusted p-value < 0.05 up- or down-regulated in *Lyz2-Cre*$^{+/+}$*YFP*$^{+/-}$*Ctnnb1*$^{Ex3Δ/wt}$ AMs depicted in *Figure 4A*. **(C)** Principal component analysis (PCA) of variable genes between biological replicates. **(D)** GSEA plots for pathways identified in *Figure 4C*. **(E)** Metascape Gene Ontology analysis of significantly (p-adj > 0.05) upregulated DEGs in *Lyz2-Cre*$^{+/+}$*YFP*$^{+/-}$*Ctnnb1*$^{Ex3Δ/wt}$ AMs. **(F)** *Left*, single-cell RNA-seq of control (ctr) or Wnt3a-treated (wnt3a) BAL-derived AMs from Zhu et al. (GSE164793) UMAP, *right*, *Tnf* expression Featureplot. **(G)** *Tnf* expression in ctr vs. wnt3a from *F*; p = 1.21 x 10^{-100}, Wilcox rank sum test.

Supplemental Figure 5

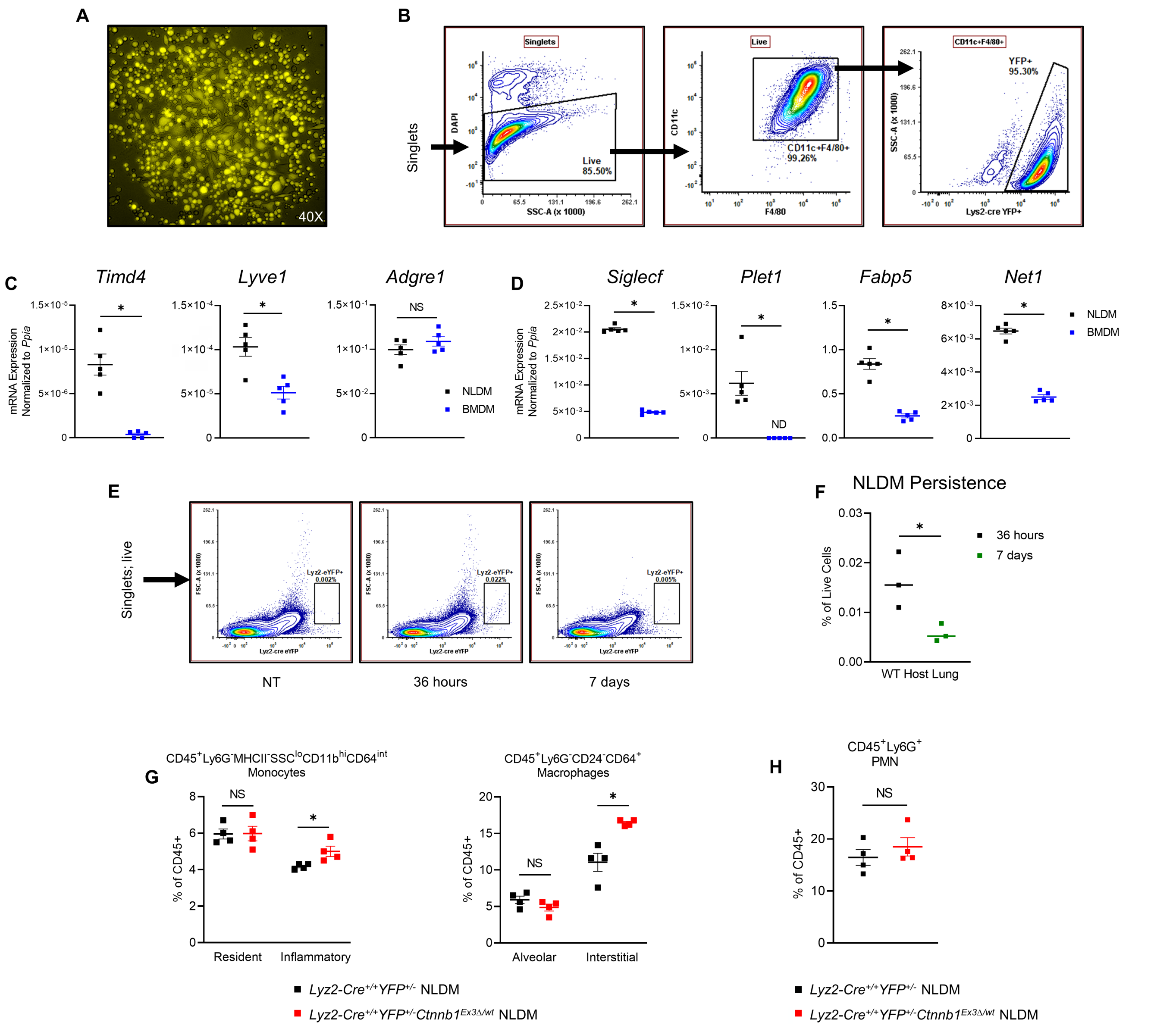

Supplemental Figure 5. Characterization of the NLDM primary cell line model. (A) Fluorescence microscopy image of YFP expression in unstimulated *Lyz2-Cre*$^{+/+}$*YFP*$^{+/-}$-derived NLDMs after 14 days of culture at 40X original magnification, and **(B)** flow cytometry gating of *Lyz2-Cre*$^{+/+}$*YFP*$^{+/-}$ NLDMs from *A,* representative of 5 biological replicates. **(C)** RT-qPCR of *Timd4*, *Lyve1*, and *Adgre1* (F4/80) of unstimulated *Lyz2-Cre*$^{+/+}$*YFP*$^{+/-}$NLDMs or BMDMs, collected after 14 and 6 days in culture, respectively. **(D)** RT-qPCR of *Siglecf*, *Plet1*, *Fabp6*, and *Net1* in *Lyz2-Cre*$^{+/+}$*YFP*$^{+/-}$NLDMs or BMDMs from *C*; n = 5; 2-tailed *t*-test. **(E)** Flow cytometry plots of WT C57BL/6 hosts following no treatment (NT) or intranasal adoptive transfer of *Lyz2-Cre*$^{+/+}$*YFP*$^{+/-}$NLDMs 36 hours or 7 days prior, representative of 3 biological replicates. **(F)** Quantification of the relative abundance of *Lyz2-Cre*$^{+/+}$*YFP*$^{+/-}$NLDMs from *E* at 36 hours or 7 days post-intranasal transfer; n = 3, 2-tailed *t*-test. **(G)** Abundance of macrophages and monocytes from *Figure 5G* at endpoint and **(H)** abundance of PMNs, as in *G*; n = 5, 2-tailed *t*-test. Data are recorded as the mean ± SEM of the indicated data points; * = $p < 0.05$, NS = not significant.

Supplemental Figure 6

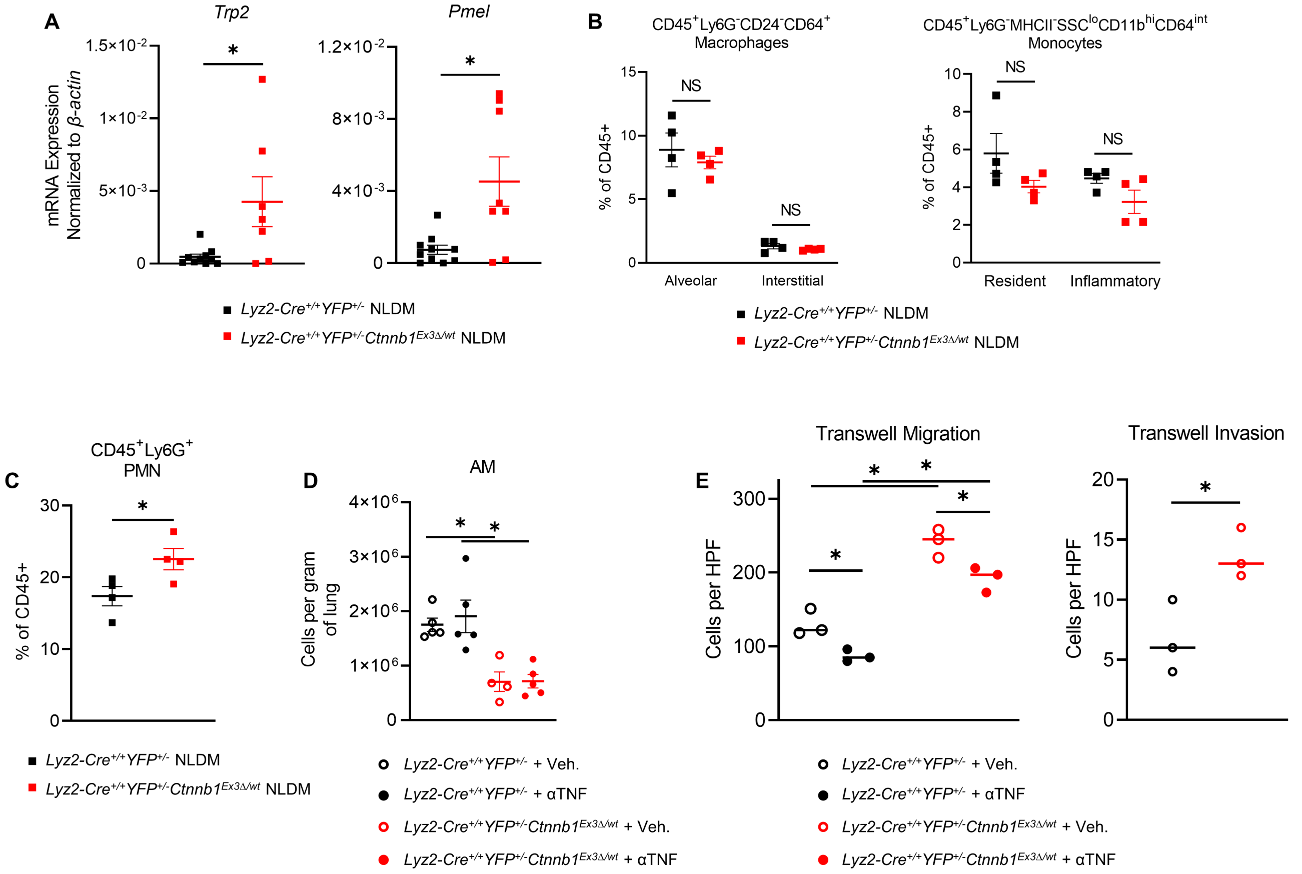

Supplemental Figure 6. Localized adoptive transfer of β-catenin activated NLDMs increases experimental metastasis in B16F10, and pathway activation promotes E0771.ML-1 tumor cell migration and invasion in vitro. (A) RT-qPCR mRNA expression of *left, Trp2* and *right*, *Pmel* from single cell-digested lungs from B16F10 via tail vein, via the same treatment protocol described in *Figure 5F*; n = 7-10, 2-tailed *t*-test. **(B & C)** Lung immune cell abundances from mice from *A*; n = 5, 2-tailed *t*-test. **(D)** Absolute abundance of $CD45^{+}Ly6G^{-}CD24^{-}CD64^{+}CD11b^{lo}CD11c^{+}$ AMs from *Figure 6B*. **(E)** *Left,* migration of E0771.ML-1 cells across 8µm Transwell insert after 8 hr incubation with *Lyz2-Cre*$^{+/+}$*YFP*$^{+/-}$*Ctnnb1*$^{Ex3\Delta/wt}$ or *Lyz2-Cre*$^{+/+}$*YFP*$^{+/-}$ NLDM supernatant untreated or with 50µg/mL αTNF antibody; n = 3, 2-tailed *t*-test. **(F)** Invasion of E0771.ML-1 cells across a Matrigel matrix in a Transwell assay as in *E*; n = 3, 2-tailed *t*-test. Data are recorded as the mean ± SEM of the indicated data points; * = $p < 0.05$, NS = not significant.

Kramer et al. Supplemental Table 1

Positive log2FoldChange = UP regulated in Lyz2-Cre+/+YFP+/-Ctnnb1Ex3Δ/wt relative to Lyz2-Cre+/+YFP+/-

Gene	baseMean	log2FoldChange	pvalue	padj
Cd63	511.523841	2.212904627	2.44E-61	3.13E-57
Lyz1	1335.362456	4.86082454	1.59E-47	1.02E-43
Pla2g7	291.3475414	2.731331563	8.90E-45	3.79E-41
Mfge8	330.0757964	2.494378757	2.08E-43	6.64E-40
Abca1	1438.455281	1.82760092	1.56E-34	3.99E-31
Gpnmb	2925.583021	1.643355321	4.86E-33	1.04E-29
C3ar1	189.4112946	2.520754581	1.38E-32	2.26E-29
Mmp12	312.4678463	2.270506622	1.41E-32	2.26E-29
Gm14023	540.3342772	1.56947105	1.47E-30	2.09E-27
Tnf	698.895157	1.669110551	1.04E-28	1.33E-25
Il1a	415.5235523	1.533822903	1.87E-27	2.18E-24
Pdk4	120.5510752	2.468112766	1.06E-24	1.13E-21
Ctsk	1214.446215	1.314834509	1.62E-24	1.60E-21
Scd1	179.1578873	2.20841949	5.63E-24	5.15E-21
F10	1454.010454	1.100026823	3.89E-22	3.32E-19
Igf1	249.9931147	2.15872812	1.47E-21	1.17E-18
Clec5a	242.4683148	1.457977615	2.77E-20	2.08E-17
Emp1	425.7624357	1.33900852	7.13E-20	5.07E-17
Myo5a	1071.8173	0.993265731	2.41E-19	1.62E-16
Pdpn	54.57966517	3.297076593	1.67E-18	1.07E-15
Ecm1	127.4824459	2.152154606	2.06E-18	1.25E-15
Anpep	323.8800874	1.54158401	2.69E-18	1.56E-15
Cxcl1	123.2539771	2.01375772	3.18E-18	1.77E-15
Nt5e	142.3646286	1.684671554	4.03E-18	2.15E-15
Fstl1	274.2827724	3.272783562	5.88E-18	3.01E-15
Ccl3	270.7711231	1.264395587	3.60E-17	1.77E-14
Fam20c	64.11635743	2.881892018	4.05E-17	1.92E-14
Ccl2	75.82379437	2.323980335	6.26E-17	2.86E-14
Mmp8	88.40495224	2.243636382	6.69E-17	2.95E-14
Spp1	1172.095097	1.047162759	7.43E-17	3.17E-14
Lepr	616.1997179	1.190321098	3.24E-16	1.34E-13
Il1rn	815.2761614	1.056126296	1.76E-15	7.03E-13
F7	1038.64245	-0.822855582	4.29E-15	1.66E-12
Cdh1	600.35251	1.158888304	4.01E-14	1.51E-11
Trem2	164.1189208	1.637538443	4.15E-14	1.52E-11
Scd2	363.5783942	1.235489894	5.07E-14	1.80E-11
Kat6b	553.2022551	1.006243964	7.08E-14	2.45E-11
Tnfaip3	1766.2206	1.084401321	1.01E-13	3.39E-11
Dock3	32.23485129	3.610090809	2.24E-13	7.34E-11
Emilin1	370.6766949	-1.203686455	2.99E-13	9.57E-11
Itgam	426.983276	1.043722953	3.17E-13	9.85E-11
Cspg4	179.4970869	1.569741983	3.23E-13	9.85E-11
Cpne5	254.2655271	-1.307411676	3.65E-13	1.09E-10
Colec12	277.5157441	1.465519098	4.43E-13	1.29E-10
Egr3	232.3326141	1.354920596	5.00E-13	1.42E-10
Malt1	759.8821227	0.889056463	8.23E-13	2.29E-10

Tmem119	64.32308891	2.273473235	9.46E-13	2.57E-10
Lilr4b	706.9661352	0.8443959	1.11E-12	2.96E-10
Bhlhe40	2116.614565	0.70809137	1.28E-12	3.35E-10
Stra6l	104.3573819	1.732718078	1.87E-12	4.79E-10
Sema3e	224.5740091	1.252047019	2.35E-12	5.88E-10
Gdf15	286.1405763	1.077867446	2.39E-12	5.88E-10
1700071M16Rik	95.00409777	1.950725672	2.54E-12	6.13E-10
Syngr1	31.63454441	4.217249544	3.57E-12	8.45E-10
Nfkbiz	1216.258958	0.859537712	4.00E-12	9.31E-10
Fpr2	229.898555	-1.17599588	5.83E-12	1.33E-09
Rab44	988.0749646	-0.934910802	1.00E-11	2.25E-09
Tnfsf9	58.70895833	2.013544795	1.11E-11	2.46E-09
Nlrp3	919.5025815	0.950870247	1.48E-11	3.20E-09
Lgmn	952.8442623	0.790305261	2.34E-11	5.00E-09
Rgcc	280.6411466	1.077981682	4.02E-11	8.44E-09
Gm16907	206.7768982	1.212433227	4.67E-11	9.63E-09
Ccl7	28.9351389	3.456011443	5.58E-11	1.13E-08
Mt1	695.5930302	0.730864224	7.17E-11	1.43E-08
Pltp	1066.456118	0.927334325	9.47E-11	1.86E-08
Cxcl2	1052.994831	0.888408788	9.87E-11	1.91E-08
Fn1	8743.593131	0.948198234	1.01E-10	1.94E-08
Dysf	221.5522872	1.132089143	1.69E-10	3.17E-08
Fmn1	444.1833629	0.809347868	3.07E-10	5.70E-08
Cd200r2	21.60248675	4.18350743	7.94E-10	1.45E-07
Slc6a8	54.09202152	1.98510966	8.25E-10	1.49E-07
Dhcr24	164.8107492	-1.462917784	8.62E-10	1.53E-07
Lhfpl2	356.3136375	0.902602941	1.02E-09	1.79E-07
Socs3	422.6195123	0.924079814	1.11E-09	1.92E-07
Egr1	2519.524823	0.766013592	1.57E-09	2.68E-07
Zranb3	58.30391251	1.810361248	1.73E-09	2.91E-07
Ptafr	92.41324153	1.443683017	2.58E-09	4.28E-07
Atf3	918.3302069	0.6962881	2.78E-09	4.56E-07
Gas7	246.0962488	0.971490099	3.30E-09	5.29E-07
Plau	179.1510369	1.374803949	3.31E-09	5.29E-07
2010111I01Rik	379.5051153	0.970740471	3.52E-09	5.56E-07
C77080	734.4695389	0.814460331	3.92E-09	6.12E-07
Ccrl2	399.8735826	1.002548358	5.79E-09	8.93E-07
Slc7a11	131.3128868	1.205592317	6.74E-09	1.03E-06
Fosb	2457.053855	0.669629466	7.63E-09	1.15E-06
Hk2	491.809239	0.901988388	7.93E-09	1.18E-06
Ksr2	21.58150624	3.216542368	8.57E-09	1.26E-06
Adgrl3	259.259717	-0.889311737	1.20E-08	1.74E-06
Slc37a2	106.5551293	1.274104763	1.27E-08	1.82E-06
Msr1	118.1992911	1.319974396	1.33E-08	1.89E-06
Dkk2	21.31439686	5.413967266	1.51E-08	2.12E-06
Fam3c	442.1403282	0.764783066	1.73E-08	2.40E-06
Fancc	109.8308209	1.285801292	1.90E-08	2.62E-06
Rgs7bp	24.98999564	3.090087146	1.93E-08	2.62E-06
Mylip	425.4907409	0.80127722	2.39E-08	3.22E-06

Cd300a	204.0900148	0.917040752	2.71E-08	3.61E-06
Lilrb4a	1461.7566	0.64889532	2.84E-08	3.74E-06
Bcl2l11	465.9187919	0.708708336	4.52E-08	5.90E-06
Nr4a3	1291.106516	0.796610169	5.11E-08	6.60E-06
Sgms2	149.1509638	1.031632338	6.05E-08	7.74E-06
Pcyox1	1688.976942	0.604299167	6.15E-08	7.79E-06
Igf2bp2	86.00770996	-1.329133857	6.37E-08	7.99E-06
Arl4c	259.6423166	0.896854055	8.84E-08	1.09E-05
Ucp3	227.5470412	-0.848031045	8.87E-08	1.09E-05
Acot1	123.649609	1.261405632	1.14E-07	1.39E-05
Txnip	3542.334356	-0.476189429	1.48E-07	1.79E-05
Phlda1	921.0941966	0.636644884	1.50E-07	1.79E-05
Fpr1	479.0939733	-0.713346679	2.61E-07	3.09E-05
Fyco1	690.1736896	-0.603998029	2.81E-07	3.30E-05
Tgif1	291.562229	0.761008129	2.86E-07	3.32E-05
3110021N24Rik	72.35619407	-1.464073591	3.35E-07	3.86E-05
Coro6	318.8380099	-0.867479579	3.86E-07	4.41E-05
Marco	2230.362333	0.61682057	4.61E-07	5.18E-05
Nr4a1	2097.167341	0.540600236	4.61E-07	5.18E-05
Igf2bp3	89.60033172	1.243891658	4.86E-07	5.40E-05
Krt79	866.8925953	-0.638186232	5.14E-07	5.63E-05
Neat1	14443.73896	0.467485747	5.15E-07	5.63E-05
Ccl9	208.5783701	1.055447629	5.87E-07	6.36E-05
Txnrd1	780.7458267	0.584603809	6.00E-07	6.45E-05
Slc1a3	28.09068579	2.339751833	6.17E-07	6.53E-05
Sash1	385.4242896	0.754563768	6.17E-07	6.53E-05
Tbc1d4	336.0515907	0.69725476	6.56E-07	6.88E-05
Gm20594	54.03905991	1.830136024	7.08E-07	7.37E-05
Tsc22d3	356.6406828	-0.845749947	7.47E-07	7.71E-05
Litaf	716.5335998	0.609878793	7.86E-07	8.02E-05
Entpd3	9.220204965	6.686189198	7.95E-07	8.02E-05
Cldn1	149.1920131	-1.008611632	7.96E-07	8.02E-05
Eps8	357.5540167	0.918068077	8.05E-07	8.05E-05
Plxdc2	458.8926517	0.63860327	8.66E-07	8.59E-05
Lyz2	128.6052381	-0.981284541	8.77E-07	8.63E-05
Ier3	79.69983921	1.403456136	8.89E-07	8.68E-05
Serpinb6a	498.4239313	0.625330778	9.12E-07	8.84E-05
Slc24a1	101.7891609	1.293573604	1.33E-06	0.000127618
Zfand2a	529.6280276	0.587966485	1.65E-06	0.000157707
Nrp2	939.8479564	0.532396532	1.83E-06	0.000173182
Actn1	1806.737178	0.543393289	1.98E-06	0.00018654
Apoe	4909.018788	2.420389654	2.00E-06	0.00018654
Nfkbia	1186.616459	0.747834568	2.02E-06	0.000187201
Cd14	779.8311648	0.669841391	2.31E-06	0.0002127
Cotl1	3672.085248	0.51632119	2.53E-06	0.000231159
Sipa1l1	297.4029298	-0.746272557	2.77E-06	0.00025111
Adamtsl4	413.5652488	0.684812959	2.97E-06	0.000267209
Erdr1	783.3033438	0.753832984	3.47E-06	0.000310692
Lpl	6681.353309	0.444940445	3.69E-06	0.000327675

Wls	212.6316189	0.749966052	4.02E-06	0.000354532
Sik1	409.3057693	-0.735315869	4.07E-06	0.000356377
Cd74	5659.705172	-0.622952183	4.68E-06	0.000407681
Mapkapk2	1090.271126	0.575083271	5.16E-06	0.000445679
Srxn1	107.5309642	0.978873043	5.67E-06	0.000486813
Chil3	14165.15018	-0.424253727	5.77E-06	0.00049202
0610040F04Rik	257.9232065	0.731154249	6.65E-06	0.000563425
Clec4d	64.18102688	1.319448502	6.71E-06	0.00056442
Jag1	271.1004318	0.653415816	7.25E-06	0.000606015
Trem1	212.4101691	0.762571196	7.30E-06	0.000606571
Cdk5r1	13.87909558	3.29108157	7.35E-06	0.000606571
Ptprs	245.2522417	-0.718293345	7.98E-06	0.000654876
Ptgs1	78.849922	1.116081385	8.83E-06	0.000719956
Gm15441	2209.181458	-0.41230482	9.03E-06	0.000731053
Slc7a8	200.6375249	0.735990928	9.83E-06	0.000787569
Cdkn1a	679.8403052	0.58894518	9.85E-06	0.000787569
Lasp1	891.6634593	0.49370381	1.09E-05	0.000870208
Abcb1b	34.35035795	1.698627872	1.13E-05	0.000896119
Il10	34.4959916	1.668922523	1.15E-05	0.00089883
Hmgn5	332.7039204	0.658771368	1.23E-05	0.000961721
Ninj1	35.02584166	1.600827348	1.31E-05	0.00101436
Apoc1	16.22639691	2.64116069	1.35E-05	0.001040605
Rdh5	27.83724647	1.898492866	1.38E-05	0.001059641
Rell1	98.43404154	1.072780508	1.44E-05	0.001097751
Acpp	16.06504539	2.990074346	1.48E-05	0.001121801
Tm7sf3	169.0371384	0.786585156	1.59E-05	0.001199149
Cacfd1	89.98791436	1.027087059	1.70E-05	0.001271583
Cebpb	6097.574947	-0.450308288	1.75E-05	0.00130262
Ffar2	105.9379521	1.012077214	1.81E-05	0.001336221
Ccbe1	61.75211465	1.192439028	1.91E-05	0.001405926
Ccdc146	60.2305921	1.178609872	1.99E-05	0.001452469
Hmox1	501.7459331	0.568465048	2.03E-05	0.001476942
Clmn	725.6330544	-0.4847814	2.24E-05	0.001620711
Fabp1	1096.745037	0.536292726	2.36E-05	0.001693054
Bzw2	437.3093617	0.593439429	2.59E-05	0.001850099
Zbtb16	124.5585107	-1.097237491	2.81E-05	0.001996106
Parvb	332.1899122	0.696139733	2.87E-05	0.002024558
Bzrap1	78.97894037	1.076650942	2.88E-05	0.002024558
Nr4a2	407.5035079	0.614907814	2.95E-05	0.002061122
1600023N17Rik	10.32235074	3.905123373	3.18E-05	0.002211057
Ank	100.8286849	0.910481977	3.44E-05	0.002377611
Kcnn4	51.95970954	1.293046657	3.63E-05	0.002496336
Lgals9	188.9281276	0.718193658	3.74E-05	0.002540122
Alcam	289.8796527	0.634518915	3.75E-05	0.002540122
Rps14	815.1968507	-0.603771229	3.75E-05	0.002540122
Klra2	339.807746	-0.685147181	3.79E-05	0.002550652
Krt80	130.5639358	-0.845651545	3.87E-05	0.002589878
Ccdc88c	1281.493528	-0.450929261	3.94E-05	0.00262645
Rpph1	60940.42194	-0.585629774	4.08E-05	0.002707056

Lrp12	1028.93643	0.471408046	4.25E-05	0.002801442
St6gal1	217.6639944	0.669836136	4.47E-05	0.00293451
Clec4e	143.6062051	0.877935039	4.55E-05	0.002969999
Tanc2	325.6751725	-0.633171518	4.61E-05	0.002983924
Lrrc27	12.4439706	2.751540205	4.62E-05	0.002983924
Ifrd1	984.1896008	0.494020699	4.80E-05	0.003089122
Plcl1	44.77649162	-1.456237475	4.89E-05	0.003129207
Cyth4	1098.46105	0.582900832	5.32E-05	0.003383755
Mafb	386.3813099	0.627584199	5.70E-05	0.003611907
Sema6d	309.3616818	-0.606930342	5.90E-05	0.00371673
Itgae	259.7459104	-0.679975172	5.94E-05	0.003726005
Fcrls	35.76447264	1.458977399	6.09E-05	0.003799564
Csf1	50.01797768	1.214861164	6.28E-05	0.003898025
Prickle2	440.3571023	0.5738612	6.58E-05	0.004065109
Cpeb1	14.21117436	2.644941802	6.63E-05	0.004080776
H2-Aa	1332.978874	-0.507432356	6.67E-05	0.004084236
Cytip	2431.422649	-0.428572454	7.26E-05	0.004425191
Dusp6	449.2918902	0.523289776	7.31E-05	0.004433338
Tmem134	177.5759284	-0.731986262	7.35E-05	0.004435806
Gbp8	119.2627488	-1.019025913	7.38E-05	0.004435806
Il1b	510.7890324	0.643616127	7.58E-05	0.004530324
Galnt3	374.1719625	0.635477512	7.79E-05	0.004626376
Epcam	76.14584656	-1.015550368	7.81E-05	0.004626376
Cd33	596.5262757	0.519687728	7.88E-05	0.004648245
Dlec1	185.3959594	0.889374451	8.00E-05	0.004696507
Espn	32.18806535	-1.48825443	8.32E-05	0.004861208
Fyn	350.5554485	-0.580014479	8.77E-05	0.0050984
Prdm8	17.12513988	2.411416412	8.87E-05	0.005134235
Acot2	98.67509086	0.900107366	9.50E-05	0.005476562
Kdm5b	125.9348982	-0.797250331	9.55E-05	0.005478777
Sh3pxd2b	1174.287446	0.465933001	0.000101031	0.005770957
Ccr1l1	7.043357189	5.308889711	0.000107891	0.006135388
Foxp4	89.86123491	-1.027129535	0.000110475	0.006192311
Dgkz	1001.113121	0.428131364	0.000110492	0.006192311
1110007C09Rik	90.69603494	0.904911019	0.000110557	0.006192311
Ccdc171	163.9414071	-0.713329961	0.000110967	0.006192311
Src	61.61819236	1.249085548	0.000111312	0.006192311
Pim1	1198.503219	0.540569213	0.000112474	0.006203722
Emb	95.17720139	-0.887790555	0.000112486	0.006203722
Lrrc3	22.881743	-1.814075425	0.000115219	0.006327134
Proz	45.01793228	1.282777076	0.000124496	0.006807355
Mamdc2	158.2787212	0.770638421	0.00012822	0.006981195
Cbfa2t3	552.033971	-0.565757983	0.000132708	0.007194912
Chsy1	426.6412471	-0.513057945	0.00013765	0.007431361
Tmem38b	200.4700771	0.706924449	0.000145074	0.007799246
Oxct1	355.0553127	0.549568275	0.000147339	0.007887875
Fam71a	57.77122141	1.132219227	0.000149531	0.007971874
H2-Eb1	1660.933951	-0.62199252	0.000163098	0.008659076
Ppp1r15a	510.3925743	0.559217397	0.000171967	0.009092232

Lox	13.5103631	2.302541148	0.000176809	0.009309734
Ptch2	35.31781963	1.361881911	0.000178403	0.009355166
Cd68	1840.069369	0.419873659	0.000181691	0.009488744
Cdk6	503.9545375	-0.482820936	0.000183775	0.009558519
Hsd3b7	550.0413267	-0.450585128	0.000192353	0.009964212
Olfr112	8.312815631	3.544655309	0.000195823	0.010103038
Wfdc17	36.64710237	1.416798912	0.00019902	0.010226734
Tsku	11.3451939	2.733784378	0.000205015	0.01049267
Rasgrp1	116.8423082	0.838892801	0.000231467	0.011799263
Slc39a2	302.3835901	0.566496497	0.000242523	0.01231383
Cpeb4	632.3829683	0.53300991	0.00024576	0.012428839
Clec2d	453.5744812	-0.519977243	0.000251503	0.012669239
4930473A02Rik	184.3482691	0.66591988	0.000254094	0.01274953
Gla	152.3757473	0.682232757	0.000263613	0.013127031
Csrnp1	311.8614382	0.686980525	0.000263669	0.013127031
Cib2	87.63106053	-0.935756937	0.000265517	0.013167782
Iqgap2	685.9360286	-0.428969316	0.000266705	0.013175645
Mki67	1112.355307	0.480971413	0.000268752	0.013225706
Hilpda	89.64443688	0.976924214	0.00027045	0.013258248
Vstm2a	167.0680884	0.730577319	0.000273869	0.013374646
Atp1b3	655.199227	0.485736946	0.000285069	0.01386865
Myo1e	971.1559698	0.39219273	0.000292754	0.014153235
Igflr1	68.85058464	-1.136401301	0.000293131	0.014153235
Arpc4	785.3822666	0.4326514	0.000294815	0.014181051
Npas2	3.895578835	5.445216553	0.000299474	NA
Mdm2	428.4339847	0.468153457	0.000306601	0.014692721
Htra3	37.49206873	1.277754223	0.000309749	0.014788187
Slc27a1	268.656981	0.736038088	0.000321466	0.015290548
Mlph	117.2861573	0.929010195	0.000326595	0.015476954
Mvb12b	184.1089092	0.655260011	0.000331232	0.015638805
Slc2a1	105.230647	0.811612982	0.000337461	0.015874292
Ctsa	2748.926105	0.384920727	0.00033992	0.015931416
Zfp276	281.3218829	-0.554143088	0.000369524	0.017247095
Gramd1b	103.6375553	0.75746269	0.000370688	0.017247095
H2-Ab1	2881.25436	-0.451514751	0.000374556	0.017363923
Rhoc	210.493282	-0.635579159	0.00037702	0.017383355
Psmd8	487.7707772	0.478607112	0.000377692	0.017383355
Slfn10-ps	18.36655857	1.80556201	0.000382786	0.017554626
Ciita	379.1751857	-0.640852929	0.000387091	0.017688655
Anxa4	841.8530665	0.423663528	0.000388721	0.017699929
Ppp1r9a	164.8353005	-0.707074922	0.000402402	0.01825794
Net1	2027.499869	-0.354563298	0.000406986	0.018400651
Gabbr1	111.7546386	-0.788862902	0.000415187	0.018684563
Map6	105.3595942	0.870987073	0.000416186	0.018684563
Med22	175.3436659	0.614862682	0.000440656	0.019713952
Penk	18.24983499	1.863996036	0.000446605	0.019857609
Rab7	1321.828316	0.372611943	0.000446971	0.019857609
E2f2	167.9906152	0.671524624	0.000453309	0.020069494
Pfn1	2618.378905	0.359665538	0.000460394	0.020312916

Egr2	1170.587697	0.445467309	0.000467181	0.020541516
Abl2	493.4540127	0.453091104	0.000470901	0.020634174
3010026O09Rik	60.694734	1.005113287	0.00047449	0.020712704
Elovl6	68.65540993	-1.049721031	0.000475931	0.020712704
Fam19a5	3.544247918	5.309715027	0.000482324	NA
Tmc8	31.51526076	-1.32115731	0.000496338	0.021527626
Slc3a2	1109.473372	0.370440789	0.000514243	0.022166387
Zfyve9	52.3509639	-1.12194888	0.00051453	0.022166387
Lpcat2	111.272692	0.767400635	0.000536539	0.02301827
Rsrp1	1438.365083	-0.383145928	0.000537903	0.02301827
Bhlhe41	1137.881305	0.347321663	0.000548392	0.023388914
Tmem132a	991.1065227	0.471901271	0.000562229	0.02389939
Rai14	144.7464774	1.733305119	0.000593027	0.025096362
Gsn	1238.228863	0.368239978	0.00059431	0.025096362
Ptgr1	46.75751065	1.083050787	0.00059787	0.025142422
Rasgef1b	1085.121666	0.389009918	0.000599331	0.025142422
Snord57	33.87830282	1.664416001	0.000601528	0.025152117
Spag11b	40.00371192	1.30821324	0.000607073	0.02530131
Hr	139.9534635	-0.745713668	0.000624461	0.025941504
Zak	296.8725734	0.550541147	0.000629338	0.026059494
Adgrg1	59.27104689	-1.075499243	0.000638494	0.026353324
Gpr3	9.409023595	2.632733476	0.000646701	0.026606245
Adam8	171.0269833	-0.766320503	0.000653841	0.026813756
8430408G22Rik	255.9338078	-0.55825149	0.000689988	0.028205749
Fnip2	918.9524366	0.422308104	0.000697003	0.028342396
Tbc1d10a	173.073052	-0.661076809	0.000697761	0.028342396
Ccl5	31.53624831	-1.355211057	0.000707177	0.028633968
Cidec	612.6633049	-0.500708442	0.000734686	0.02965396
Osbpl3	246.4179787	0.562089531	0.000742798	0.029887094
Erc2	172.1933594	-0.640144458	0.000749314	0.030054778
F630028O10Rik	86.16366377	-0.987248143	0.000776898	0.031063773
Ttc21a	12.87911386	2.323164635	0.000785882	0.031325129
Frk	42.81183939	1.124036531	0.000793922	0.031547311
Arhgap26	1166.632498	-0.358232229	0.000827159	0.032766263
Adcy6	123.3565489	-0.778622313	0.000837557	0.033075739
Sgk1	3285.100341	0.315255074	0.000855258	0.033670846
Slc29a1	469.7453763	0.447682366	0.000859566	0.033730707
Rnf128	19.3914454	1.746062627	0.000862051	0.033730707
Trim24	120.1509654	-0.697731283	0.000868654	0.033885442
Sestd1	113.613238	0.693217361	0.000885614	0.034442059
Fblim1	166.6179094	0.665857385	0.000892345	0.034598654
B4galt1	591.698331	0.447513401	0.000930238	0.035958892
4930599N23Rik	99.3853517	0.738570753	0.000939396	0.036203538
Pid1	228.3722588	0.535604912	0.000992652	0.038088322
Timp2	446.288024	0.509348373	0.000994256	0.038088322
Clec4n	822.6119087	0.413605317	0.000997871	0.038112726
Hip1r	277.7114843	-0.530685282	0.001004355	0.038246188
Gatm	57.62506657	-1.040711179	0.001027189	0.038999648
Prelid2	18.12879584	-1.637414916	0.00104136	0.0394207

Rmi1	330.5014138	0.530182493	0.001100231	0.041526413
Herpud1	392.8477209	-0.448880735	0.001146955	0.043162628
Rbpj	682.2958282	0.429436857	0.001159579	0.043509704
Clec1b	44.27619145	1.104420765	0.001187861	0.044440593
Plbd1	415.6287602	-0.472488955	0.00119344	0.044519129
Ctss	9956.250544	-0.282064695	0.001210794	0.04503522
AW112010	47.71180856	-1.065171917	0.001224945	0.045429482
Lims1	333.2885545	0.446729758	0.001240148	0.045860379
4931440P22Rik	112.9634877	0.664044743	0.001261264	0.046506847
Nfe2l2	867.8763978	0.384967719	0.001342517	0.049307812
Fam212a	40.37336904	-1.093961902	0.001344934	0.049307812
Bcl2a1b	431.5062783	0.428964358	0.00136236	0.049803985

Kramer et al. Supplemental Table 2

UP regulated in Lyz2-Cre+/+YFP+/-Ctnnb1Ex3Δ/wt relative to Lyz2-Cre+/+YFP+/-

NAME	SIZE	ES	NES	NOM p-val	FDR q-val	FWER p-val
HALLMARK_TNFA_SIGNALING_VIA_NFKB	189	0.5228427	2.384836	0	0	0
HALLMARK_INFLAMMATORY_RESPONSE	175	0.4649009	2.0897958	0	0	0
HALLMARK_COMPLEMENT	171	0.391839	1.7815962	0	0.006942031	0.011
HALLMARK_IL2_STAT5_SIGNALING	191	0.38917518	1.7684631	0	0.006306523	0.013
HALLMARK_KRAS_SIGNALING_UP	172	0.37779653	1.6864816	0	0.012771722	0.032
HALLMARK_P53_PATHWAY	189	0.36350355	1.6497709	0	0.018496457	0.056
HALLMARK_APOPTOSIS	148	0.3675941	1.6284434	0	0.019855699	0.069
HALLMARK_EPITHELIAL_MESENCHYMAL_TRANSITION	166	0.35301563	1.5755601	0.003076923	0.030838158	0.118
HALLMARK_COAGULATION	104	0.37193736	1.5353906	0.007425743	0.036614947	0.151
HALLMARK_ESTROGEN_RESPONSE_LATE	165	0.29888755	1.3505424	0.011142061	0.12602551	0.53
HALLMARK_HYPOXIA	171	0.31036854	1.3913258	0.012539185	0.09488032	0.406
HALLMARK_IL6_JAK_STAT3_SIGNALING	79	0.380484	1.502002	0.013736264	0.043594502	0.199
HALLMARK_ESTROGEN_RESPONSE_EARLY	172	0.29139036	1.301258	0.033994336	0.14059843	0.666
HALLMARK_XENOBIOTIC_METABOLISM	160	0.30243286	1.3372833	0.0359116	0.12932126	0.566
HALLMARK_CHOLESTEROL_HOMEOSTASIS	69	0.34684932	1.3342023	0.05	0.12361256	0.572

Kramer et al. Supplemental Table 3

UP regulated in Lyz2-Cre+/+YFP+/-Ctnnb1Ex3Δ/wt relative to Lyz2-Cre+/+YFP+/-

NAME	SIZE	ES	NES	NOM p-val	FDR q-val	FWER p-val
GOBP_POSITIVE_REGULATION_OF_LIPID_STORAGE	22	0.7142092	2.195236	0	0.038392685	0.024
GOBP_REGULATION_OF_LIPID_STORAGE	47	0.60589755	2.1655	0	0.032107066	0.04
GOBP_PROTEIN_LIPID_COMPLEX_SUBUNIT_ORGANIZATION	37	0.63082993	2.161361	0	0.021929035	0.041
GOBP_REGULATION_OF_PLASMA_LIPOPROTEIN_PARTICLE_LEVELS	66	0.5652764	2.153499	0	0.019656403	0.049
GOBP_LIPID_STORAGE	74	0.5404545	2.1476483	0	0.017950531	0.056
GOBP_INTERLEUKIN_1_PRODUCTION	85	0.53418404	2.1451633	0	0.015495149	0.058
GOBP_NEGATIVE_REGULATION_OF_INTERLEUKIN_1_BETA_PRODUCTION	20	0.70793134	2.109485	0	0.024985926	0.107
GOBP_REGULATION_OF_HETEROTYPIC_CELL_CELL_ADHESION	17	0.7473678	2.1032362	0	0.023430523	0.115
GOBP_GRANULOCYTE_CHEMOTAXIS	87	0.5156702	2.099306	0	0.022272365	0.123
GOBP_CELLULAR_RESPONSE_TO_FATTY_ACID	27	0.6596066	2.0926316	0	0.022108605	0.135
GOBP_NEUTROPHIL_CHEMOTAXIS	73	0.5413211	2.084956	0	0.022296311	0.149
GOBP_INTERLEUKIN_1_BETA_PRODUCTION	73	0.53311205	2.083842	0	0.020839546	0.151
GOBP_NEGATIVE_REGULATION_OF_INTERLEUKIN_1_PRODUCTION	25	0.6686165	2.0820417	0	0.01997572	0.155
GOBP_HUMORAL_IMMUNE_RESPONSE	122	0.48140016	2.0662014	0	0.0226779	0.184
GOBP_TOLERANCE_INDUCTION	22	0.6735145	2.0622284	0	0.02234059	0.192
GOBP_POSITIVE_REGULATION_OF_LIPID_LOCALIZATION	84	0.51793027	2.057979	0	0.02244735	0.203
GOBP_POSITIVE_REGULATION_OF_MACROPHAGE_MIGRATION	21	0.69370306	2.0435889	0	0.026309734	0.246
GOBP_RESPONSE_TO_FATTY_ACID	45	0.5751703	2.0326958	0	0.02849401	0.281
GOBP_REGULATION_OF_BEHAVIOR	33	0.6074947	2.0325875	0	0.026994329	0.281
GOBP_AMYLOID_BETA_CLEARANCE	33	0.60367876	2.016608	0	0.031797167	0.337
GOBP_REGULATION_OF_LEUKOCYTE_DEGRANULATION	35	0.5936506	2.0141687	0	0.031356	0.346
GOBP_CELLULAR_RESPONSE_TO_MOLECULE_OF_BACTERIAL_ORIGIN	158	0.45164892	2.0049267	0	0.03321516	0.373
GOBP_NEUTROPHIL_MIGRATION	87	0.4910698	1.996466	0	0.03559136	0.402
GOBP_GRANULOCYTE_MIGRATION	103	0.4845869	1.991921	0	0.03558837	0.414
GOBP_MACROPHAGE_CHEMOTAXIS	30	0.61058	1.9879568	0	0.035635285	0.43
GOBP_CHEMOKINE_PRODUCTION	72	0.50569326	1.9827901	0	0.035762142	0.446
GOBP_REGULATION_OF_MEMBRANE_INVAGINATION	15	0.7247144	1.972934	0	0.038764838	0.487
GOBP_MEMBRANE_INVAGINATION	59	0.52217704	1.9694482	0	0.03869368	0.494
GOBP_REGULATION_OF_AMYLOID_BETA_CLEARANCE	15	0.6974186	1.9514387	0	0.04523159	0.563
GOBP_POSITIVE_REGULATION_OF_CHOLESTEROL_EFFLUX	17	0.69811726	1.949814	0	0.04464855	0.568
GOBP_CELLULAR_RESPONSE_TO_BIOTIC_STIMULUS	181	0.4274607	1.9492502	0	0.043400977	0.569
GOBP_POSITIVE_REGULATION_OF_CALCIUM_MEDIATED_SIGNALING	22	0.64697355	1.9319143	0	0.048236128	0.635
GOBP_LIPOPOLYSACCHARIDE_MEDIATED_SIGNALING_PATHWAY	52	0.53540415	1.923688	0	0.050501592	0.664
GOBP_CHOLESTEROL_EFFLUX	38	0.5527135	1.9178114	0	0.05128782	0.688
GOBP_OOGENESIS	51	0.53319746	1.9137099	0	0.05237539	0.708

GOBP_PROTEIN_LIPID_COMPLEX_ASSEMBLY	27	0.60371965	1.9123011	0	0.051741842	0.714
GOBP_REGULATION_OF_BONE_MINERALIZATION	54	0.52416265	1.9112884	0	0.05134138	0.719
GOBP_POSITIVE_REGULATION_OF_INFLAMMATORY_RESPONSE	97	0.45988822	1.9062433	0	0.0519487	0.745
GOBP_REGULATION_OF_MEMBRANE_PROTEIN_ECTODOMAIN_PROTEOLYSIS	22	0.6289227	1.9025965	0	0.052996513	0.763
GOBP_REGULATION_OF_PHAGOCYTOSIS	77	0.47859046	1.8882126	0	0.058024332	0.811
GOBP_TUMOR_NECROSIS_FACTOR_SUPERFAMILY_CYTOKINE_PRODUCTION	125	0.43808243	1.8854007	0	0.058389254	0.817
GOBP_POSITIVE_REGULATION_OF_PHAGOCYTOSIS	54	0.5089912	1.8841945	0	0.058011796	0.82
GOBP_BONE_MINERALIZATION	83	0.4707862	1.879688	0	0.05957849	0.841
GOBP_REGULATION_OF_LEUKOCYTE_CHEMOTAXIS	96	0.44335753	1.8694031	0	0.059242494	0.869
GOBP_FEMALE_GAMETE_GENERATION	85	0.46600312	1.8662187	0	0.06038782	0.877
GOBP_VASCULAR_ENDOTHELIAL_GROWTH_FACTOR_PRODUCTION	35	0.5564537	1.8633057	0	0.06108908	0.885
GOBP_POSITIVE_REGULATION_OF_INTERLEUKIN_1_PRODUCTION	46	0.52770305	1.8603383	0	0.062071137	0.891
GOBP_PLASMA_LIPOPROTEIN_PARTICLE_CLEARANCE	45	0.52530813	1.8543202	0	0.06368413	0.903
GOBP_REGULATION_OF_ANION_TRANSMEMBRANE_TRANSPORT	82	0.44910264	1.844457	0	0.06693039	0.921
GOBP_REGULATION_OF_OSSIFICATION	82	0.45753843	1.8442296	0	0.066098765	0.922
GOBP_REGULATION_OF_MONONUCLEAR_CELL_MIGRATION	89	0.45200035	1.8435569	0	0.065540776	0.923
GOBP_POSITIVE_REGULATION_OF_LEUKOCYTE_MIGRATION	108	0.43615618	1.8419499	0	0.0646927	0.929
GOBP_POSITIVE_REGULATION_OF_MYELOID_LEUKOCYTE_MEDIATED_IMMUNI	16	0.65987575	1.838017	0	0.06623854	0.932
GOBP_GLIAL_CELL_MIGRATION	41	0.52046454	1.8361577	0	0.066181235	0.933
GOBP_NEGATIVE_REGULATION_OF_TUMOR_NECROSIS_FACTOR_SUPERFAMILY	46	0.50906384	1.8340474	0	0.06545545	0.939
GOBP_POSITIVE_REGULATION_OF_NIK_NF_KAPPAB_SIGNALING	61	0.4868398	1.8257877	0	0.070531845	0.954
GOBP_RESPIRATORY_SYSTEM_DEVELOPMENT	154	0.40669817	1.8161813	0	0.07312073	0.967
GOBP_REGULATION_OF_LIPID_LOCALIZATION	129	0.42530516	1.8141122	0	0.07386671	0.97
GOBP_MULTICELLULAR_ORGANISMAL_RESPONSE_TO_STRESS	37	0.51074505	1.8118831	0	0.07457302	0.971
GOBP_REGULATION_OF_TISSUE_REMODELING	60	0.47717002	1.8078079	0	0.07525903	0.971
GOBP_BIOMINERALIZATION	106	0.4325073	1.8033198	0	0.07759553	0.973
GOBP_INTERLEUKIN_6_PRODUCTION	117	0.4271417	1.8008862	0	0.07839181	0.973
GOBP_RESPONSE_TO_PROGESTERONE	33	0.55218357	1.79864	0	0.07894557	0.974
GOBP_POSITIVE_REGULATION_OF_REACTIVE_OXYGEN_SPECIES_METABOLIC_P	77	0.4478671	1.7875228	0	0.08389782	0.982
GOBP_MYELOID_LEUKOCYTE_MIGRATION	160	0.3998551	1.7838552	0	0.08397873	0.985
GOBP_REGULATION_OF_REPRODUCTIVE_PROCESS	100	0.42551568	1.7688855	0	0.08854202	0.994
GOBP_POSITIVE_REGULATION_OF_EPITHELIAL_TO_MESENCHYMAL_TRANSITIO	43	0.49678436	1.7509289	0	0.09803897	0.997
GOBP_RESPONSE_TO_MOLECULE_OF_BACTERIAL_ORIGIN	259	0.3665905	1.74821	0	0.09968595	0.997
GOBP_ERK1_AND_ERK2_CASCADE	222	0.37461895	1.739869	0	0.104001425	0.999
GOBP_POSITIVE_REGULATION_OF_LEUKOCYTE_CHEMOTAXIS	73	0.44826725	1.7285804	0	0.10589663	0.999
GOBP_REGULATION_OF_LEUKOCYTE_MIGRATION	164	0.38860035	1.7279302	0	0.1055624	0.999
GOBP_POSITIVE_REGULATION_OF_SIGNALING_RECEPTOR_ACTIVITY	32	0.52055913	1.7189786	0	0.11064549	0.999
GOBP_DEFENSE_RESPONSE_TO_BACTERIUM	124	0.40100658	1.7133186	0	0.11368226	0.999

GOBP_POSITIVE_REGULATION_OF_PROTEIN_TYROSINE_KINASE_ACTIVITY	48	0.47914562	1.7119906	0	0.11437127	1
GOBP_CELL_CHEMOTAXIS	231	0.36126345	1.7023201	0	0.11828239	1
GOBP_REGULATION_OF_REACTIVE_OXYGEN_SPECIES_METABOLIC_PROCESS	149	0.38050663	1.6908894	0	0.12187077	1
GOBP_RESPONSE_TO_BACTERIUM	403	0.34294772	1.6908519	0	0.121041246	1
GOBP_LEUKOCYTE_MIGRATION	344	0.34419572	1.6839863	0	0.12616333	1
GOBP_POSITIVE_REGULATION_OF_INTERLEUKIN_6_PRODUCTION	69	0.42912984	1.6790985	0	0.12835379	1
GOBP_POSITIVE_REGULATION_OF_ERK1_AND_ERK2_CASCADE	141	0.37943792	1.6780508	0	0.12836133	1
GOBP_POSITIVE_REGULATION_OF_CELL_CELL_ADHESION	216	0.35448503	1.6722785	0	0.12925264	1
GOBP_NEGATIVE_REGULATION_OF_CYTOKINE_PRODUCTION	234	0.35056418	1.6498628	0	0.14171413	1
GOBP_TISSUE_REMODELING	126	0.38093486	1.634729	0	0.14963065	1
GOBP_REGULATION_OF_CELL_CELL_ADHESION	337	0.33567807	1.6311891	0	0.15025668	1
GOBP_POSITIVE_REGULATION_OF_RESPONSE_TO_EXTERNAL_STIMULUS	394	0.3299016	1.6265808	0	0.15143852	1
GOBP_CELLULAR_RESPONSE_TO_LIPID	422	0.32562777	1.6148168	0	0.15983064	1
GOBP_LIPID_LOCALIZATION	356	0.33206156	1.6066504	0	0.1662874	1
GOBP_POSITIVE_REGULATION_OF_DEFENSE_RESPONSE	281	0.33428472	1.6044168	0	0.16568688	1
GOBP_POSITIVE_REGULATION_OF_CYTOKINE_PRODUCTION	344	0.32697114	1.5919582	0	0.1737016	1
GOBP_REACTIVE_OXYGEN_SPECIES_METABOLIC_PROCESS	206	0.3502389	1.5916183	0	0.17330913	1
GOBP_POSITIVE_REGULATION_OF_CELL_ACTIVATION	274	0.3348376	1.5841839	0	0.17725125	1
GOBP_CELLULAR_PROCESS_INVOLVED_IN_REPRODUCTION_IN_MULTICELLULA	204	0.34360477	1.5795828	0	0.17983241	1
GOBP_REGULATION_OF_NUCLEAR_DIVISION	118	0.3683367	1.576123	0	0.18109098	1
GOBP_REGULATION_OF_CELL_ACTIVATION	456	0.3128791	1.5716729	0	0.18447194	1
GOBP_POSITIVE_REGULATION_OF_LYMPHOCYTE_DIFFERENTIATION	84	0.39204577	1.5685269	0	0.1857678	1
GOBP_MYELOID_LEUKOCYTE_MEDIATED_IMMUNITY	452	0.31484875	1.5615139	0	0.18991208	1
GOBP_TAXIS	469	0.30952162	1.5467883	0	0.20036829	1
GOBP_LEUKOCYTE_CELL_CELL_ADHESION	287	0.32542962	1.5394967	0	0.20409663	1
GOBP_MEIOTIC_CELL_CYCLE_PROCESS	128	0.35280392	1.5131655	0	0.2208527	1
GOBP_MULTICELLULAR_ORGANISMAL_HOMEOSTASIS	381	0.30192548	1.4886198	0	0.23168792	1
GOBP_RESPONSE_TO_TOXIC_SUBSTANCE	165	0.3345242	1.4879601	0	0.23204182	1
GOBP_SENSORY_PERCEPTION	293	0.30922642	1.4861643	0	0.23395117	1
GOBP_WOUND_HEALING	414	0.29964527	1.4820219	0	0.2382627	1
GOBP_REGULATION_OF_PROTEIN_SERINE_THREONINE_KINASE_ACTIVITY	408	0.29558516	1.4749733	0	0.24298175	1
GOBP_ANION_TRANSMEMBRANE_TRANSPORT	378	0.2999991	1.4677787	0	0.24633354	1
GOBP_POSITIVE_REGULATION_OF_MAPK_CASCADE	390	0.29462504	1.4609149	0	0.24850078	1
GOBP_RESPONSE_TO_INSULIN	233	0.31049103	1.4577286	0	0.24944885	1
GOBP_RESPONSE_TO_PEPTIDE	413	0.29420158	1.45691	0	0.24994168	1
GOBP_GLIOGENESIS	219	0.31028184	1.4458905	0	0.25579977	1
GOBP_POSITIVE_REGULATION_OF_LOCOMOTION	458	0.2844026	1.4285363	0	0.26711762	1
GOBP_REGULATION_OF_T_CELL_ACTIVATION	263	0.29838976	1.426474	0	0.26726264	1

GOBP_POSITIVE_REGULATION_OF_SECRETION	205	0.31116837	1.4262398	0	0.2670172	1
GOBP_POSITIVE_REGULATION_OF_PROTEIN_KINASE_ACTIVITY	424	0.28823414	1.4220378	0	0.26875305	1
GOBP_DEVELOPMENTAL_GROWTH	449	0.2818112	1.4158745	0	0.27514857	1
GOBP_RESPONSE_TO_PEPTIDE_HORMONE	339	0.28934994	1.4107841	0	0.2774158	1
GOBP_REGULATION_OF_VESICLE_MEDIATED_TRANSPORT	402	0.2857517	1.3932171	0	0.29083502	1
GOBP_REACTIVE_NITROGEN_SPECIES_METABOLIC_PROCESS	62	0.45692566	1.7338932	0.002325581	0.10487625	0.999
GOBP_CELL_JUNCTION_DISASSEMBLY	19	0.6348267	1.8107029	0.002375297	0.07426099	0.971
GOBP_COMPLEMENT_ACTIVATION	32	0.55195487	1.782293	0.002375297	0.08426918	0.985
GOBP_BONE_RESORPTION	49	0.46180084	1.6836913	0.002380953	0.12555255	1
GOBP_OOCYTE_MATURATION	18	0.6339974	1.8231547	0.002409639	0.07055148	0.958
GOBP_RESPONSE_TO_NICOTINE	22	0.6264463	1.8731664	0.002439024	0.060179185	0.858
GOBP_PROTEIN_CONTAINING_COMPLEX_REMODELING	19	0.6946758	1.9692795	0.002444988	0.03746961	0.495
GOBP_INORGANIC_ANION_TRANSMEMBRANE_TRANSPORT	60	0.44867176	1.6778549	0.002463054	0.1276236	1
GOBP_APOPTOTIC_CELL_CLEARANCE	39	0.5381548	1.8564727	0.002469136	0.06335574	0.901
GOBP_POSITIVE_REGULATION_OF_STEROL_TRANSPORT	24	0.60908484	1.889437	0.002475248	0.058414526	0.805
GOBP_REGULATION_OF_MACROPHAGE_MIGRATION	34	0.5325363	1.7761378	0.002487562	0.08697085	0.989
GOBP_LUNG_ALVEOLUS_DEVELOPMENT	32	0.5358155	1.7694569	0.0025	0.08898389	0.994
GOBP_RESPONSE_TO_CHEMOKINE	68	0.4548171	1.7351528	0.002506266	0.10479331	0.999
GOBP_NEGATIVE_REGULATION_OF_ENDOTHELIAL_CELL_PROLIFERATION	38	0.4804347	1.6542069	0.002518892	0.13914351	1
GOBP_ANTIMICROBIAL_HUMORAL_RESPONSE	46	0.52343667	1.8741313	0.002531646	0.060841516	0.857
GOBP_REGULATION_OF_HUMORAL_IMMUNE_RESPONSE	42	0.5120623	1.7886517	0.002544529	0.085063465	0.982
GOBP_POSITIVE_REGULATION_OF_REPRODUCTIVE_PROCESS	48	0.48244122	1.7509493	0.002544529	0.09894315	0.997
GOBP_REGULATION_OF_OSTEOCLAST_DIFFERENTIATION	48	0.4948901	1.792511	0.002557545	0.082900904	0.978
GOBP_REGULATION_OF_ANOIKIS	20	0.6204975	1.8480917	0.002564103	0.0656997	0.916
GOBP_POSITIVE_REGULATION_OF_MITOTIC_NUCLEAR_DIVISION	35	0.54215854	1.7739526	0.002570694	0.08690034	0.991
GOBP_STEROL_TRANSPORT	85	0.4155536	1.6613889	0.00257732	0.13526404	1
GOBP_MACROPHAGE_MIGRATION	42	0.52169204	1.8349603	0.002583979	0.065857425	0.935
GOBP_FATTY_ACID_TRANSMEMBRANE_TRANSPORT	40	0.49322477	1.7287283	0.002583979	0.107529484	0.999
GOBP_AMINO_ACID_TRANSMEMBRANE_TRANSPORT	65	0.44055128	1.6806811	0.002604167	0.12762453	1
GOBP_ORGANIC_ACID_TRANSMEMBRANE_TRANSPORT	114	0.3754804	1.570754	0.002617801	0.18499589	1
GOBP_PHOSPHATIDYLCHOLINE_METABOLIC_PROCESS	61	0.4429634	1.6673448	0.002624672	0.13162449	1
GOBP_GLIAL_CELL_PROLIFERATION	38	0.5418063	1.8428729	0.002631579	0.0650879	0.927
GOBP_REGULATION_OF_BONE_REMODELING	39	0.57027495	1.9447044	0.002659574	0.04393421	0.586
GOBP_BONE_REMODELING	65	0.42244816	1.6180537	0.002666667	0.15770516	1
GOBP_DETOXIFICATION	87	0.40916768	1.6096151	0.002666667	0.16362932	1
GOBP_NEGATIVE_REGULATION_OF_RESPONSE_TO_WOUNDING	64	0.42476365	1.6153094	0.002673797	0.16004235	1
GOBP_REGULATION_OF_NIK_NF_KAPPAB_SIGNALING	86	0.42095777	1.6972322	0.002702703	0.11940844	1
GOBP_POSITIVE_REGULATION_OF_CHEMOTAXIS	110	0.38477153	1.6332135	0.002785515	0.1496374	1

GOBP_ORGAN_GROWTH	117	0.37949845	1.630572	0.002785515	0.15011348	1
GOBP_REGULATION_OF_CYCLIN_DEPENDENT_PROTEIN_KINASE_ACTIVITY	89	0.36891395	1.5080333	0.002793296	0.222183	1
GOBP_REGULATION_OF_CHEMOTAXIS	176	0.33362937	1.5172645	0.002801121	0.21797705	1
GOBP_TOLL_LIKE_RECEPTOR_SIGNALING_PATHWAY	138	0.33904704	1.4743819	0.002816901	0.24239105	1
GOBP_REGULATION_OF_REACTIVE_OXYGEN_SPECIES_BIOSYNTHETIC_PROCESS	71	0.44096142	1.7396696	0.002906977	0.10333735	0.999
GOBP_POSITIVE_REGULATION_OF_HEMOPOIESIS	126	0.37844673	1.6362063	0.002915452	0.14874852	1
GOBP_REGULATION_OF_MAP_KINASE_ACTIVITY	250	0.29788762	1.4025621	0.002932551	0.28342456	1
GOBP_REGULATION_OF_RESPONSE_TO_WOUNDING	121	0.35245305	1.506752	0.002976191	0.22032402	1
GOBP_POSITIVE_REGULATION_OF_IMMUNE_EFFECTOR_PROCESS	167	0.32894906	1.4800218	0.003012048	0.2392929	1
GOBP_NEGATIVE_REGULATION_OF_RESPONSE_TO_EXTERNAL_STIMULUS	272	0.3007351	1.4399426	0.003076923	0.2607385	1
GOBP_LEUKOCYTE_CHEMOTAXIS	171	0.38666117	1.7478673	0.00308642	0.09905817	0.997
GOBP_POSITIVE_REGULATION_OF_PROTEIN_SERINE_THREONINE_KINASE_ACT	254	0.31636843	1.4920149	0.003095975	0.23356691	1
GOBP_PHAGOCYTOSIS	228	0.3344991	1.5577139	0.003134796	0.19196446	1
GOBP_MAINTENANCE_OF_LOCATION	259	0.30788368	1.463371	0.003154574	0.24757554	1
GOBP_REGULATION_OF_VASCULATURE_DEVELOPMENT	232	0.30722612	1.4375677	0.003194888	0.26175883	1
GOBP_POSITIVE_REGULATION_OF_CELL_ADHESION	340	0.2809048	1.374318	0.003333333	0.3039516	1
GOBP_POSITIVE_REGULATION_OF_IMMUNE_RESPONSE	495	0.25376353	1.2856323	0.004016064	0.3872321	1
GOBP_RESPONSE_TO_ISCHEMIA	42	0.5057198	1.7770532	0.004750594	0.08717787	0.989
GOBP_OOCYTE_DIFFERENTIATION	31	0.5647895	1.8499744	0.004784689	0.06554252	0.914
GOBP_REGULATION_OF_MYELOID_LEUKOCYTE_MEDIATED_IMMUNITY	44	0.48460588	1.7075646	0.004784689	0.11488579	1
GOBP_RESPONSE_TO_FUNGUS	25	0.5531681	1.7021644	0.004796163	0.116648555	1
GOBP_LIPID_IMPORT_INTO_CELL	28	0.59610844	1.8725711	0.004819277	0.059300445	0.859
GOBP_REGULATION_OF_GRANULOCYTE_CHEMOTAXIS	38	0.5038997	1.7136985	0.004878049	0.11416299	0.999
GOBP_POSITIVE_REGULATION_OF_MACROPHAGE_ACTIVATION	22	0.62394005	1.8950655	0.004914005	0.056302123	0.79
GOBP_POSITIVE_REGULATION_OF_STEROID_METABOLIC_PROCESS	19	0.62552917	1.8252655	0.004926108	0.070054434	0.954
GOBP_POSITIVE_REGULATION_OF_CYCLIN_DEPENDENT_PROTEIN_KINASE_ACT	30	0.55070007	1.7873818	0.004987531	0.08302771	0.982
GOBP_PHOSPHATIDYLCHOLINE_BIOSYNTHETIC_PROCESS	30	0.58932036	1.9103565	0.005012531	0.05069745	0.721
GOBP_REGULATION_OF_GLUCOSE_TRANSMEMBRANE_TRANSPORT	58	0.4437931	1.6696054	0.005012531	0.13160521	1
GOBP_REGULATION_OF_MACROPHAGE_CHEMOTAXIS	23	0.5857195	1.7790568	0.005025126	0.0863091	0.988
GOBP_POSITIVE_REGULATION_OF_VASCULAR_ENDOTHELIAL_GROWTH_FACTC	23	0.61664325	1.9220353	0.005037783	0.0500844	0.667
GOBP_NEGATIVE_REGULATION_OF_LIPID_BIOSYNTHETIC_PROCESS	40	0.4673323	1.643363	0.005089059	0.1423167	1
GOBP_NEUROINFLAMMATORY_RESPONSE	35	0.54496795	1.8769737	0.005194805	0.06017676	0.847
GOBP_POSITIVE_REGULATION_OF_LEUKOCYTE_DEGRANULATION	17	0.6208739	1.7884123	0.005208334	0.084234275	0.982
GOBP_INTERLEUKIN_8_PRODUCTION	73	0.40332642	1.5681372	0.005263158	0.18551262	1
GOBP_REGULATION_OF_NITRIC_OXIDE_METABOLIC_PROCESS	50	0.47175512	1.7117095	0.005291005	0.11285754	1
GOBP_REGULATION_OF_BIOMINERALIZATION	65	0.42781183	1.6455745	0.005405406	0.14327872	1
GOBP_ACTIVATION_OF_MAPK_ACTIVITY	120	0.36628142	1.5513681	0.005405406	0.19591089	1
GOBP_REGULATION_OF_CALCIUM_MEDIATED_SIGNALING	50	0.44548455	1.6315782	0.005509642	0.15065774	1

GOBP_NEGATIVE_REGULATION_OF_NEURON_DEATH	137	0.33829746	1.479381	0.005509642	0.23954831	1
GOBP_MEMBRANE_PROTEIN_PROTEOLYSIS	55	0.42824218	1.6058515	0.005524862	0.16560224	1
GOBP_REGULATION_OF_NERVOUS_SYSTEM_PROCESS	87	0.384619	1.5860901	0.005633803	0.17561002	1
GOBP_MONONUCLEAR_CELL_MIGRATION	142	0.3758346	1.6640614	0.005649718	0.13338487	1
GOBP_POSITIVE_REGULATION_OF_TUMOR_NECROSIS_FACTOR_SUPERFAMILY_	75	0.4204329	1.6436318	0.005730659	0.14277735	1
GOBP_ENDOTHELIAL_CELL_PROLIFERATION	129	0.36060023	1.5888034	0.005730659	0.17343654	1
GOBP_POSITIVE_REGULATION_OF_PEPTIDYL_TYROSINE_PHOSPHORYLATION	139	0.35194474	1.5060722	0.005763689	0.22065318	1
GOBP_POSITIVE_REGULATION_OF_LEUKOCYTE_CELL_CELL_ADHESION	187	0.33285037	1.5092751	0.005813954	0.22271083	1
GOBP_REGULATION_OF_HEMOPOIESIS	329	0.28807858	1.3888378	0.006430868	0.2929336	1
GOBP_REGULATION_OF_INFLAMMATORY_RESPONSE	258	0.32112998	1.5319859	0.006622517	0.21078531	1
GOBP_CELLULAR_RESPONSE_TO_PEPTIDE	310	0.28641146	1.3856363	0.006644518	0.29356098	1
GOBP_REGULATION_OF_IMMUNE_EFFECTOR_PROCESS	307	0.28524187	1.3789424	0.006825938	0.30127743	1
GOBP_T_CELL_ACTIVATION	384	0.26868042	1.3258061	0.006993007	0.35320905	1
GOBP_FEAR_RESPONSE	19	0.65534216	1.940624	0.007159905	0.044854827	0.603
GOBP_NEGATIVE_REGULATION_OF_TISSUE_REMODELING	15	0.66850275	1.8721851	0.007194245	0.05856929	0.861
GOBP_NEGATIVE_REGULATION_OF_OXIDATIVE_STRESS_INDUCED_NEURON_D	15	0.6495158	1.7738836	0.007317073	0.08609564	0.991
GOBP_RESPONSE_TO_MINERALOCORTICOID	21	0.5732826	1.7038823	0.007334963	0.11766539	1
GOBP_REGULATION_OF_STEROL_TRANSPORT	41	0.48499978	1.7210643	0.007352941	0.109632105	0.999
GOBP_RETINA_HOMEOSTASIS	43	0.4845456	1.708213	0.007389163	0.11507728	1
GOBP_POSITIVE_REGULATION_OF_MEMBRANE_PROTEIN_ECTODOMAIN_PROT	15	0.6596427	1.7745688	0.007537689	0.08733851	0.991
GOBP_TOLL_LIKE_RECEPTOR_4_SIGNALING_PATHWAY	37	0.51952404	1.7578459	0.007594937	0.09487962	0.994
GOBP_POSITIVE_REGULATION_OF_VASCULAR_ASSOCIATED_SMOOTH_MUSCLE	29	0.52071196	1.6949664	0.007614213	0.11886496	1
GOBP_ENDOTHELIAL_CELL_APOPTOTIC_PROCESS	44	0.4545909	1.6197232	0.007692308	0.15659769	1
GOBP_NEGATIVE_REGULATION_OF_LEUKOCYTE_PROLIFERATION	66	0.4028188	1.5575606	0.007792208	0.19139147	1
GOBP_POSITIVE_REGULATION_OF_STRESS_ACTIVATED_PROTEIN_KINASE_SIGN	98	0.36226553	1.4963299	0.007915568	0.23119308	1
GOBP_POSITIVE_REGULATION_OF_REACTIVE_OXYGEN_SPECIES_BIOSYNTHETIC	38	0.49381715	1.6989425	0.007978723	0.1187944	1
GOBP_EPITHELIAL_CELL_APOPTOTIC_PROCESS	84	0.39295658	1.5765738	0.008	0.18132727	1
GOBP_PEPTIDYL_CYSTEINE_MODIFICATION	42	0.49829915	1.7389756	0.00802139	0.1029558	0.999
GOBP_TEMPERATURE_HOMEOSTASIS	133	0.3511494	1.4936432	0.008042895	0.23272519	1
GOBP_REGULATION_OF_MONOOXYGENASE_ACTIVITY	48	0.44264373	1.5915192	0.008086253	0.17185201	1
GOBP_ZYMOGEN_ACTIVATION	40	0.48272014	1.6846813	0.008196721	0.12643766	1
GOBP_L_ALPHA_AMINO_ACID_TRANSMEMBRANE_TRANSPORT	44	0.46812576	1.6554121	0.008196721	0.13871716	1
GOBP_METAL_ION_HOMEOSTASIS	453	0.2510517	1.2615561	0.008264462	0.40902218	1
GOBP_NEGATIVE_REGULATION_OF_LIPID_METABOLIC_PROCESS	69	0.41519794	1.6061229	0.008379889	0.1660523	1
GOBP_SUBSTRATE_ADHESION_DEPENDENT_CELL_SPREADING	93	0.3697598	1.508293	0.008426966	0.2233602	1
GOBP_POSITIVE_REGULATION_OF_EPITHELIAL_CELL_PROLIFERATION	146	0.32659703	1.4333768	0.008426966	0.26318842	1
GOBP_REGULATION_OF_CELL_SUBSTRATE_ADHESION	176	0.3213199	1.4460073	0.008498584	0.25632325	1
GOBP_POSITIVE_REGULATION_OF_VASCULATURE_DEVELOPMENT	130	0.3460236	1.4918073	0.008571428	0.23316252	1

GOBP_REGULATION_OF_LEUKOCYTE_DIFFERENTIATION	228	0.30009732	1.3813529	0.008849558	0.2990635	1
GOBP_POSITIVE_REGULATION_OF_SMOOTH_MUSCLE_CONTRACTION	15	0.64384425	1.7617446	0.009280742	0.09240152	0.994
GOBP_REGULATION_OF_MAST_CELL_ACTIVATION	32	0.49113375	1.6678534	0.009638554	0.13191065	1
GOBP_REGULATION_OF_MACROPHAGE_DERIVED_FOAM_CELL_DIFFERENTIATI	19	0.6061507	1.7525008	0.009779952	0.09826745	0.995
GOBP_REGULATION_OF_NEUTROPHIL_CHEMOTAXIS	26	0.54183674	1.6961623	0.01	0.11948304	1
GOBP_REGULATION_OF_TRANSMEMBRANE_TRANSPORT	374	0.2723477	1.3333815	0.01010101	0.34871244	1
GOBP_POSITIVE_REGULATION_OF_NUCLEAR_DIVISION	45	0.5131179	1.8165097	0.010204081	0.07391592	0.966
GOBP_POSITIVE_REGULATION_OF_LIPID_TRANSPORT	63	0.4117065	1.6043499	0.010230179	0.16495794	1
GOBP_REGULATION_OF_PLATELET_ACTIVATION	30	0.53001213	1.7323066	0.010282776	0.1053674	0.999
GOBP_REGULATION_OF_EXTRINSIC_APOPTOTIC_SIGNALING_PATHWAY_IN_AB	32	0.51227874	1.6950687	0.010282776	0.119646296	1
GOBP_HORMONE_METABOLIC_PROCESS	114	0.33379483	1.4257572	0.010526316	0.26712477	1
GOBP_OSSIFICATION	301	0.27649924	1.3315026	0.01056338	0.35056	1
GOBP_REGULATION_OF_REGULATED_SECRETORY_PATHWAY	95	0.36071736	1.5074137	0.01058201	0.22236076	1
GOBP_NEGATIVE_REGULATION_OF_NF_KAPPAB_TRANSCRIPTION_FACTOR_ACT	71	0.3919931	1.5125463	0.010638298	0.22104146	1
GOBP_NEGATIVE_REGULATION_OF_CELL_CELL_ADHESION	143	0.3243226	1.427016	0.010638298	0.26832736	1
GOBP_ADENYLATE_CYCLASE_MODULATING_G_PROTEIN_COUPLED_RECEPTOR_	127	0.32784224	1.4321107	0.010695187	0.2645301	1
GOBP_ACUTE_INFLAMMATORY_RESPONSE	67	0.41475508	1.5804967	0.010781671	0.18026693	1
GOBP_CELLULAR_RESPONSE_TO_TOXIC_SUBSTANCE	82	0.3639873	1.4805785	0.010810811	0.23920624	1
GOBP_CIRCULATORY_SYSTEM_PROCESS	413	0.255753	1.2800558	0.010869565	0.39246637	1
GOBP_OSTEOCLAST_DIFFERENTIATION	75	0.3916842	1.5414318	0.010928961	0.20371854	1
GOBP_POSITIVE_REGULATION_OF_ANION_TRANSPORT	373	0.26243785	1.2971817	0.010989011	0.37609506	1
GOBP_REGULATION_OF_MITOTIC_NUCLEAR_DIVISION	99	0.35021937	1.4376717	0.011019284	0.2622497	1
GOBP_SMOOTH_MUSCLE_CELL_PROLIFERATION	113	0.35795978	1.5070993	0.011080332	0.22127827	1
GOBP_POSITIVE_REGULATION_OF_SMALL_MOLECULE_METABOLIC_PROCESS	99	0.36528787	1.5113013	0.011695907	0.22118042	1
GOBP_PROTEIN_PROCESSING	164	0.32205927	1.4457905	0.011695907	0.25529778	1
GOBP_REGULATION_OF_PEPTIDYL_TYROSINE_PHOSPHORYLATION	199	0.31400394	1.4305085	0.011764706	0.2658502	1
GOBP_LEUKOCYTE_MIGRATION_INVOLVED_IN_INFLAMMATORY_RESPONSE	15	0.63447857	1.7146002	0.011792453	0.114142135	0.999
GOBP_IMPORT_INTO_CELL	151	0.3148921	1.3982687	0.011869436	0.2875318	1
GOBP_REGULATION_OF_OXIDATIVE_STRESS_INDUCED_NEURON_DEATH	21	0.5611327	1.7117716	0.011990408	0.11370769	1
GOBP_ORGANIC_ACID_TRANSPORT	203	0.2911533	1.3322849	0.012012012	0.3502386	1
GOBP_NEGATIVE_REGULATION_OF_LIPID_CATABOLIC_PROCESS	16	0.64387316	1.7864416	0.012048192	0.08283513	0.985
GOBP_MEMBRANE_PROTEIN_ECTODOMAIN_PROTEOLYSIS	39	0.47926745	1.6461227	0.012195122	0.14435925	1
GOBP_POTASSIUM_ION_TRANSPORT	133	0.34859496	1.5272964	0.012307692	0.21431185	1
GOBP_MYOTUBE_CELL_DEVELOPMENT	22	0.5526524	1.6292902	0.012376238	0.1500577	1
GOBP_NEGATIVE_REGULATION_OF_MUSCLE_CELL_APOPTOTIC_PROCESS	27	0.4992926	1.5953761	0.0125	0.1704636	1
GOBP_POSITIVE_REGULATION_OF_BONE_MINERALIZATION	28	0.5283764	1.6531644	0.012531328	0.13952285	1
GOBP_MONOCYTE_CHEMOTAXIS	39	0.4834688	1.6506824	0.012594459	0.14165054	1
GOBP_REGULATION_OF_CHOLESTEROL_EFFLUX	24	0.5661572	1.7380271	0.012919896	0.10296441	0.999

GOBP_CELLULAR_RESPONSE_TO_PEPTIDE_HORMONE_STIMULUS	253	0.30113634	1.4084607	0.012987013	0.27805862	1
GOBP_POSITIVE_REGULATION_OF_ENDOTHELIAL_CELL_APOPTOTIC_PROCESS	15	0.63918376	1.7402861	0.01305483	0.10466282	0.999
GOBP_REGULATION_OF_EPITHELIAL_TO_MESENCHYMAL_TRANSITION	84	0.36142054	1.4457461	0.013089005	0.2546847	1
GOBP_REGULATION_OF_SENSORY_PERCEPTION	20	0.59628665	1.7230166	0.013192612	0.10863714	0.999
GOBP_FEEDING_BEHAVIOR	39	0.46559623	1.6029547	0.013477089	0.16490579	1
GOBP_IMPORT_ACROSS_PLASMA_MEMBRANE	107	0.33979845	1.4249538	0.013477089	0.2671879	1
GOBP_POSITIVE_REGULATION_OF_SMOOTH_MUSCLE_CELL_PROLIFERATION	72	0.40308726	1.6023651	0.013586956	0.16484381	1
GOBP_REGULATION_OF_EPITHELIAL_CELL_APOPTOTIC_PROCESS	63	0.39183977	1.4906535	0.013623978	0.23202151	1
GOBP_NEGATIVE_REGULATION_OF_OSSIFICATION	25	0.50486344	1.6048901	0.013921114	0.16578043	1
GOBP_INTERLEUKIN_12_PRODUCTION	51	0.42676893	1.5672424	0.013927577	0.18523493	1
GOBP_REGULATION_OF_HORMONE_LEVELS	312	0.27270377	1.3251156	0.013986014	0.3520695	1
GOBP_POSITIVE_REGULATION_OF_PEPTIDE_SECRETION	107	0.34855455	1.4547989	0.014084507	0.2505229	1
GOBP_PROTEIN_NITROSYLATION	15	0.62324095	1.6587385	0.014184397	0.1357773	1
GOBP_SENSORY_PERCEPTION_OF_SMELL	17	0.5936982	1.6724135	0.014634146	0.1307972	1
GOBP_PATTERN_RECOGNITION_RECEPTOR_SIGNALING_PATHWAY	185	0.30856076	1.4264746	0.014705882	0.26790664	1
GOBP_NEGATIVE_REGULATION_OF_INTERLEUKIN_6_PRODUCTION	40	0.4745933	1.6471272	0.014778325	0.14388126	1
GOBP_FOAM_CELL_DIFFERENTIATION	25	0.52969694	1.6446856	0.014888338	0.14262472	1
GOBP_POSITIVE_REGULATION_OF_OSTEOCLAST_DIFFERENTIATION	20	0.57774085	1.7287197	0.014962593	0.10663143	0.999
GOBP_NEGATIVE_REGULATION_OF_OSTEOCLAST_DIFFERENTIATION	21	0.5812969	1.7262357	0.015	0.106514774	0.999
GOBP_REGULATION_OF_LEUKOCYTE_MEDIATED_IMMUNITY	164	0.3225913	1.458855	0.015060241	0.25040928	1
GOBP_REGULATION_OF_SECRETION	428	0.24926366	1.2492322	0.01532567	0.42411035	1
GOBP_NEGATIVE_REGULATION_OF_EXTRINSIC_APOPTOTIC_SIGNALING_PATHW	85	0.38640592	1.5422671	0.015337423	0.20410934	1
GOBP_POSITIVE_REGULATION_OF_GRANULOCYTE_CHEMOTAXIS	19	0.56297785	1.6458613	0.015424165	0.14382079	1
GOBP_STEROL_HOMEOSTASIS	58	0.40463874	1.525694	0.015424165	0.21502411	1
GOBP_REGULATION_OF_GLIAL_CELL_MIGRATION	16	0.63799274	1.7624476	0.015544041	0.09282451	0.994
GOBP_ENDOCRINE_PROCESS	47	0.43692347	1.5732384	0.015544041	0.18331341	1
GOBP_MACROPHAGE_ACTIVATION	81	0.35326102	1.4279175	0.015544041	0.26747897	1
GOBP_L_AMINO_ACID_TRANSPORT	48	0.44928923	1.612784	0.015706806	0.16136892	1
GOBP_OVULATION_CYCLE	44	0.44781184	1.6017925	0.015706806	0.16395378	1
GOBP_HETEROTYPIC_CELL_CELL_ADHESION	43	0.43924034	1.5279161	0.015748031	0.2141171	1
GOBP_REGULATION_OF_PROTEIN_TYROSINE_KINASE_ACTIVITY	80	0.36573333	1.4672756	0.015873017	0.24573144	1
GOBP_REACTIVE_OXYGEN_SPECIES_BIOSYNTHETIC_PROCESS	86	0.37753555	1.5197067	0.01591512	0.21609247	1
GOBP_REGULATION_OF_SYSTEMIC_ARTERIAL_BLOOD_PRESSURE_MEDIATED_E	28	0.522835	1.6604383	0.016203703	0.13472903	1
GOBP_REGULATION_OF_LIPID_BIOSYNTHETIC_PROCESS	154	0.31902218	1.4151542	0.016713092	0.27445737	1
GOBP_EXTERNAL_ENCAPSULATING_STRUCTURE_ORGANIZATION	309	0.2638814	1.2799995	0.016891891	0.39200503	1
GOBP_LIPID_TRANSLOCATION	36	0.45440337	1.569418	0.01703163	0.1859569	1
GOBP_POSITIVE_REGULATION_OF_MONONUCLEAR_CELL_MIGRATION	50	0.4037019	1.4854333	0.017114915	0.23431353	1
GOBP_LAMELLIPODIUM_MORPHOGENESIS	15	0.61598325	1.6765971	0.017412934	0.12811816	1

GOBP_NEGATIVE_REGULATION_OF_PRODUCTION_OF_MOLECULAR_MEDIATOI	26	0.4974517	1.5781171	0.01758794	0.18102346	1
GOBP_NIK_NF_KAPPAB_SIGNALING	156	0.30587348	1.3683738	0.017804155	0.3093236	1
GOBP_REGULATION_OF_DEVELOPMENTAL_GROWTH	229	0.27601817	1.2788026	0.017804155	0.38930404	1
GOBP_NEGATIVE_REGULATION_OF_CELL_ADHESION	225	0.28296712	1.3176531	0.017964073	0.3593764	1
GOBP_REGULATION_OF_SYSTEM_PROCESS	371	0.2502261	1.234354	0.018181818	0.43407494	1
GOBP_ORGANIC_HYDROXY_COMPOUND_TRANSPORT	165	0.31098032	1.3941288	0.018461538	0.29118237	1
GOBP_NEGATIVE_REGULATION_OF_WOUND_HEALING	50	0.41632858	1.5206227	0.018469658	0.21632817	1
GOBP_LUNG_CELL_DIFFERENTIATION	16	0.6134567	1.7021854	0.018518519	0.1174776	1
GOBP_NEGATIVE_REGULATION_OF_SIGNAL_TRANSDUCTION_IN_ABSENCE_OF_	25	0.5256189	1.612253	0.01861702	0.16119768	1
GOBP_POSITIVE_REGULATION_OF_TRANSMEMBRANE_RECEPTOR_PROTEIN_SE	81	0.37151593	1.4674091	0.01897019	0.24616621	1
GOBP_TISSUE_HOMEOSTASIS	188	0.30783463	1.4074796	0.01910828	0.27926946	1
GOBP_GERM_CELL_DEVELOPMENT	146	0.34404382	1.502428	0.019607844	0.22370139	1
GOBP_CELLULAR_OXIDANT_DETOXIFICATION	68	0.38672197	1.4890621	0.019830028	0.23172271	1
GOBP_STRIATED_MUSCLE_CELL_DIFFERENTIATION	186	0.30005214	1.3888019	0.02	0.29239142	1
GOBP_NEGATIVE_REGULATION_OF_CELL_DEVELOPMENT	120	0.31283626	1.3449366	0.020408163	0.333027	1
GOBP_LYMPH_NODE_DEVELOPMENT	15	0.59810835	1.6608306	0.020460358	0.13507265	1
GOBP_POSITIVE_REGULATION_OF_NITRIC_OXIDE_METABOLIC_PROCESS	31	0.5193199	1.6684885	0.020881671	0.13215137	1
GOBP_POSITIVE_REGULATION_OF_ACTIVATED_T_CELL_PROLIFERATION	19	0.559794	1.6393292	0.021028038	0.14594564	1
GOBP_PROTEIN_KINASE_B_SIGNALING	198	0.28535315	1.3278601	0.021148037	0.35153547	1
GOBP_SEXUAL_REPRODUCTION	489	0.24986902	1.2541848	0.021201413	0.4179654	1
GOBP_NEGATIVE_REGULATION_OF_CELL_MATRIX_ADHESION	32	0.490037	1.6332201	0.021276595	0.15045089	1
GOBP_REGULATION_OF_CELL_MATRIX_ADHESION	104	0.34888297	1.449956	0.021276595	0.2543484	1
GOBP_EXTRACELLULAR_MATRIX_DISASSEMBLY	58	0.4108185	1.5360261	0.021563342	0.20735615	1
GOBP_POSITIVE_REGULATION_OF_MAP_KINASE_ACTIVITY	185	0.30602288	1.387517	0.021671826	0.29215777	1
GOBP_NEGATIVE_REGULATION_OF_DEVELOPMENTAL_GROWTH	82	0.35909775	1.4337058	0.021686748	0.26468414	1
GOBP_REGULATION_OF_NITRIC_OXIDE_SYNTHASE_ACTIVITY	42	0.47620955	1.6453344	0.02200489	0.14270593	1
GOBP_NEGATIVE_REGULATION_OF_B_CELL_ACTIVATION	31	0.4896069	1.626836	0.022038568	0.15190846	1
GOBP_INTERFERON_GAMMA_PRODUCTION	84	0.35432675	1.4525347	0.022038568	0.25283307	1
GOBP_MUSCLE_CELL_PROLIFERATION	149	0.32299787	1.4018078	0.022099448	0.28407305	1
GOBP_ANTIMICROBIAL_HUMORAL_IMMUNE_RESPONSE_MEDIATED_BY_ANTII	23	0.52847123	1.6019242	0.022113021	0.16461502	1
GOBP_ANOIKIS	30	0.48591632	1.5757611	0.022167487	0.18081807	1
GOBP_NEGATIVE_REGULATION_OF_T_CELL_PROLIFERATION	49	0.41528118	1.503557	0.022613065	0.22272949	1
GOBP_REGULATION_OF_VASCULAR_ASSOCIATED_SMOOTH_MUSCLE_CELL_PR	47	0.43821725	1.5614774	0.022727273	0.18917294	1
GOBP_POSITIVE_REGULATION_OF_ENDOTHELIAL_CELL_PROLIFERATION	82	0.34700873	1.3972626	0.022900764	0.2880696	1
GOBP_RESPONSE_TO_CORTICOSTEROID	104	0.3405125	1.416081	0.022922637	0.27539468	1
GOBP_MICROGLIAL_CELL_ACTIVATION	38	0.4471882	1.517815	0.023017902	0.21790491	1
GOBP_NEGATIVE_REGULATION_OF_MYELOID_LEUKOCYTE_DIFFERENTIATION	36	0.46478775	1.5805283	0.023316063	0.18102859	1
GOBP_REGULATION_OF_SIGNALING_RECEPTOR_ACTIVITY	114	0.354324	1.4958979	0.023391813	0.2310415	1

GOBP_EPIBOLY	32	0.47890678	1.5799347	0.0234375	0.18022795	1
GOBP_CARBOHYDRATE_TRANSMEMBRANE_TRANSPORT	86	0.34387666	1.3876479	0.0234375	0.29253536	1
GOBP_SIGNAL_TRANSDUCTION_IN_ABSENCE_OF_LIGAND	53	0.41302657	1.519863	0.02356021	0.21664503	1
GOBP_POSITIVE_REGULATION_OF_MUSCLE_CONTRACTION	24	0.51354396	1.5579271	0.023684211	0.19242772	1
GOBP_ODONTOGENESIS	78	0.36960843	1.456906	0.023746701	0.24927479	1
GOBP_REGULATION_OF_BLOOD_PRESSURE	112	0.33796492	1.4106846	0.023746701	0.2769611	1
GOBP_BONE_GROWTH	20	0.58082545	1.6762178	0.024330901	0.12770931	1
GOBP_COBALAMIN_METABOLIC_PROCESS	15	0.6030717	1.6202677	0.024330901	0.15685387	1
GOBP_SENSORY_PERCEPTION_OF_LIGHT_STIMULUS	113	0.3504402	1.4710923	0.024523161	0.2454651	1
GOBP_DIVALENT_INORGANIC_CATION_HOMEOSTASIS	343	0.26139128	1.28585	0.024734983	0.38734868	1
GOBP_NEGATIVE_REGULATION_OF_INTERLEUKIN_12_PRODUCTION	16	0.6023327	1.6723171	0.024830699	0.13005069	1
GOBP_PHOSPHOLIPID_TRANSPORT	69	0.37210366	1.4445355	0.025062656	0.2558372	1
GOBP_BONE_DEVELOPMENT	147	0.31599644	1.3839945	0.025280898	0.29519913	1
GOBP_REGULATION_OF_MYELOID_LEUKOCYTE_DIFFERENTIATION	91	0.34166554	1.4087107	0.025575448	0.27829129	1
GOBP_ODONTOGENESIS_OF_DENTIN_CONTAINING_TOOTH	50	0.41796505	1.5223565	0.025773196	0.21690738	1
GOBP_RESPONSE_TO_AMYLOID_BETA	43	0.4161464	1.4911466	0.025773196	0.23200649	1
GOBP_REGULATION_OF_NUCLEOTIDE_BIOSYNTHETIC_PROCESS	28	0.4955528	1.5915343	0.025882352	0.17263632	1
GOBP_DIENCEPHALON_DEVELOPMENT	34	0.46193895	1.5269142	0.026004728	0.21405411	1
GOBP_SENSORY_PERCEPTION_OF_PAIN	58	0.3883508	1.4627697	0.026246719	0.2470283	1
GOBP_GAMETE_GENERATION	411	0.2476645	1.2341682	0.026515152	0.43389007	1
GOBP_OSTEOBLAST_DIFFERENTIATION	167	0.3008219	1.3457035	0.026627218	0.33284712	1
GOBP_REGULATION_OF_RUFFLE_ASSEMBLY	28	0.49172872	1.5672069	0.027173912	0.18453	1
GOBP_NEGATIVE_REGULATION_OF_NEURON_PROJECTION_DEVELOPMENT	106	0.33104846	1.3991164	0.027173912	0.2880173	1
GOBP_CARBOHYDRATE_TRANSPORT	114	0.3375519	1.4175277	0.027472528	0.27360103	1
GOBP_REGULATION_OF_ADAPTIVE_IMMUNE_RESPONSE	137	0.31019253	1.3559575	0.027472528	0.323215	1
GOBP_DEFENSE_RESPONSE_TO_GRAM_NEGATIVE_BACTERIUM	33	0.45747003	1.5400527	0.027522936	0.20406191	1
GOBP_SENSORY_PERCEPTION_OF_CHEMICAL_STIMULUS	41	0.45217678	1.565743	0.027707808	0.18577212	1
GOBP_REGULATION_OF_MICROGLIAL_CELL_ACTIVATION	18	0.54582864	1.5429721	0.027906977	0.20387429	1
GOBP_REGULATION_OF_COMPLEMENT_ACTIVATION	28	0.50702065	1.5900122	0.027918782	0.17271672	1
GOBP_REGULATION_OF_MULTICELLULAR_ORGANISM_GROWTH	46	0.43206576	1.5499295	0.027918782	0.19718845	1
GOBP_RHYTHMIC_PROCESS	222	0.2719653	1.275889	0.027950311	0.3936356	1
GOBP_MYELOID_DENDRITIC_CELL_ACTIVATION	24	0.54855806	1.6646781	0.028132992	0.13360703	1
GOBP_MORPHOGENESIS_OF_A_BRANCHING_STRUCTURE	130	0.31090173	1.3661091	0.028213166	0.31018412	1
GOBP_AMINO_ACID_IMPORT	35	0.48836574	1.6209402	0.028277636	0.1569793	1
GOBP_POSITIVE_REGULATION_OF_PROTEIN_MATURATION	21	0.52976334	1.5693166	0.028277636	0.18538463	1
GOBP_CELL_SUBSTRATE_ADHESION	286	0.26755854	1.2835752	0.028571429	0.38818038	1
GOBP_POSITIVE_REGULATION_OF_BIOMINERALIZATION	32	0.4690723	1.5311636	0.028645834	0.21103361	1
GOBP_HEART_GROWTH	67	0.37428743	1.4523736	0.02917772	0.2524176	1

GOBP_REGULATION_OF_SYSTEMIC_ARTERIAL_BLOOD_PRESSURE_BY_HORMON	21	0.55347115	1.6301877	0.029776676	0.14973699	1
GOBP_GLUCOSE_METABOLIC_PROCESS	171	0.2916619	1.3156586	0.029810298	0.36087975	1
GOBP_RESPONSE_TO_TUMOR_NECROSIS_FACTOR	244	0.27353773	1.2890483	0.029900333	0.38541082	1
GOBP_ACTIVATION_OF_PROTEIN_KINASE_ACTIVITY	261	0.27118874	1.288631	0.029900333	0.38387525	1
GOBP_GLUCOSE_IMPORT	56	0.39467975	1.4593046	0.029925186	0.25031373	1
GOBP_RESPONSE_TO_GAMMA_RADIATION	51	0.40809697	1.5108353	0.030456852	0.22105654	1
GOBP_REGULATION_OF_SYSTEMIC_ARTERIAL_BLOOD_PRESSURE	52	0.39501446	1.4567316	0.031088082	0.2488664	1
GOBP_REGULATION_OF_CYTOSOLIC_CALCIUM_ION_CONCENTRATION	238	0.27131048	1.2785102	0.031347964	0.3892958	1
GOBP_INORGANIC_ANION_TRANSPORT	93	0.34173772	1.3983495	0.031578947	0.2880333	1
GOBP_NEGATIVE_REGULATION_OF_SMOOTH_MUSCLE_CELL_PROLIFERATION	39	0.4512264	1.5125278	0.03166227	0.22030368	1
GOBP_RESPONSE_TO_OXYGEN_LEVELS	320	0.25372747	1.2256051	0.03169014	0.4450784	1
GOBP_NEGATIVE_REGULATION_OF_HORMONE_SECRETION	40	0.4402486	1.5229979	0.032098766	0.21675196	1
GOBP_VITAMIN_TRANSPORT	26	0.50607175	1.6043423	0.03241895	0.16416503	1
GOBP_POSITIVE_REGULATION_OF_RESPONSE_TO_CYTOKINE_STIMULUS	52	0.39326978	1.4369273	0.0325	0.2621337	1
GOBP_POSITIVE_REGULATION_OF_INTERLEUKIN_8_PRODUCTION	50	0.42503026	1.5402062	0.032608695	0.20458354	1
GOBP_MORPHOGENESIS_OF_AN_EPITHELIAL_SHEET	53	0.40614572	1.4901894	0.032608695	0.2320668	1
GOBP_RHYTHMIC_BEHAVIOR	28	0.49892747	1.5874543	0.032745592	0.17451696	1
GOBP_POSITIVE_REGULATION_OF_NEURON_APOPTOTIC_PROCESS	43	0.4282864	1.5082614	0.032745592	0.22266446	1
GOBP_SKELETAL_SYSTEM_DEVELOPMENT	336	0.24885519	1.2080599	0.032846715	0.45633274	1
GOBP_REGULATION_OF_GLUCOSE_IMPORT	43	0.4142272	1.4636177	0.032911394	0.24858324	1
GOBP_TYPE_2_IMMUNE_RESPONSE	29	0.46999162	1.5136541	0.032994922	0.2215268	1
GOBP_TRANSFORMING_GROWTH_FACTOR_BETA_PRODUCTION	32	0.4360725	1.4616867	0.03309693	0.24803954	1
GOBP_CYTOKINE_PRODUCTION_INVOLVED_IN_IMMUNE_RESPONSE	77	0.34759167	1.3937227	0.033942558	0.29063153	1
GOBP_AMINO_ACID_TRANSPORT	96	0.34584203	1.4291879	0.034120735	0.26676744	1
GOBP_CELLULAR_RESPONSE_TO_INSULIN_STIMULUS	188	0.29383254	1.3260791	0.034267914	0.35331744	1
GOBP_CELL_RECOGNITION	87	0.33424312	1.3619149	0.034759358	0.31486848	1
GOBP_NEGATIVE_REGULATION_OF_IMMUNE_RESPONSE	109	0.31280646	1.3255514	0.034883723	0.35247907	1
GOBP_REGULATION_OF_TRANSMEMBRANE_RECEPTOR_PROTEIN_SERINE_THR	184	0.27965757	1.2789651	0.03508772	0.3901393	1
GOBP_ACYLGLYCEROL_HOMEOSTASIS	21	0.5194638	1.5549836	0.03521127	0.19420867	1
GOBP_LOCOMOTORY_BEHAVIOR	99	0.34826314	1.4180702	0.035230353	0.2733059	1
GOBP_NEURON_DEATH	258	0.26737562	1.2680354	0.035256412	0.40427288	1
GOBP_L_GLUTAMATE_TRANSMEMBRANE_TRANSPORT	18	0.527842	1.5780894	0.035377357	0.1802619	1
GOBP_NEGATIVE_REGULATION_OF_LIPID_LOCALIZATION	38	0.43157429	1.4759239	0.035443038	0.24219804	1
GOBP_ENDOCHONDRAL_BONE_MORPHOGENESIS	41	0.4426073	1.5235417	0.035885166	0.21666226	1
GOBP_OVULATION_CYCLE_PROCESS	33	0.44445822	1.476046	0.035989717	0.24270763	1
GOBP_SMOOTH_MUSCLE_CELL_MIGRATION	64	0.37268832	1.4242294	0.035989717	0.26777044	1
GOBP_MODULATION_BY_HOST_OF_VIRAL_PROCESS	24	0.5090256	1.5642644	0.036057692	0.18700196	1
GOBP_POSITIVE_REGULATION_OF_CHEMOKINE_PRODUCTION	49	0.40914053	1.4926238	0.03617571	0.23330364	1

GOBP_OVARIAN_FOLLICLE_DEVELOPMENT	39	0.44440693	1.5038838	0.036231883	0.22310026	1
GOBP_POSITIVE_REGULATION_OF_REGULATED_SECRETORY_PATHWAY	33	0.47097206	1.5585326	0.036674816	0.19239	1
GOBP_REGULATION_OF_MORPHOGENESIS_OF_A_BRANCHING_STRUCTURE	29	0.4730198	1.5527173	0.036745407	0.1963944	1
GOBP_INTERLEUKIN_10_PRODUCTION	46	0.41959122	1.4913623	0.036939315	0.23317747	1
GOBP_REGULATION_OF_FATTY_ACID_BIOSYNTHETIC_PROCESS	34	0.4726716	1.5524071	0.037037037	0.19600248	1
GOBP_T_CELL_PROLIFERATION	154	0.29047146	1.3051513	0.037037037	0.36879575	1
GOBP_NEGATIVE_REGULATION_OF_ERK1_AND_ERK2_CASCADE	58	0.3914383	1.4528172	0.037220843	0.25308514	1
GOBP_NEURON_DEATH_IN_RESPONSE_TO_OXIDATIVE_STRESS	25	0.47611004	1.4630045	0.037406486	0.2474045	1
GOBP_REGULATION_OF_MEIOTIC_CELL_CYCLE	35	0.43033496	1.4827642	0.0375	0.23773126	1
GOBP_POSITIVE_REGULATION_OF_CD4_POSITIVE_ALPHA_BETA_T_CELL_DIFFEI	27	0.46208552	1.5006269	0.037647057	0.22552568	1
GOBP_POSITIVE_REGULATION_OF_T_HELPER_CELL_DIFFERENTIATION	18	0.5411019	1.5523087	0.03787879	0.19541539	1
GOBP_NEGATIVE_REGULATION_OF_DNA_BINDING_TRANSCRIPTION_FACTOR_A	129	0.31195375	1.345446	0.03846154	0.33273357	1
GOBP_ACTIVATED_T_CELL_PROLIFERATION	35	0.44284368	1.4683784	0.038560413	0.24680643	1
GOBP_REGULATION_OF_LYMPHOCYTE_ACTIVATION	358	0.2519036	1.2318856	0.03960396	0.436558	1
GOBP_POSITIVE_REGULATION_OF_FIBROBLAST_PROLIFERATION	38	0.433618	1.5073769	0.0397878	0.22168538	1
GOBP_NEGATIVE_REGULATION_OF_CELL_JUNCTION_ASSEMBLY	28	0.45912224	1.4704049	0.04010025	0.24504244	1
GOBP_MUSCLE_STRUCTURE_DEVELOPMENT	443	0.2370605	1.1903926	0.040145986	0.47932124	1
GOBP_PLATELET_DEGRANULATION	103	0.3227491	1.3382673	0.04054054	0.3425311	1
GOBP_CELLULAR_ION_HOMEOSTASIS	473	0.24351665	1.2207999	0.04074074	0.44808567	1
GOBP_PEPTIDYL_TYROSINE_MODIFICATION	287	0.25465563	1.2186251	0.04075235	0.4474419	1
GOBP_REGULATION_OF_STRIATED_MUSCLE_CELL_DIFFERENTIATION	57	0.38304827	1.4347119	0.040983606	0.2637596	1
GOBP_REGULATION_OF_FATTY_ACID_METABOLIC_PROCESS	69	0.368728	1.4398158	0.041162226	0.2602797	1
GOBP_PRI_MIRNA_TRANSCRIPTION_BY_RNA_POLYMERASE_II	46	0.4114461	1.4646825	0.041208792	0.24758299	1
GOBP_REGULATION_OF_ENDOCYTOSIS	167	0.29220414	1.307685	0.041533545	0.3672503	1
GOBP_CHONDROCYTE_DEVELOPMENT	23	0.5045406	1.5223132	0.041666668	0.21617806	1
GOBP_RESPONSE_TO_INTERLEUKIN_1	162	0.29522476	1.311394	0.041666668	0.3615382	1
GOBP_CHLORIDE_TRANSPORT	51	0.40936795	1.4764775	0.042105265	0.24269725	1
GOBP_POSITIVE_REGULATION_OF_ACUTE_INFLAMMATORY_RESPONSE	16	0.55895907	1.5677698	0.042394016	0.18526083	1
GOBP_NEGATIVE_REGULATION_OF_INTERFERON_GAMMA_PRODUCTION	26	0.47229737	1.5250707	0.042857144	0.21507181	1
GOBP_PROTEIN_ACTIVATION_CASCADE	18	0.52268595	1.533806	0.04347826	0.20893866	1
GOBP_NEGATIVE_REGULATION_OF_METAPHASE_ANAPHASE_TRANSITION_OF_	41	0.41722292	1.4693096	0.04411765	0.24604127	1
GOBP_REGULATION_OF_NMDA_RECEPTOR_ACTIVITY	22	0.50804996	1.5354122	0.044226043	0.20746279	1
GOBP_MUSCLE_CELL_DEVELOPMENT	114	0.31744838	1.3513032	0.044444446	0.32808298	1
GOBP_POSITIVE_REGULATION_OF_FATTY_ACID_METABOLIC_PROCESS	23	0.48458526	1.4897547	0.044554457	0.23141451	1
GOBP_MODIFIED_AMINO_ACID_TRANSPORT	22	0.5052623	1.5155094	0.04477612	0.2197206	1
GOBP_REGULATION_OF_MEMBRANE_POTENTIAL	254	0.26092422	1.2374715	0.045161292	0.43316388	1
GOBP_ANATOMICAL_STRUCTURE_HOMEOSTASIS	359	0.2447722	1.2049781	0.045454547	0.45852616	1
GOBP_REGULATION_OF_LYMPHOCYTE_DIFFERENTIATION	148	0.29506212	1.2900873	0.045584045	0.38528833	1

GOBP_RESPONSE_TO_DRUG	265	0.26179472	1.224937	0.045901638	0.44476187	1
GOBP_REGULATION_OF_SYNCYTIUM_FORMATION_BY_PLASMA_MEMBRANE_I	22	0.5169313	1.5374234	0.04622871	0.20623109	1
GOBP_NEGATIVE_REGULATION_OF_DEFENSE_RESPONSE	158	0.2949184	1.3075849	0.046376813	0.36685875	1
GOBP_REGULATION_OF_LEUKOCYTE_PROLIFERATION	187	0.28061974	1.2731317	0.04658385	0.39715132	1
GOBP_BEHAVIOR	326	0.25575623	1.2428783	0.04682274	0.42691338	1
GOBP_REGULATION_OF_BMP_SIGNALING_PATHWAY	63	0.3628023	1.3759134	0.046875	0.30422214	1
GOBP_NEGATIVE_REGULATION_OF_ANION_TRANSMEMBRANE_TRANSPORT	25	0.4804879	1.4936552	0.047058824	0.2334509	1
GOBP_REGULATION_OF_MACROPHAGE_DIFFERENTIATION	17	0.53383994	1.5301964	0.047732696	0.21150734	1
GOBP_NEGATIVE_REGULATION_OF_INFLAMMATORY_RESPONSE	101	0.32762104	1.3489784	0.048257373	0.33112124	1
GOBP_NEGATIVE_REGULATION_OF_CHEMOKINE_PRODUCTION	18	0.54455674	1.5421314	0.04842615	0.20351543	1
GOBP_REGULATION_OF_RECEPTOR_SIGNALING_PATHWAY_VIA_STAT	60	0.369896	1.3930354	0.048469387	0.29053694	1
GOBP_MEIOTIC_CELL_CYCLE	171	0.28194922	1.269467	0.048484847	0.402304	1
GOBP_CELLULAR_RESPONSE_TO_HORMONE_STIMULUS	470	0.22657108	1.1493671	0.048507463	0.52446216	1
GOBP_SIGNALING_RECEPTOR_LIGAND_PRECURSOR_PROCESSING	19	0.52474	1.5067701	0.048611112	0.22102521	1
GOBP_CELL_KILLING	100	0.3226498	1.3458236	0.048710603	0.333259	1
GOBP_REPLICATION_FORK_PROCESSING	34	0.44972286	1.4786587	0.048843186	0.23992617	1
GOBP_REGULATION_OF_WOUND_HEALING	95	0.3262658	1.3236412	0.049479168	0.3537076	1
GOBP_NEGATIVE_REGULATION_OF_LIPID_STORAGE	19	0.54780394	1.6011823	0.04987531	0.16387545	1
GOBP_MAST_CELL_ACTIVATION	47	0.3874453	1.4032491	0.04987531	0.28288406	1
GOBP_POSITIVE_REGULATION_OF_LIPID_BIOSYNTHETIC_PROCESS	60	0.36644855	1.3866554	0.05	0.29249242	1

Kramer et al. Supplemental Table 4

**Oligonucleotides (5'->3')**		
**Genotyping PCR**		
Lyz2-cre	CCCAGAAATGCCAGATTACG	CTTGGGCTGCCAGAATTTCTC
Ctnnb1Ex3Δ	CATTGCGTGGACAATGGCTACTCA	CTAAGCTTGGCTGGACGTAAACTC
ROSA26-EYFP	AGGGCGAGGAGCTGTTCA	TGAAGTCGATGCCCTTCAG
**CUT&RUN qPCR**		
Tnf -408	CCCCCATGGATGTCCCATTT	TGTAGAAAGACCATGCCTGTG
Axin2 -340	GCGGATCTAGCTCCTACCAG	TCAAATCAAACACGTTCCTACCTT
**qRT-PCR**	**Forward**	**Reverse**
Ppia	GGCAAATGCTGGACCAAAC	CATTCCTGGACCCAAAACG
Axin2	GCTGCGCTTTGATAAGGTCC	GCAATCGGCTTGGTCTCTCT
Ccnd1	CGCCCTCCGTATCTTACTTC	AAGCGGTCCAGGTAGTTCAT
Cd63	CAGGTTAGGAGTGTAAGGCCG	ATGGCGATCAATCCCACTGC
Lyz1	GCAGTGCTCTGCTGCAGGAT	GTCAGACTCCGCAGTTCCGA
Pla2g7	AGGCTGTATGCTCAACCCAC	TTTGATGTTCTGGTCACTGCAC
Mfge8	GTGCCCTGTGGGCTACTC	GTATTGGGGACGGCTGTG
Abca1	GGTTTGGAGATGGTTATACAATAGTTGT	CCCGGAAACGCAAGTCC
Gpnmb	GAAGCCAGCATCTCAGGTTC	TGAACACCGACCCAGTTTT
C3ar1	CAGGCAAGGGATTACTTTTGG	TGTGAGGACATTAGGAGGCTTTCC
Mmp12	TTTCTTCCATATGGCCAAGC	GGTCAAAGACAGCTGCATCA
Tnf	GGTCTGGGCCATAGAACTGA	CAGCCTCTTCTCATTCCTGC
Il1a	TTCTATGATGCAAGCTATGGCTCA	CGGCTCTCCTTGAAGGTGA
Timd4	CGTATAGAGGTGCCTGGCTGGTTC	GGGCACGTGGTCACTGCTGTAC
Lyve1	TGGTGTTACTCCTCGCCTCT	TTCTGCGCTGACTCTACCTG
Adgre1	GAAGCATCCGAGACACACAC	TTGTGGTTCTGAACAGCACG
Siglecf	CAGGGACGTACTTCTTCAGATTGG	GGGTAGATGTGACTTGGATGTTAGG
Plet1	CACTATGGCTAACGTCTCTGG	CTGTCGTCCTCCTTCACTG
Fabp5	TGAAAGAGCTAGGAGTAGGACTG	CTCTCGGTTTTGACCGTGATG
Net1	ACATTCTCGTGAACTGGTTA	GCTGGAGGAAGTCTTGGA
Trp2	CCTGAATGGGACCAATGCCT	AGGCATCTGTGGAAGGGTTG
Pmel	GGGGATGCATTTGAGCTGAC	CTGGCACCCTGGTGATGAAA

Kramer et al. Supplemental Table 5

Flow Cytometry Antibodies	Source	Identifier
**Analysis of Lung Active β-catenin**		
Anti mouse CD16/CD32 FC	BD Bioscience	Cat # 553142
BUV395 anti mouse CD45	BD Bioscience	Cat # 564616
AlexaFluor 488 anti mouse Non-phospho (Active) β-Catenin (Ser45)	Cell Signaling Technology	Cat # 70034
**Analysis of the Lung Microenvironment**		
Anti mouse CD16/CD32 FC	BD Bioscience	Cat # 553142
PerCP-Cy5.5 anti mouse CD64	Biolegend	Cat # 139307
BV711 anti mouse CD11b	Biolegend	Cat # 101241
BV510 anti mouse MHCII	Biolegend	Cat # 107635
PE anti mouse CD24	Biolegend	Cat # 101807
APC anti mouse CD45	Biolegend	Cat # 103112
PE-Cy7 anti mouse CD11c	Biolegend	Cat # 117318
Pacific Blue anti mouse Ly6G	Biolegend	Cat # 127612
AlexaFluor 700 anti mouse Ly6C	Biolegend	Cat # 128024
**Analysis of the Tumor Microenvironment**		
Anti mouse CD16/CD32 FC	BD Bioscience	Cat # 553142
APC anti mouse CD45	Biolegend	Cat # 103112
BV711 anti mouse CD11b	Biolegend	Cat # 101241
Pacific Blue anti mouse Ly6G	Biolegend	Cat # 127612
AlexaFluor 700 anti mouse Ly6C	Biolegend	Cat # 128024
PE-Cy7 anti mouse CD11c	Biolegend	Cat # 117318
BUV395 anti mouse CD3	BD Bioscience	Cat # 563565
FITC anti mouse CD4	BD Pharmigen	Cat # 553047
BV650 anti mouse CD8	Biolegend	Cat # 100742
BV605 anti mouse F4/80	Biolegend	Cat # 123133
**Analysis of the Neonatal Liver-Derived Macrophages**		
Anti mouse CD16/CD32 FC	BD Bioscience	Cat # 553142
PE-Cy7 anti mouse CD11c	Biolegend	Cat # 117318
BV605 anti mouse F4/80	Biolegend	Cat # 123133
**Fluorsence Assisted Cell Sorting**		
Anti mouse CD16/CD32 FC	BD Bioscience	Cat # 553142
APC anti mouse CD45	Biolegend	Cat # 103112
Pacific Blue anti mouse Ly6G	Biolegend	Cat # 127612
PE-Cy7 anti mouse CD11c	Biolegend	Cat # 117318

CUT&RUN		
β-Catenin (D10A8) XP	Cell Signaling Technology	Cat # 8480
Rabbit (DA1E) mAb IgG XP Isotype Control	Cell Signaling Technology	Cat # 66362

In Vivo Antibody Treatments		
InVivoMAb anti-mouse TNFα	BioXCell	Cat # BE0058

In Vivo Alveolar Macrophage Depletion		
Clodronate liposomes (neutral)	FormuMax	Cat # F70101C-N

Chemicals, Peptides, and Recombinant Proteins		
Mouse GM-CSF	Peprotech	Cat # 315-03
Mouse M-CSF	Peprotech	Cat # 315-02
Mouse Wnt-3a	Peprotech	Cat # 315-20
Lithium Chloride	Sigma	Cat # L9650
Lipopolysaccharides (LPS)	Sigma	Cat # L2630
Cellstripper	Corning	Cat # 25-056-CI

Dulbecco's PBS	Corning	Cat # 20-031-CV
FBS	Corning	Cat # 35-011-CV
EDTA	Corning	Cat # 46-034-CL
Trypsin EDTA 1X	Corning	Cat # 25-053-CL
Trypan Blue Solution, 0.4%	Gibco	Cat # 15250061
DAPI	Thermofisher	Cat # 62248
ACK Lysis Buffer	Gibco	Cat # A1049201
Matrigel	Corning Inc.	Cat # 356234
L-glutamine	Gibco	Cat # 25030081
Sodium Pyruvate	Gibco	Cat # 11360070
2-mercaptoethanol	Gibco	Cat # 31350010
Pen/Strep	Gibco	Cat # 10378016
Collagenase/Hyaluronidase	Stemcell	Cat # 07912
Live/Dead Blue	ThermoFisher	Cat # L23105
SYBR Green PCR Master Mix	Applied Biosystems	Cat # 4309155
D-luciferin	GoldBio	Cat # Luck-100
TriTrack DNA Loading Dye (6X)	ThermoFisher	Cat # R1161
GeneRuler 100 bp DNA Ladder	ThermoFisher	Cat # SM0243
DirectPCR Lysis Reagent (Mouse Tail)	Viagen Biotech	Cat # 102-T
Methanol, 99.9%	ThermoFisher	Cat # 176845000
Crystal violet solution	Sigma	Cat # V5265

**Critical Commercial Assays**		
RNeasy Mini Kit	Qiagen	Cat # 74104
iScript cDNA synthesis kit	Bio-Rad	Cat # 1708890
ELISA MAX™ Deluxe Set Mouse TNF-α	Biolegend	Cat # 430904
CUT&RUN Assay Kit	Cell Signaling Technology	Cat # 86652
DNA Purification Kit	Cell Signaling Technology	Cat # 14209
FoxP3 Staining Buffer Set	Miltenyi Biotec	Cat # 130-093-142

**Depositied Data**		
RNA-Seq Data	This paper	GSE200508
scRNA-Seq Data	Zhu et al. 2021	GSE164793
scRNA-Seq Data	Travaglini et al. 2020	EGAS00001004344

**Experimental Models: Organisms/ Strains**		
C57BL/6 Mice	Charles River Laboratories	Strain Code 556
C57BL/6 Lyz2-cre+/+ Mice	The Jackson Laboratory	Strain Code 004781
C57BL/6 ROSA26-EYFP+/+ Mice	The Jackson Laboratory	Strain Code 006148
C57BL/6 Ctnnb1Ex3Δ/Ex3Δ Mice	Harada et al. 1999	

**Experimental Models: Cell Lines**		
B16F10	ATCC	Cat # CLR-6475
E0771.ML-1	Liu et al., 2021	
LLC-luc	Dr. Pamela Hershberger, Buffalo, NY	

Supplemental Table Legends

Supplemental Tables 1-3 Related to Figure 4 & Supplemental Figure 4. Complete lists of significantly, (adjusted) p-values < 0.05, differentially expressed genes in *Lyz2-Cre*$^{+/+}$*YFP*$^{+/-}$ *Ctnnb1*$^{Ex3\Delta/wt}$ versus *Lyz2-Cre*$^{+/+}$*YFP*$^{+/-}$ non-tumor bearing AMs (Supplemental Table 1), and pathways upregulated in the former population of AMs identified by Gene Set Enrichment Analysis with (NOM) p-values < 0.05 (Supplemental Table 2 & 3).

Supplemental Table 4. Primer pairs utilized for murine genotyping, CUT&RUN, and qRT-PCR experiments.

Supplemental Table 5. Key resources, antibodies, reagents, cell and animal lines used in this study.
